# Putting cyaphide in its place: determining the donor/acceptor properties of the κ*C*-cyaphido ligand†

**DOI:** 10.1039/d3sc01126g

**Published:** 2023-04-06

**Authors:** Eric S. Yang, Emma Combey, Jose M. Goicoechea

**Affiliations:** a Department of Chemistry, University of Oxford, Chemistry Research Laboratory 12 Mansfield Rd. Oxford OX1 3TA UK; b Department of Chemistry, Indiana University 800 East Kirkwood Ave. Bloomington Indiana 47405 USA jgoicoec@iu.edu

## Abstract

The synthesis of group 9 pyridine–diimine complexes M(^Dipp^PDI)X and [M(^Dipp^PDI)L]^+^ (M = Co, Rh; ^Dipp^PDI = 1,1′-(pyridine-2,6-diyl)bis(*N*-(2,6-diisopropylphenyl)ethan-1-imine); X = CP^−^, CCH^−^; L = CO, ^*t*^BuNC) bearing a series of strong-field ligands, including the cyaphide ion (C≡P^−^), is reported. A combined experimental and computational comparative study of the group 9 PDI cyaphide complexes Co(^Dipp^PDI)(CP) and Rh(^Dipp^PDI)(CP), as well as the N-heterocyclic carbene (NHC) gold(i) cyaphide complex Au(IDipp)(CP) (IDipp = 1,3-bis(2,6-diisopropylphenyl)imidazol-2-ylidene), reveals the σ donor and π acceptor properties of the κ*C*-cyaphido ligand, and allow us to suggest a position for this ion in the spectrochemical series.

## Abstract 2

The σ-donor and π-acceptor properties of the cyaphide ion (C≡P^–^) are compared to other well-established strong-field ligands allowing us to assess the relative position of this unique ion in the spectrochemical series.

## Graphical abstract

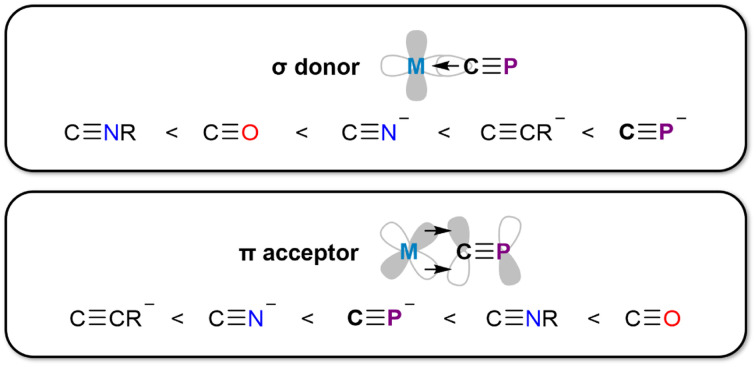

## Introduction

The cyanide ion (C≡N^−^) is found in coordination compounds across many areas of chemistry, ranging from biological enzyme cofactors to bespoke magnetic materials and catalysts.^1–4^ In the end-on κ*C* coordination mode, the cyanide ion is an archetypal strong field ligand, a good σ donor and moderate π acceptor. These properties are critical to the utility of the cyanido ligand; its uncommon ability to withdraw π electron density despite its negative charge makes it a particularly useful tool in coordination chemistry.^5–7^

The cyaphide ion, C≡P^−^, is a phosphorus-containing analogue of the cyanide ion. Unlike the cyanide ion, it is rare, with relatively few known examples of cyaphido metal complexes having been reported to date.^8–12^ As a consequence, its ligand properties are still poorly understood. However, the potential for even higher π accepting character compared to cyanide has made the cyaphide ion an attractive candidate for use as a bridging ligand in magnetic materials, where its low-lying C≡P π* orbitals should facilitate more effective super-exchange between open-shell metal centers.^6^ Conceptually, the cyaphide ion is most closely related to two isolobal congeners, the cyanide (C≡N^−^) and acetylide (C≡CH^−^) ions. While both the cyanido and acetylido ligands are known to be good σ donors, the acetylido ligand has much reduced π withdrawing character, acting as a borderline π acceptor or donor.^13,14^

There is scant experimental evidence available with which to probe the electronic properties of the cyaphido ligand. The only systematic study of κ*C*-cyaphido complexes conducted to date exclusively probed the effect of *trans*-ligated acetylides on ruthenium(ii) cyaphido complexes,^15^ leaving the donor/acceptor properties of the cyaphide ligand itself still somewhat of a mystery.

**Fig. 1:**
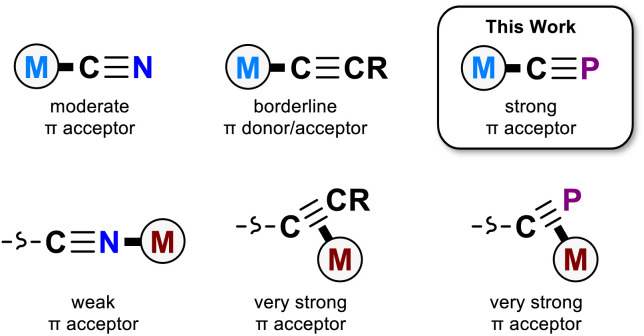
Coordination chemistry of the cyanido, acetylido, and cyaphido ligands.

Recently, we reported a magnesium(ii) cyaphide complex Mg(^Dipp^NacNac)(dioxane)(CP) (Mg_CP_; ^Dipp^NacNac = CH{C(CH_3_)N(Dipp)}_2_ and Dipp = 2,6-diisopropylphenyl) which, by analogy to Grignard reagents, can be used to transfer the cyaphide ion to the coordination sphere of other metal centers using simple salt metathesis reactions.^16^ This has enabled synthetic access to many metal cyaphide coordination complexes which can be studied to better understand the properties of this anion. To this end, we recently used the gold(i) cyaphide complex Au(IDipp)(CP) (Au_CP_, IDipp = 1,3-bis(diisopropylphenyl)-imidazol-2-ylidene) to prepare heterometallic complexes featuring the cyaphide ion as a bridging ligand, revealing the electrophilic, π withdrawing nature of the cyaphide ion in side-on η^2^-coordination to metal centers (Fig. 1).^17^

As traditional methods for probing ligand donor strength are currently inaccessible for the cyaphido ligand – due to a lack of [M(CP)_6_]^*x*−^ or [M(CP)_4_]^*x*−^ type complexes – in this study we focus on the use of both NMR spectroscopic and crystallographically determined bond metric data to do so. This allows us to deconvolute the donor/acceptor properties of the terminal κ*C*-cyaphido ligand through comparative studies of three cyaphido metal complexes, the gold(i) complex (IDipp)Au(CP) (Au_CP_), the cobalt(i) complex Co(^Dipp^PDI)(CP) (Co_CP_; ^Dipp^PDI = 1,1′-(pyridine-2,6-diyl)bis(*N*-(2,6-diisopropylphenyl)ethan-1-imine)), and the novel rhodium(i) complex Rh(^Dipp^PDI)(CP) (Rh_CP_). Au_CP_ is compared to other known gold(i) complexes Au(IDipp)X and [Au(Dipp)L]^+^ (Au_X_ and Au_L_^+^; where X and L are used to describe anionic and neutral ligands, respectively, in accordance with the Covalent Bond Classification Method).^18^ Analysis of the gold(i) complexes allows us to determine the *σ donor properties* of the cyaphide ion. A systematic comparison of Co_CP_ and Rh_CP_ with both known and novel M(^Dipp^PDI)X and [M(^Dipp^PDI)L]^+^ complexes (M_X_ and M_L_^+^, M = Co, Rh) allows us to probe the *π accepting character* of the terminally bonded cyaphido ligand.

## Results and discussion

**Fig. 2:**
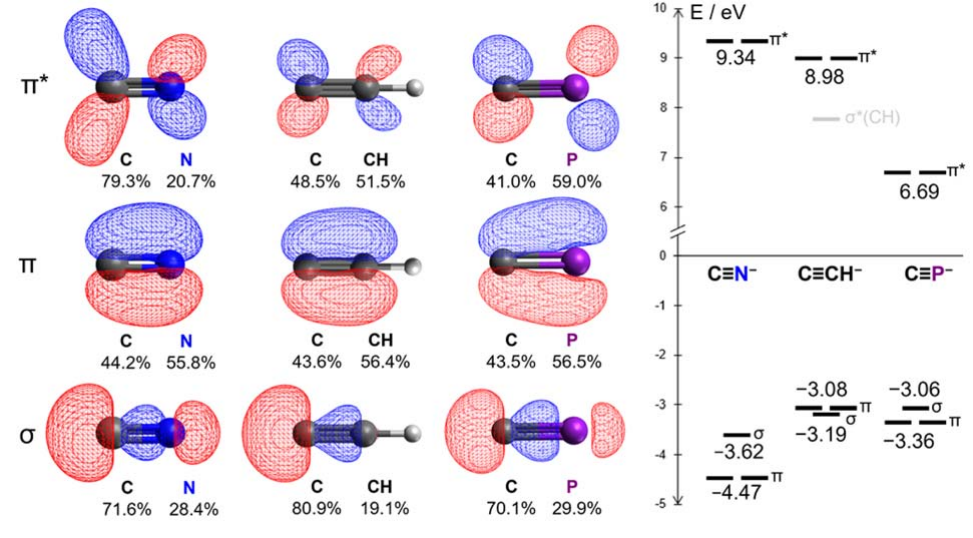
Kohn–Sham DFT frontier orbitals of the cyanide, acetylide, and cyaphide ions.

From a theoretical standpoint, the ligand properties of the cyaphide ion are not immediately obvious. Its 2p–3p C≡P π bonds result both in low energy antibonding π* orbitals (6.69 eV, *cf.* CN^−^: 9.34 eV, CCH^−^: 8.98 eV) as well as high energy bonding π orbitals (−3.36 eV, *cf.* CN^−^: −4.47 eV, CCH^−^: −3.08 eV), increasing both π withdrawing and π donating ability relative to the cyanide ion. Moreover, the lower electronegativity of phosphorus relative to carbon results in the polarization of the π* orbital away from the coordinating carbon atom (41.0% C, *cf.* CN^−^: 79.3% C, CCH^−^: 48.5% C), in principle worsening Md–π* orbital overlap (Fig. 2, see ESI† for full computational details).

It has been previously demonstrated that the N-heterocyclic carbene (NHC) carbenoid ^13^C{^1^H} NMR chemical shift (^13^C_NHC_) is sensitive to the σ basicity of *trans*-coordinated co-ligands.^19^ A similar trend can be observed in the case of gold(i) Au(IDipp)X and [Au(Dipp)L]^+^ complexes (Au_X_ and Au_L_^+^) which exhibit ^13^C_NHC_ chemical shifts over a wide frequency range (∼50 ppm) depending on the nature of the ligand *trans* to the carbene (Table S9†).^16,17,20–28^ Weak σ donors (such as acetonitrile) give rise to low ^13^C_NHC_ chemical shifts (*e.g.*Au_NCMe_^+^: ^13^C_NHC_ = 166.0 ppm), whereas strong donors (*e.g.* boryls) give rise to high shifts (*e.g.*Au_BPin_: ^13^C_NHC_ = 216.7 ppm). The solid-state C_NHC_–Au bond lengths in such complexes are less sensitive to the nature of the *trans*-ligand, although they follow a similar trend, apart from strong π donors (such as chloride and hydroxide ligands) which deviate slightly. This is consistent with previous theoretical studies on gold(i) NHC complexes: while electrostatic and σ donation effects dominate for NHC–Au^I^ bonds,^29^ π donating co-ligands have been shown to enable more significant Au^I^–NHC π backbonding.^30^

**Fig. 3:**
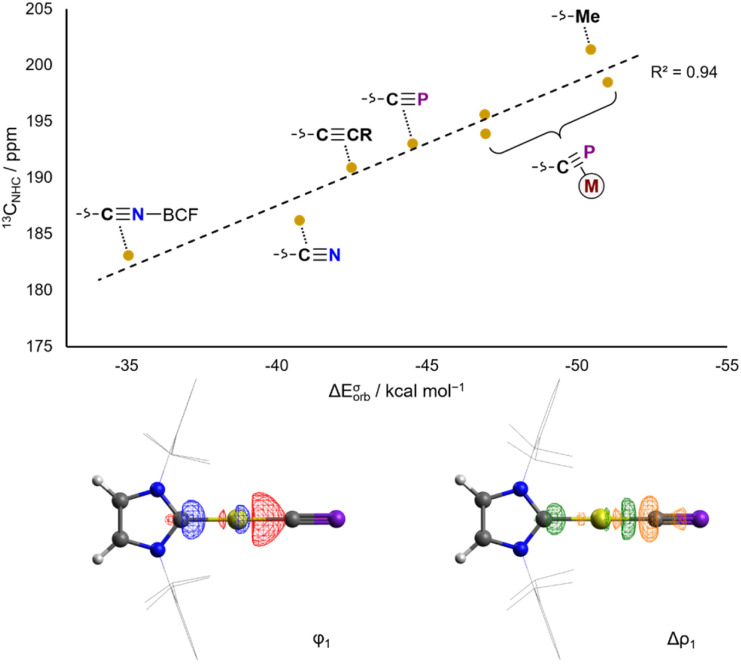
Correlation of calculated ETS-NOCV σ donation energies with experimental ^13^C_NHC_ NMR spectroscopic data for Au_X_ complexes (top). Isosurfaces for the σ bonding NOCV and difference density in Au_CP_ (bottom).

EDA-NOCV analysis of the L–Au bond in the aforementioned complexes allows for the theoretical examination of σ donor and π acceptor orbital interactions. Experimental ^13^C_NHC_ chemical shifts correlate very well with calculated ETS-NOCV σ donation energies (Fig. 3), and conversely do not correlate well with ETS-NOCV π backdonation energies (Fig. S50†). Thus the ^13^C_NHC_ chemical shift in Au(IDipp)X and [Au(Dipp)L]^+^ compounds can be taken as a measure of the σ donating ability of the *trans* ligand.

In Au_CP_, the ^13^C_NHC_ chemical shift is 193.0 ppm, in a very similar range to other sp-hybridized carbanions such as cyanide and phenylacetylide (Au_CN_: ^13^C_NHC_ = 186.2 ppm; Au_CCPh_: ^13^C_NHC_ = 190.0 ppm). This is also reflected computationally in the ETS-NOCV σ donor energies: the cyaphide complex has a Δ*E*^σ^_orb_ value of −44.5 kcal mol^−1^ compared to −40.8 kcal mol^−1^ for cyanide and −42.5 kcal mol^−1^ for acetylide. These data show that the cyaphido ligand has a very similar σ basicity to closely related sp-carbanionic ligands, though slightly higher due to its higher energy HOMO and/or the lower electronegativity of phosphorus resulting in higher charge density at carbon.

The same analysis can be performed for the previously reported hetero- bi- and tri-metallic bridging cyaphide complexes {Au(IDipp)}{Ni(^Me^I^i^Pr)_2_}(μ-CP), {Au(IDipp)}{Rh(Cp*)(PMe_3_)}(μ-CP), and {Au(IDipp)}{Rh(Cp*)(PMe_3_)}{W(CO)_5_}(μ-CP).^17^ The bimetallic complexes give rise to higher ^13^C_NHC_ chemical shifts (^13^C_NHC_ = 198.5 and 195.6 ppm, for the nickel and rhodium complexes, respectively), showing that the net electron-withdrawing nature of the η^2^ cyaphido-metal interaction results in heightened σ donor ability at carbon, which is also reflected in larger calculated Δ*E*^σ^_orb_ energies (Δ*E*^σ^_orb_ = −51.0 and −46.9 kcal mol^−1^, respectively). Coordination of a third metal to the cyaphide ion *via* the phosphorus lone pair results in a moderate reduction in σ donor ability, with the trimetallic complex exhibiting a ^13^C_NHC_ chemical shift of 193.9 ppm.

Whereas Au_CP_ allows for the study of the σ donor properties of the κ*C*-cyaphido ligand, the group 9 cyaphido complexes Co(^Dipp^PDI)(CP) (Co_CP_) and Rh(^Dipp^PDI)(CP) (Rh_CP_) are sensitive to its π donor/acceptor character. The tridentate pyridine diimine (PDI) ligand framework has a low lying π* orbital that gives rise to strong π accepting character and redox non-innocence. Population of this π* orbital by π backdonation or reduction results in measurable changes in the solid-state bond metrics of the PDI ligand, summarized in the parameter *δ*(PDI),^31^ which allows for convenient assessment of the electronic structure of metal PDI complexes (see Tables S10 and S11†). An NMR study of the purple, square-planar cobalt complexes Co(^Dipp^PDI)H (Co_H_), Co(^Dipp^PDI)Me (Co_Me_),^32^ and Co(^Dipp^PDI)Cl (Co_Cl_),^33^ has previously shown that they consist of cobalt(ii) centers ligated by reduced (^Dipp^PDI)˙^−^ radical anions.^34^ Notably absent from this study were ligands with appreciable π accepting ability. Indeed it has been noted that the cationic dinitrogen complex [Co(^Dipp^PDI)(N_2_)][B(Me)(C_6_F_5_)_3_] has a markedly different *δ*(PDI) value than for the aforementioned complexes (0.132(9); *cf.* 0.090(9)–0.098(7) for Co_H_, Co_Me_, and Co_Cl_) and is a deep blue color instead of purple. This suggests it is better described as having a cobalt(i) center and neutral ^Dipp^PDI ligand.^31^

**Scheme 1:**
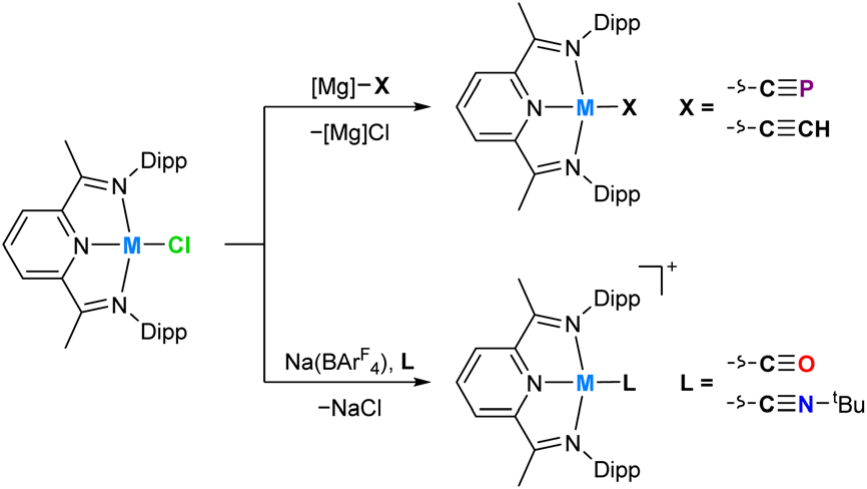
Synthesis of M_X_ (M = Co, Rh; X = CP^−^ CCH^−^) and M_L_^+^ complexes (M = Co, Rh; L = CO, CN^*t*^Bu).

**Fig. 4:**
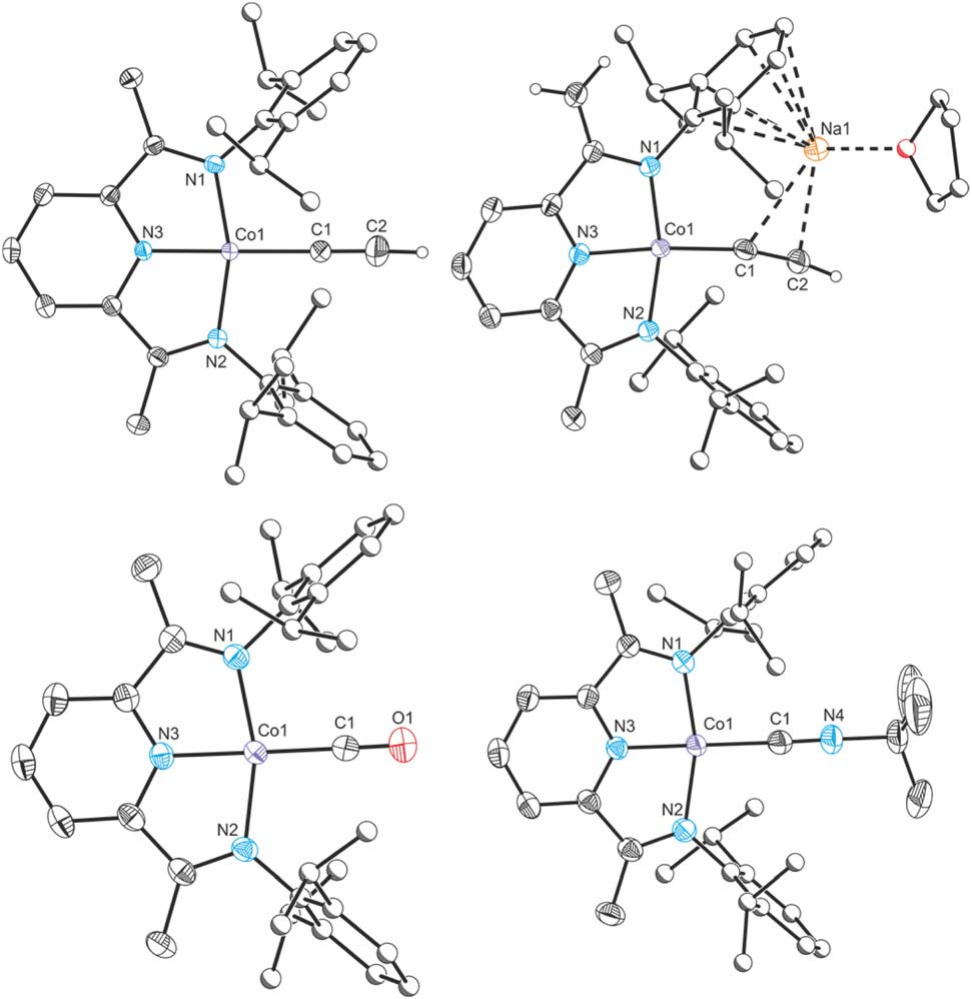
Clockwise from top left: single crystal X-ray structures of complexes Co_CCH_, Na(THF)Co(PIEA)(CCH), Co_CNBu_^+^ and Co_CO_^+^. Thermal ellipsoids set at 50% probability level. Hydrogen atoms (except for acetylide and terminal alkene protons) removed for clarity. Atoms of Dipp groups pictured as spheres of arbitrary radius.

In order to provide additional useful data points against which the cobalt(i) cyaphide complex Co_CP_ can be compared, several complexes with ligands of varying π acceptor character (Co_X_ and Co_L_^+^) were prepared and characterized.‡‡See ESI for full experimental details, analytical and computational data. The purple cobalt acetylide complex Co(^Dipp^PDI)(CCH) (Co_CCH_) was prepared by salt metathesis of Co_Cl_ with ethynylmagnesium chloride (Scheme 1). Its solid-state structure was determined by single crystal X-ray diffraction (Fig. 4), showing a square-planar cobalt center with a N_py_–Co bond length of 1.812(2) Å and a *δ*(PDI) value of 0.098(7) (Table S10†). If the reaction is performed with an excess of sodium acetylide instead of the Grignard reagent, a mixture of products forms, from which Na(THF)Co(PIEA)(CCH) (PIEA = pyridine–imine–enamine) can be isolated, containing a deprotonated PDI ligand and a sodium cation held in a close ion pair by π interactions with arene and acetylide moieties.

Cobalt carbonyl [Co(^Dipp^PDI)(CO)][BAr^F^_4_] (Co_CO_^+^, BAr^F^_4_^−^ = tetrakis(3,5-bis(trifluoromethyl)phenyl)borate) and *tert*-butyl isocyanide [Co(^Dipp^PDI)(CN^*t*^Bu)][BAr^F^_4_] (Co_CNBu_^+^) complexes were synthesized by halide abstraction with Na(BAr^F^_4_) in 1,2-difluorobenzene (1,2-DFB), in the presence of CO gas or ^*t*^BuNC, respectively. Both complexes are blue and have solid-state structures with N_py_–Co bond lengths of 1.847(2) Å (Co_CO_^+^) and 1.819(2) Å (Co_CNBu_^+^), and *δ*(PDI) values of 0.144(7) (Co_CO_^+^) and 0.121(4) (Co_CNBu_^+^). If two equivalents of ^*t*^BuNC are used the reaction results in a different product, [Co(^Dipp^PDI)(CN^*t*^Bu)_2_][BAr^F^_4_] (Co_2CNBu_^+^), with a five-coordinate square-pyramidal cobalt center (see ESI†).

The cobalt cyanide complex Co(^Dipp^PDI)(CN) (Co_CN_) was also targeted, although attempts to prepare it *via* several methods were unsuccessful, including salt metathesis with KCN or Me_3_SiCN, and halide abstraction in the presence of [Bu_4_N]CN. This is likely due to the potential for redox disproportionation. The reaction of the cobalt(ii) complex Co(^Dipp^PDI)Cl_2_ with sodium cyanide was previously shown to lead to disproportionation, forming Co(^Dipp^PDI)(CN)_3_ and other unidentified byproducts.^35^ An alternative decomposition pathway could involve deprotonation of the PDI ligand by the Brønsted basic cyanide ion (as observed for the reaction of Co_Cl_ with sodium acetylide; *vide supra*).

**Fig. 5:**
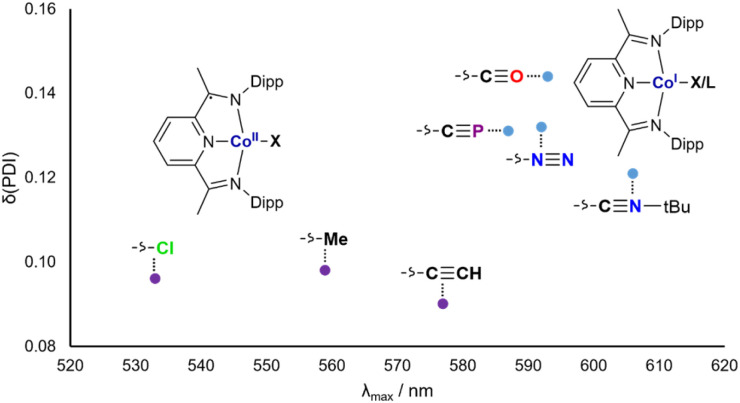
UV-vis absorbance maxima (*λ*_max_) plotted against *δ*(PDI) for Co_X_ and Co_L_^+^.

The cobalt cyaphide complex Co_CP_ is blue, with a N_py_–Co bond length of 1.825(3) Å and a *δ*(PDI) value of 0.131(9). From these data, it is clear that Co_X_ and Co_L_^+^ (X and L are CP^−^, CCH^−^, Me^−^, N_2_, CO, CN^*t*^Bu, and Cl^−^) can be separated into two groups with different electronic structures. The purple complexes with low *δ*(PDI) values are best described as Co^2+^(^Dipp^PDI)˙^−^ systems, whereas the blue complexes with high *δ*(PDI) values are better described as Co^1+^(^Dipp^PDI). In order to quantify this, UV-vis spectra for all complexes were measured. As expected, the absorbance maxima (*λ*_max_) relate discontinuously with *δ*(PDI), with low *λ*_max_ corresponding to low *δ*(PDI) and *vice versa* (Fig. 5).

The group of blue Co^1+^(^Dipp^PDI) complexes with higher *λ*_max_ and *δ*(PDI) values all feature π accepting ligands, with the notable inclusion of the cyaphide ion. This can be rationalized by considering the effect of the ligand on the Co(^Dipp^PDI) fragment: when it is primarily electron donating, the electron rich Co(^Dipp^PDI) fragment favors the reduced ^Dipp^PDI state, whereas when the ligand is π withdrawing, the less electron rich Co(^Dipp^PDI) fragment favors the neutral ^Dipp^PDI state.

**Fig. 6:**
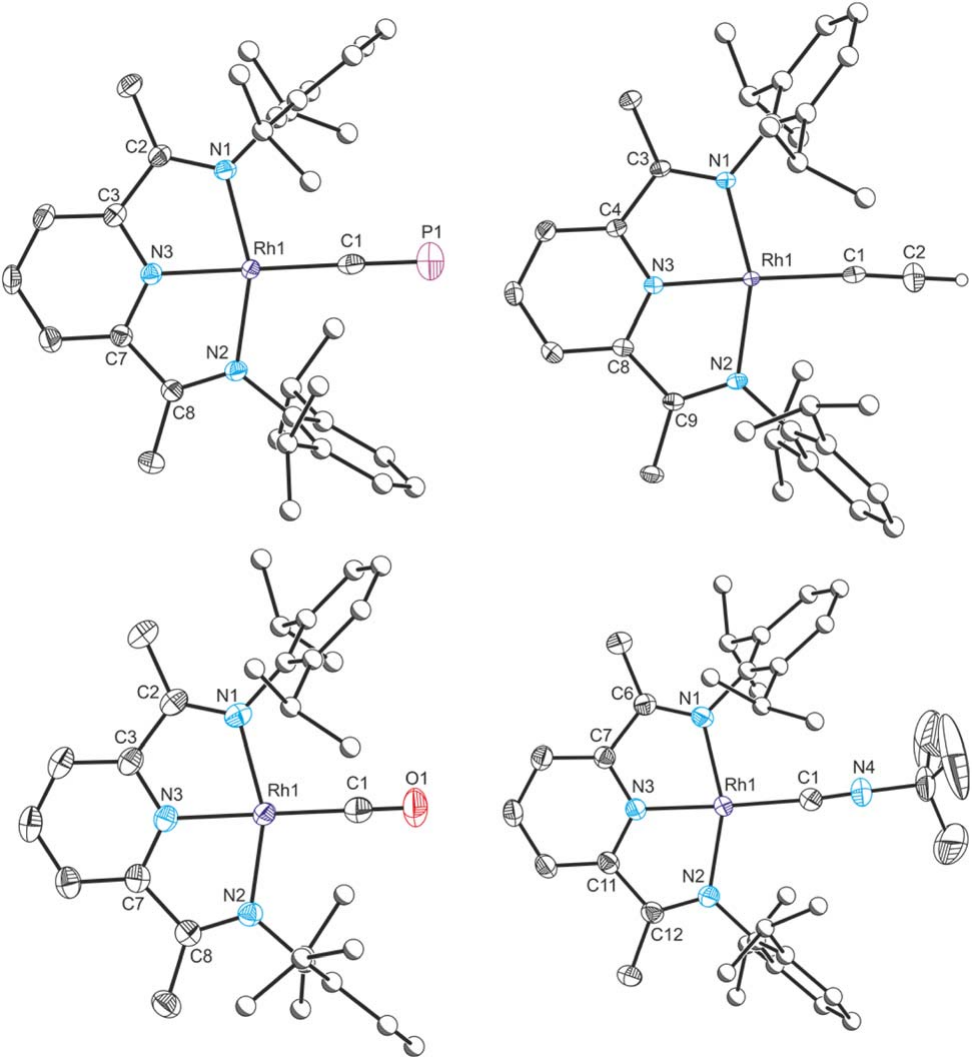
Clockwise from top left: single crystal X-ray structures of complexes Rh_CP_, Rh_CCH_, Rh_CNBu_^+^ and Rh_CO_^+^. Thermal ellipsoids set at 50% probability level. Hydrogen atoms (except for acetylide protons) removed for clarity. Atoms of Dipp groups pictured as spheres of arbitrary radius. Selected bond distances (Å) and angles (°) for Rh_CP_: Rh1–C1 1.969(3), C1–P1 1.542(4), Rh1–N1 2.019(2), Rh1–N2 2.023(2), Rh1–N3 1.942(2), N1–C2 1.309(4), C2–C3 1.470(4), C3–N3 1.360(4), N3–C7 1.357(4), C7–C8 1.468(4), C8–N2 1.315(4); C1–Rh1–N1 101.06(11), C1–Rh1–N2 101.41(11), C1–Rh1–N3 179.24(14), N1–Rh1–N2 157.48(10), N1–Rh1–N3 78.92(10), N2–Rh1–N3, 78.59(10).

In order to better quantify the π accepting ability of the cyaphido ligand, the heavier group 9 ^Dipp^PDI complexes Rh(^Dipp^PDI)X and [Rh(^Dipp^PDI)L]^+^ (Rh_X_ and Rh_L_^+^) were prepared, in which the much lower relative favorability of the rhodium(ii) oxidation state should preclude the redox non-innocence of the PDI ligand. Addition of Rh(^Dipp^PDI)Cl^36^ (Rh_Cl_) to a toluene solution of the cyaphide transfer reagent Mg_CP_ results in the formation of the novel rhodium(i) cyaphide complex Rh(^Dipp^PDI)(CP) (Rh_CP_) after 3 days. Rh_CP_ exhibits a doublet in its ^31^P{^1^H} NMR spectrum at 251.4 ppm (^2^*J*_P–Rh_ = 6 Hz) corresponding to the cyaphide phosphorus atom and a doublet of doublets in its ^13^C{^1^H} NMR spectrum at 264.15 ppm (^1^*J*_C–Rh_ = 54.8 Hz, ^1^*J*_C–P_ = 22.9 Hz) corresponding to the cyaphide carbon atom. The C≡P vibrational stretching frequency was measured to be 1334 cm^−1^ by Raman spectroscopy (*cf.* 1306 cm^−1^ for Co_CP_). Its solid-state structure was determined by X-ray crystallography (Fig. 6), revealing a square-planar rhodium center, a C–P bond length of 1.542(4) Å (*cf.* 1.506(4) Å for Co_CP_), a N_py_–Rh bond length of 1.943(2) Å, and a *δ*(PDI) value of 0.134(3).

The rhodium(i) acetylide complex Rh(^Dipp^PDI)(CCH) (Rh_CCH_) could likewise be prepared by salt metathesis with ethynylmagnesium chloride. Single crystal X-ray crystallography confirms its structure, with a N_py_–Rh bond length of 1.926(2) Å and a *δ*(PDI) value of 0.124(2).

As with Co_CO_^+^ and Co_CNBu_^+^, cationic rhodium(i) carbonyl and isocyanide complexes could be prepared by halide abstraction. Reaction of 1,2-DFB solutions of Rh_Cl_ and Na(BAr^F^_4_) with CO or ^*t*^BuNC resulted in the formation of [Rh(^Dipp^PDI)(CO)][BAr^F^_4_] (Rh_CO_^+^) and [Rh(^Dipp^PDI)(CN^*t*^Bu)][BAr^F^_4_] (Rh_CNBu_^+^), respectively. Single crystal X-ray diffraction reveals a N_py_–Rh bond length of 1.969(2) Å and a *δ*(PDI) of 0.161(3) for Rh_CO_^+^, and a N_py_–Rh bond length of 1.942(3) Å and a *δ*(PDI) of 0.152(3) for Rh_CNBu_^+^.

To provide further points of comparison, the solid-state structures of the known rhodium(i) complexes Rh(^Dipp^PDI)Cl (Rh_Cl_), Rh(^Dipp^PDI)Me (Rh_Me_),^34^ and [Rh(^Dipp^PDI)(C_2_H_4_)][BAr^F^_4_] (Rh_ethene_^+^),^37^ were measured by single crystal X-ray diffraction. This allowed their N_py_–Rh bond lengths and *δ*(PDI) values to be determined: N_py_–Rh = 1.898(4) Å, and *δ*(PDI) = 0.108(6) for Rh_Cl_, N_py_–Rh = 1.936(3) Å, and *δ*(PDI) = 0.110(6) for Rh_Me_, N_py_–Rh = 1.960(2) Å, and *δ*(PDI) = 0.152(3) for Rh_ethene_^+^.

**Fig. 7:**
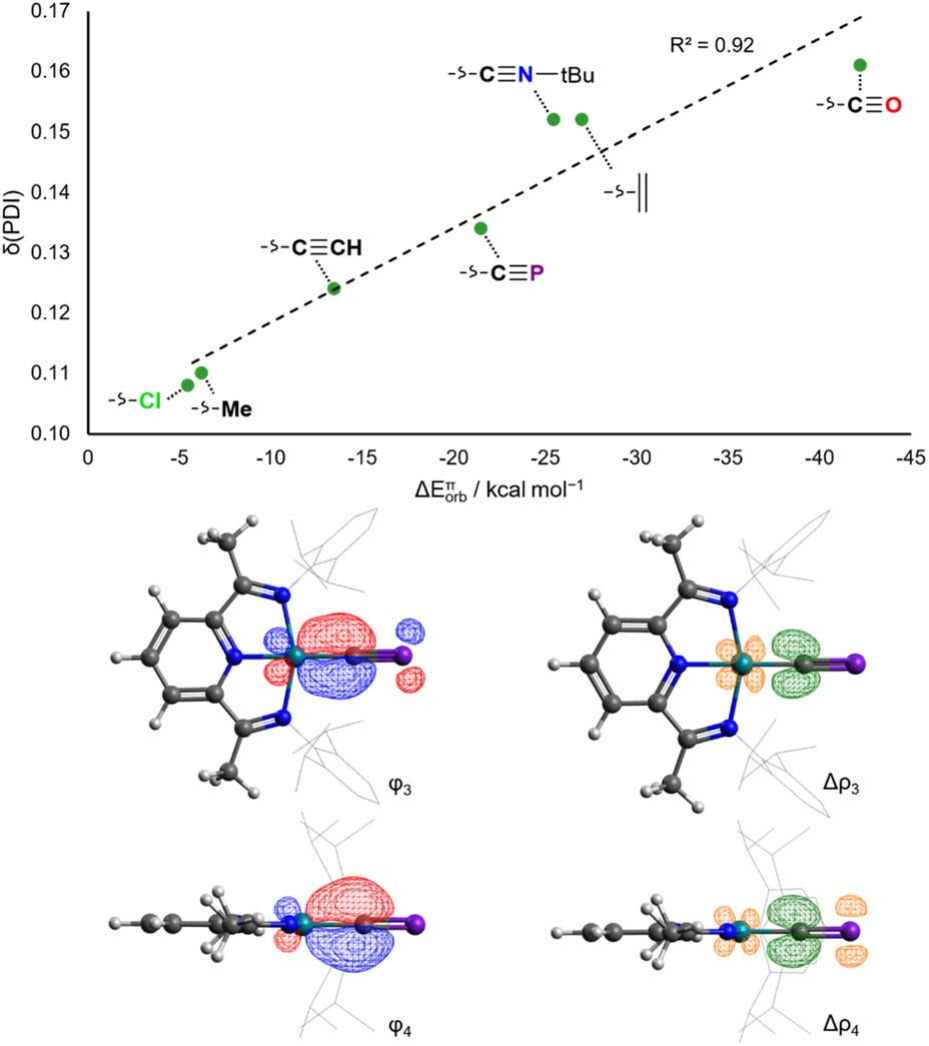
Correlation of calculated ETS-NOCV π backdonation energies with experimental *δ*(PDI) values for Rh_X_ and Rh_L_^+^ (top). Isosurfaces for the π bonding NOCVs and difference densities in Rh_CP_ (bottom).

In contrast to the situation for the analogous cobalt complexes, the *δ*(PDI) values for all the aforementioned Rh_X_ and Rh_L_^+^ complexes vary continuously with different ligands (Fig. 7). Changes to *δ*(PDI) represent different degrees of rhodium(i) to ^Dipp^PDI π backdonation, and consequently are highly sensitive to the π donor/acceptor properties of L. The π donor ligand Cl^−^ in Rh_Cl_ results in a low *δ*(PDI) value (0.108(6)), whereas the strong π acceptor CO results in a high *δ*(PDI) value (0.161(3)) in Rh_CO_^+^. Calculated ETS-NOCV π backdonation energies (Δ*E*^π^_orb_) correlate very well with *δ*(PDI), as do calculated NBO π backdonation energies from 2nd order perturbation theory (*E*^(2)^π). The N_py_–Rh bond length also varies with the *trans*-influence of L, though is also sensitive to σ donating ability, as evidenced by the anomalously high *δ*(PDI) for Rh_Me_ (Fig. S58†).

The rhodium(i) cyaphide complex Rh_CP_ has a *δ*(PDI) of 0.134(3) and N_py_–Rh bond length of 1.943(2) Å. This is likewise reflected in its calculated Δ*E*^π^_orb_ (−21.5 kcal mol^−1^) and *E*^(2)^π (44.1 kcal mol^−1^) values. This places the π accepting ability of the cyaphide ion as being higher than the acetylide ion and almost as high as isocyanides, though still lower than neutral, strong π accepting ligands (*e.g.* CO, C_2_H_4_).

## Conclusions

The cyaphide ion is both a strong σ donor and π acceptor in the terminal, κ*C* coordination mode. The relatively low electronegativity of phosphorus makes the cyaphide ion a slightly stronger σ donor than the cyanide and acetylide ions, and its low energy 2p–3p C≡P π* orbitals make it a potent π acceptor despite its negative charge, with a π acidity comparable to neutral isocyanides. This will undoubtedly have consequences for using the cyaphide ion as a tool to tune the properties of coordination complexes. In particular, its strong π accepting ability in both the η^1^, κ*C* coordination mode as well as the side-on η^2^ coordination mode will make the cyaphide ion especially suitable for use as a bridging ligand in magnetic coordination polymers, for example heavy analogues of Prussian blue.

## Data availability

Crystallographic data has been deposited with the Cambridge Structural Database.

## Author contributions

Conceptualization: J. M. G.; experimental work: E. S. Y. and E. C.; X-ray crystallography: E. S. Y and J. M. G.; computational work: E. S. Y.; writing – original draft: E. S. Y and J. M. G.; writing & editing: all authors; supervision: E. S. Y. and J. M. G.; funding acquisition: J. M. G.

## Conflicts of interest

There are no conflicts to declare.

## Supplementary Material

SC-014-D3SC01126G-s001

SC-014-D3SC01126G-s002

SC-014-D3SC01126G-s003
