# Supplementary material for: Putting cyaphide in its place: determining the donor/acceptor properties of the κC-cyaphido ligand
Source: Chem Sci. 2023 Apr 6;14(17):4627–32. doi: 10.1039/d3sc01126g (PMC10155923; doi:10.1039/d3sc01126g)
Supplement: SC-014-D3SC01126G-s002 [file SC-014-D3SC01126G-s002.pdf]

Electronic Supplementary Material (ESI) for Chemical Science.
This journal is © The Royal Society of Chemistry 2023

Supporting Information

Putting Cyaphide in its Place: Determining the Donor/Acceptor Properties of the κ*C*-Cyaphido Ligand

Contents

1. Experimental section ... 2
   1.1 General experimental methods ... 2
   1.2 Synthesis of reported compounds ... 3
      1.2.1 Synthesis of Co(^{Dipp}PDI)(CCH) (**Co$_{CCH}$**) ... 3
      1.2.2 Synthesis of Na(THF)Co(PIEA)(CCH) ... 5
      1.2.3 Synthesis of [Co(^{Dipp}PDI)(CO)][BAr$^F{}_4$] (**Co$_{CO}$$^+$**) ... 7
      1.2.4 Synthesis of [Co(^{Dipp}PDI)(CN^{t}Bu)][BAr$^F{}_4$] (**Co$_{CNBu}$$^+$**) ... 9
      1.2.5 Synthesis of [Co(^{Dipp}PDI)(CN^{t}Bu)$_2$][BAr$^F{}_4$] ... 11
      1.2.6 Synthesis of Rh(^{Dipp}PDI)(CP) (**Rh$_{CP}$**) ... 13
      1.2.7 Synthesis of Rh(^{Dipp}PDI)(CCH) (**Rh$_{CCH}$**) ... 16
      1.2.8 Synthesis of [Rh(^{Dipp}PDI)(CO)][BAr$^F{}_4$] (**Rh$_{CO}$$^+$**) ... 18
      1.2.9 Synthesis of [Rh(^{Dipp}PDI)(CN^{t}Bu)][BAr$^F{}_4$] (**Rh$_{CNBu}$$^+$**) ... 20
      1.2.10 Synthesis of [Rh(^{Dipp}PDI)(C_2H_4)][BAr$^F{}_4$] (**Rh$_{ethene}$$^+$**) ... 22
      1.2.11 Attempted syntheses of Co(^{Dipp}PDI)(CN) (**Co$_{CN}$**) and Rh(^{Dipp}PDI)(CN) (**Rh$_{CN}$**) ... 24
   1.3 Other characterization data ... 25
2. Single crystal X-ray diffraction data ... 27
3. Computational details ... 43
   3.1. General computational methods ... 43
   3.2. Calculations on anions ... 44
   3.3. Natural Bond Orbital analysis ... 46
   3.4. Energy decomposition analysis ... 47
   3.5. Data analysis ... 50
4. References ... 60

1. Experimental section

1.1 General experimental methods

Synthetic methods. All reactions and product manipulations were carried out using standard Schlenk-line techniques under an inert atmosphere of argon, or in a dinitrogen filled glovebox (MBraun LABstar glovebox maintained at < 0.1 ppm H_2O and < 0.1 ppm O_2). $Na(BAr^F{}_4)$,[1] $Co(^{Dipp}PDI)Cl$,[2] $Co(^{Dipp}PDI)Me$, $[Co(^{Dipp}PDI)(N_2)][B(Me)(C_6F_5)_3]$,[3] $Rh(^{Dipp}PDI)Cl$,[4] and $Rh(^{Dipp}PDI)Me$[5] were synthesized according to previously reported synthetic procedures. Crystals of $Rh(^{Dipp}PDI)Cl$ suitable for SCXRD were obtained from THF/pentane at −20 ºC. Crystals of $Rh(^{Dipp}PDI)Me$ suitable for SCXRD were obtained from toluene/pentane at −35 ºC. $Mg(^{Dipp}NacNac)$(dioxane)(CP) was generated *in situ* according to a previously reported procedure.[6] Ethynylmagnesium chloride (Sigma Aldrich, 0.5 M in THF), sodium acetylide (Sigma Aldrich 95%), *tert*-butyl isocyanide (Sigma Aldrich 98%), carbon monoxide (BOC), ethylene (BOC), potassium cyanide (TCI >98%), trimethylsilyl cyanide (Sigma Aldrich 98%), and anhydrous methanol (99.8%) were purchased and used as received. Tetrabutylammonium cyanide (Sigma Aldrich 95%) was recrystallized from anhydrous THF/hexane. Toluene (Sigma Aldrich HPLC grade), hexane (Sigma Aldrich HPLC grade), and pentane (Sigma Aldrich HPLC grade) were purified using an MBraun SPS-800 solvent system. THF (Sigma Aldrich HPLC grade) was distilled over sodium/benzophenone. 1,2-DFB (Alfa Aesar, 98%) was distilled over CaH_2. C_6D_6 (Aldrich, 99.5%) was degassed and dried over CaH_2. d_8-THF (Aldrich, 99.5%) was distilled over NaK. All dry solvents were stored under argon in gas-tight ampoules over activated 3 Å molecular sieves.

Characterization techniques. NMR spectra were acquired on a Bruker AVIII 400 MHz NMR spectrometer (^{1}H 400 MHz, ^{31}P 162 MHz) or a Bruker Avance NEO 600 MHz NMR spectrometer with a broadband helium cryoprobe (^{13}C 151 MHz). ^{1}H and ^{13}C NMR spectra in deuterated solvents were referenced to the most downfield solvent resonance (^{1}H NMR C_6D_6: δ = 7.16 ppm, d_8-THF: δ = 3.58 ppm; ^{13}C NMR C_6D_6: δ = 128.06 ppm, d_8-THF: δ = 67.21 ppm). ^{1}H and ^{13}C NMR spectra in 1,2-DFB were measured locked to a C_6D_6 capillary and referenced according to literature methods. ^{31}P NMR spectra were externally referenced to an 85% solution of H_3PO_4 in H_2O. Infrared spectra were acquired on a Thermo Scientific iS5 FTIR spectrometer with an iD3 ATR stage. Raman spectra were acquired on a Thermo Fisher Scientific DXR3 SmartRaman spectrometer using a 532 nm laser. UV-vis absorption spectra were acquired for dilute toluene solutions on a Horiba Duetta spectrofluorometer at 298 K using an anaerobic quartz cuvette. Elemental analyses were carried out by Elemental Microanalyses Ltd. (Devon, U.K.) or by London Metropolitan University (London, U.K.). Samples (approx. 5 mg) were submitted in flame sealed glass tubes.

1.2 Synthesis of reported compounds

1.2.1 Synthesis of Co(^{Dipp}PDI)(CCH) (Co$_{CCH}$)

Co(^{Dipp}PDI)Cl (100 mg, 0.17 mmol) was dissolved in THF (10 mL). Ethynylmagnesium chloride (0.7 mL, 0.25 M in THF, 0.18 mmol) was added dropwise, and stirred for 3 days. This was then filtered, and the solvent removed under reduced pressure. The product was crystallized from a 3:1 mixture of pentane/toluene at −20 ºC for 3 days, then isolated by filtration to give Co(^{Dipp}PDI)(CCH) as dark purple crystals. Yield: 33 mg, 0.06 mmol, 34%. Anal. Calcd. (%) for $C_{35}H_{44}N_3Co$: C, 74.31; H, 7.84; N, 7.43. Found: C, 72.86; H, 7.17; N, 6.75.

^{1}H NMR (600 MHz, C_6D_6): δ(ppm) 10.05 (t, $^3J_{H–H}$ = 7.7, 1H, pyridine *para* C**H**), 7.50 (t, $^3J_{H–H}$ = 7.8, 2H, Dipp *para* C**H**), 7.37 (d, $^3J_{H–H}$ = 7.8, 4H, Dipp *meta* C**H**), 7.08 (d, $^3J_{H–H}$ = 7.7, 2H, pyridine *meta* C**H**), 3.27 (sept, $^3J_{H–H}$ = 6.8 Hz, 4H, Dipp C**H**(CH$_3$)$_2$), 2.27 (s, 1H, CC**H**), 1.25 (d, $^3J_{H–H}$ = 6.8 Hz, 12H, Dipp CH(C**H**$_3$)$_2$), 0.94 (d, $^3J_{H–H}$ = 6.8 Hz, 12H, Dipp CH(C**H**$_3$)$_2$), −0.33 (s, 6H, imine NCC**H**$_3$).

^{13}C{^{1}H} NMR (151 MHz, C_6D_6): δ(ppm) 169.05 (s, imine N**C**CH$_3$), 154.83 (s, Dipp *ipso* **C**), 154.25 (s, Dipp *ortho* **C**), 140.84 (s, pyridine *ortho* **C**), 132.24 (s, C**C**H), 126.65 (s, Dipp *para* **C**), 124.42 (s, pyridine *meta* **C**), 123.72 (s, Dipp *meta* **C**), 117.57 (s, pyridine *para* **C**), 29.01 (s, Dipp **C**H(CH$_3$)$_2$), 24.04 (s, Dipp CH(**C**H$_3$)$_2$), 23.53 (s, Dipp CH(**C**H$_3$)$_2$), 22.54 (s, imine NC**C**H$_3$).

UV-vis: λ_{max}(nm) 559.

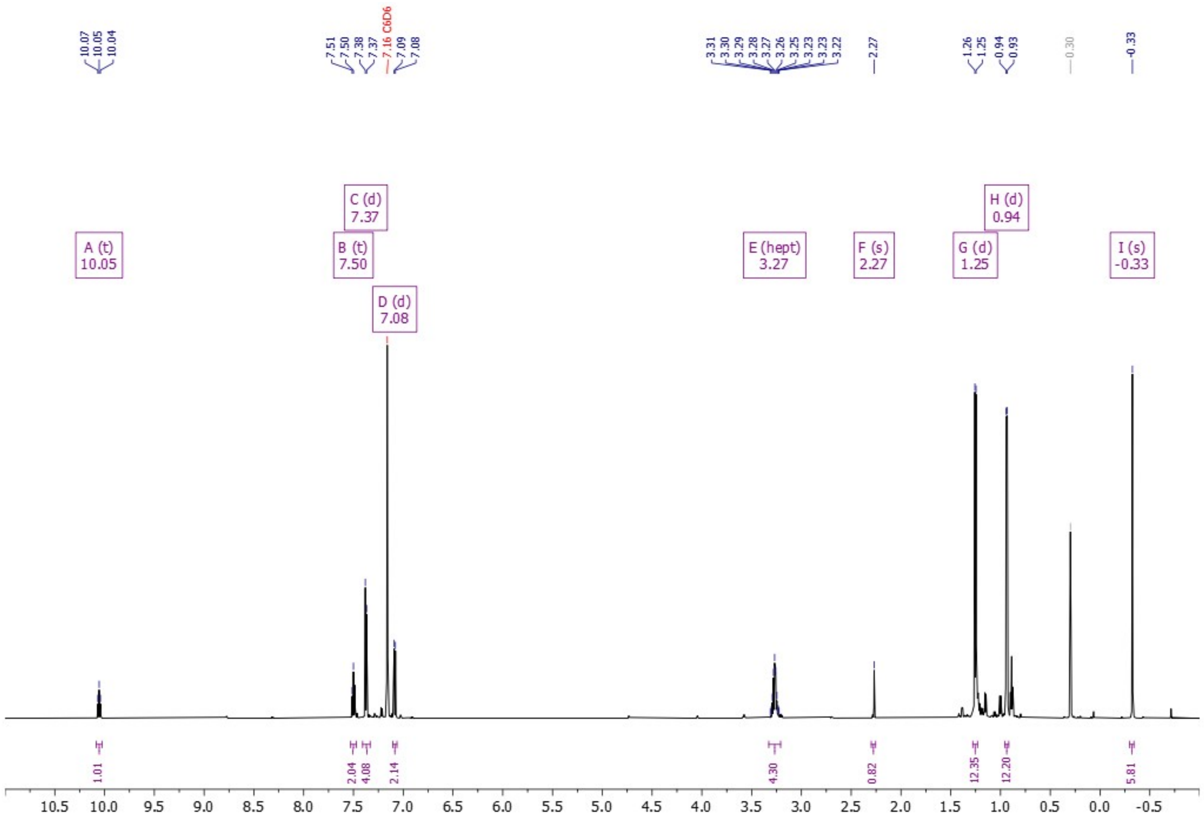

Figure S1. ^{1}H NMR (600 MHz) spectrum of **Co$_{CCH}$** in C_6D_6.

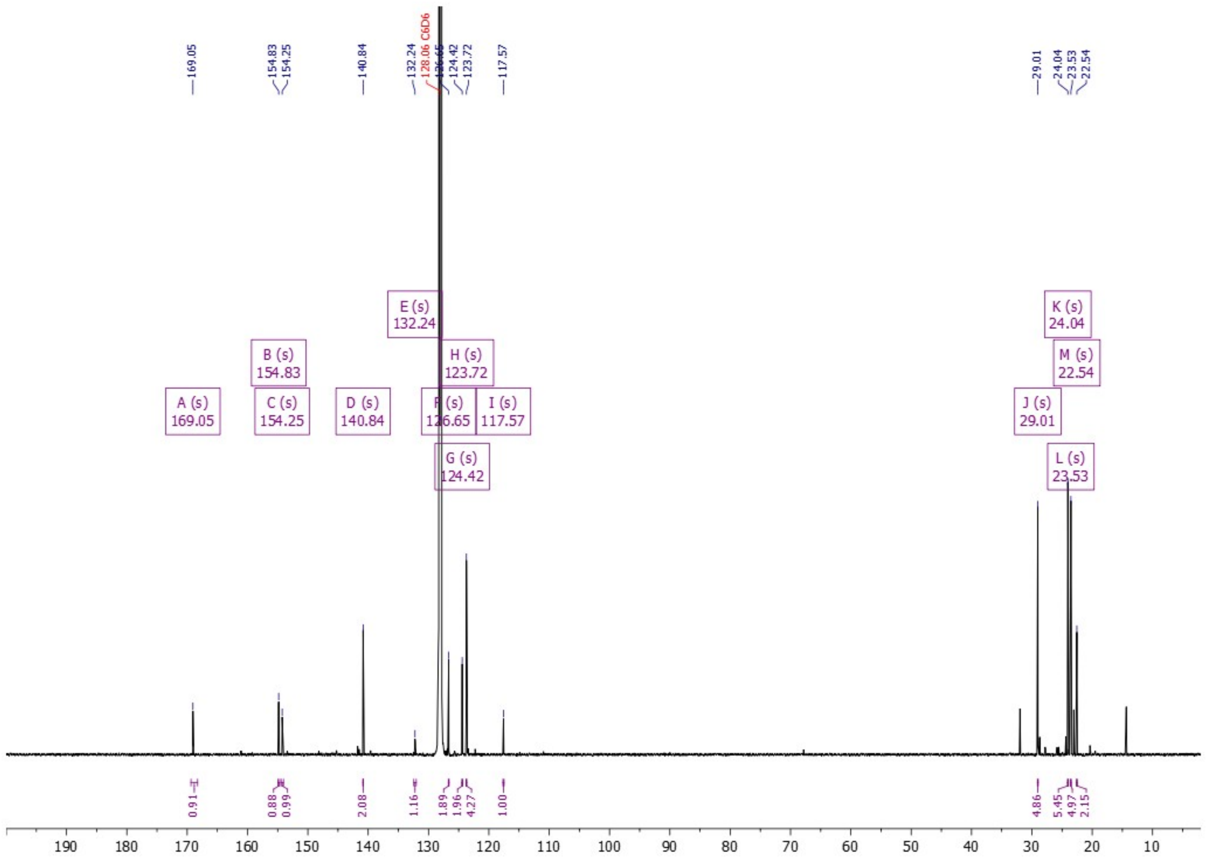

Figure S2. $^{13}C\{^{1}H\}$ NMR (600 MHz) spectrum of $\mathbf{Co_{CCH}}$ in C_6D_6.

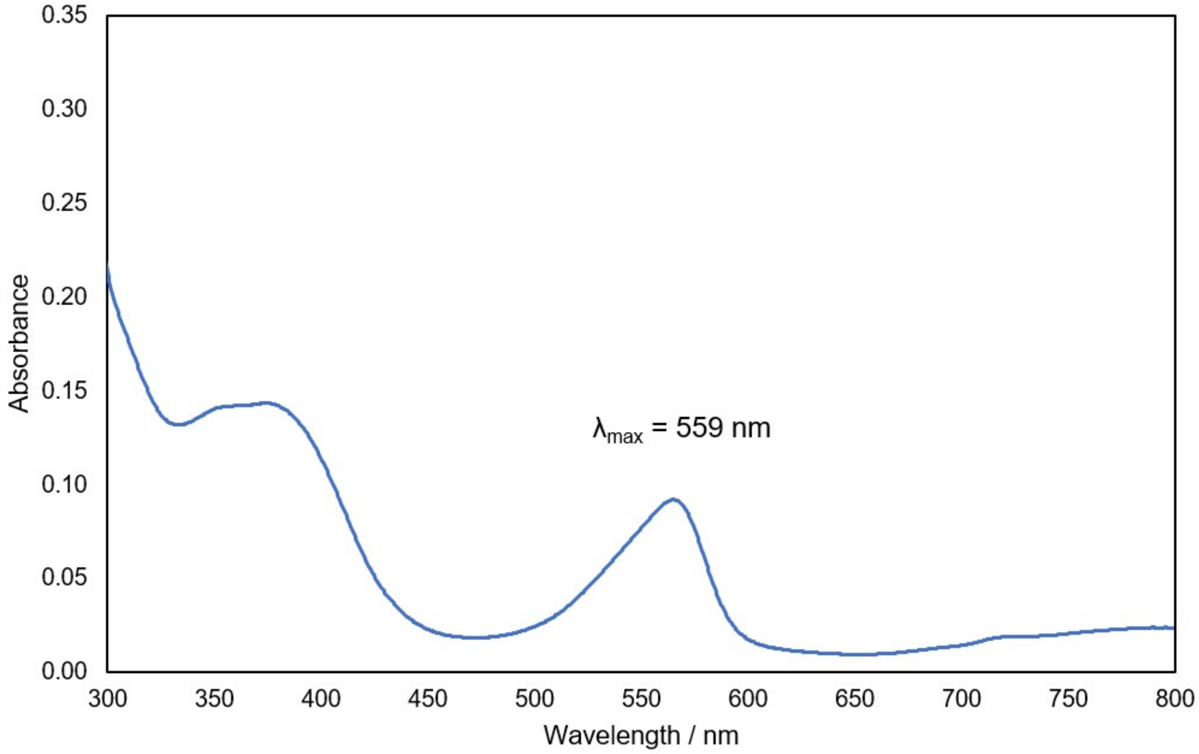

Figure S3. UV-vis absorption spectrum of $\mathbf{Co_{CCH}}$ in toluene.

1.2.2 Synthesis of Na(THF)Co(PIEA)(CCH)

Co(^{Dipp}PDI)Cl (120 mg, 0.21 mmol) and sodium acetylide (20 mg, 0.42 mmol) were dissolved in THF (15 mL), then stirred overnight. The solvent was removed under reduced pressure, then extracted with hexane (3 × 10 mL). This solution was concentrated, then crystallized at −20 ºC for 3 days, yielding dark red crystals of Na(THF)Co(PIEA)(CCH) that were isolated by filtration. Yield: 28 mg, 0.04 mmol, 20%. Anal. Calcd. (%) for $C_{39}H_{51}N_3CoNaO$: C, 71.00; H, 7.79; N, 6.37. Found: C, 69.99; H, 7.42; N, 6.24. In solution, Na(THF)Co(^{Dipp}PIEA)(CCH) (**1**) appears to be in equilibrium with the separated ion pair [Na(THF)$_n$][Co(^{Dipp}PIEA)(CCH)] (**2**).

^{1}H NMR (400 MHz, d$_8$-THF): δ(ppm) 11.00 (d, $^3J_{H–H}$ = 7.3 Hz, 1H, **1** *o*-enamine pyridine *meta* C**H**), 10.51 (t, $^3J_{H–H}$ = 7.8 Hz, 1H, **2** pyridine *para* C**H**), 8.35 (d, $^3J_{H–H}$ = 8.1 Hz, 1H, **1** *o*-imine pyridine *meta* C**H**), 8.22–8.15 (m, 1H, **1** pyridine *para* C**H**), 7.75 (t, $^3J_{H–H}$ = 7.8 Hz, 2H, **2** pyridine *meta* C**H**), 7.43–7.37 (m, 1H, **1** enamine Dipp *para* C**H**), 7.34 (t, $^3J_{H–H}$ = 7.6 Hz, 2H, **2** Dipp *para* C**H**), 7.27 (d, $^3J_{H–H}$ = 7.7 Hz, 2H, **1** enamine Dipp *meta* C**H**), 7.07 (d, $^3J_{H–H}$ = 7.6 Hz, 2H, **2** Dipp *meta* C**H**), 6.78 (dd, $^3J_{H–H}$ = 8.3, 6.6 Hz, 1H, **1** imine Dipp *para* C**H**), 6.72–6.67 (m, 2H, **1** imine Dipp *meta* C**H**), 5.25 (s, 1H, **1** enamine NCC**H**$_2$), 3.47 (s, 1H, **1** enamine NCC**H**$_2$), 3.11 (sept, $^3J_{H–H}$ = 6.9 Hz, 2H, **1** enamine Dipp C**H**(CH$_3$)$_2$), 2.76 (s, 1H, **1** CC**H**), 2.40 (sept, $^3J_{H–H}$ = 6.9 Hz, 2H, **1** imine Dipp C**H**(CH$_3$)$_2$), 2.27 (s, 1H, **2** CC**H**), 2.03 (sept, $^3J_{H–H}$ = 6.9 Hz, 4H, **2** Dipp C**H**(CH$_3$)$_2$), 1.54 (d, $^3J_{H–H}$ = 6.8 Hz, 12H, **2** Dipp CH(C**H**$_3$)$_2$), 1.25 (d, $^3J_{H–H}$ = 6.8 Hz, 6H, **1** enamine Dipp CH(C**H**$_3$)$_2$), 0.94 (d, $^3J_{H–H}$ = 6.8 Hz, 6H, **1** imine Dipp CH(C**H**$_3$)$_2$), 0.66 (d, $^3J_{H–H}$ = 7.0 Hz, 12H, **2** Dipp CH(C**H**$_3$)$_2$), 0.64 (d, $^3J_{H–H}$ = 6.8 Hz, 6H, **1** enamine Dipp CH(C**H**$_3$)$_2$), 0.43 (d, $^3J_{H–H}$ = 6.8 Hz, 6H, **1** imine Dipp CH(C**H**$_3$)$_2$), 0.03 (bs, 5H, **2** imine/enamine NCC**H**$_3$/NCC**H**$_2$)), −4.20 (s, 3H, **1** imine NCC**H**$_3$).

^{13}C{^{1}H} NMR (151 MHz, d$_8$-THF): δ(ppm) 174.87 (**1** enamine N**C**CH$_2$), 164.26 (**2** enamine/imine NCC**H**$_3$/NCC**H**$_2$), 158.43 (**2** Dipp *ipso* **C**), 156.34 (**1** imine N**C**CH$_3$), 155.17 (**1** enamine Dipp *ipso* **C**), 152.01 (**1** imine Dipp *ipso* **C**), 146.80 (**1** imine Dipp *ortho* **C**), 141.15 (**1** enamine Dipp *para* **C**), 135.82 (**2** Dipp *ortho* **C**), 132.18 (**1** pyridine *para* **C**), 131.65 (**2** pyridine *ortho* **C**), 126.42 (**1** enamine Dipp *ortho* **C**), 125.08 (**2** pyridine *meta* **C**), 124.39 (**2** Dipp *para* **C**), 123.62 (**1** enamine Dipp *meta* **C**), 122.55 (**1** imine Dipp *meta* **C**). 121.75 (**2** Dipp *meta* **C**), 121.60 (**1** imine Dipp *para* **C**), 114.66 (**1** *o*-imine pyridine *meta* **C**), 102.20 (**1** *o*-enamine pyridine *meta* **C**), 97.72 (**1** C**C**H), 81.63 (**1** enamine NC**C**H$_2$), 68.03 (**2** **C**CH), 29.15 (**1** enamine Dipp **C**H(CH$_3$)$_2$), 27.87 (**2** Dipp **C**H(CH$_3$)$_2$), 26.94 (**1** imine Dipp **C**H(CH$_3$)$_2$), 26.19 (**1** imine NC**C**H$_3$), 24.59 (**1** imine Dipp CH(**C**H$_3$)$_2$), 24.39 (**2** Dipp CH(**C**H$_3$)$_2$), 23.94 (**1** enamine Dipp CH(**C**H$_3$)$_2$), 23.56 (**1** imine Dipp CH(**C**H$_3$)$_2$), 23.43 (**1** enamine Dipp CH(**C**H$_3$)$_2$), 23.24 (**2** Dipp CH(**C**H$_3$)$_2$).

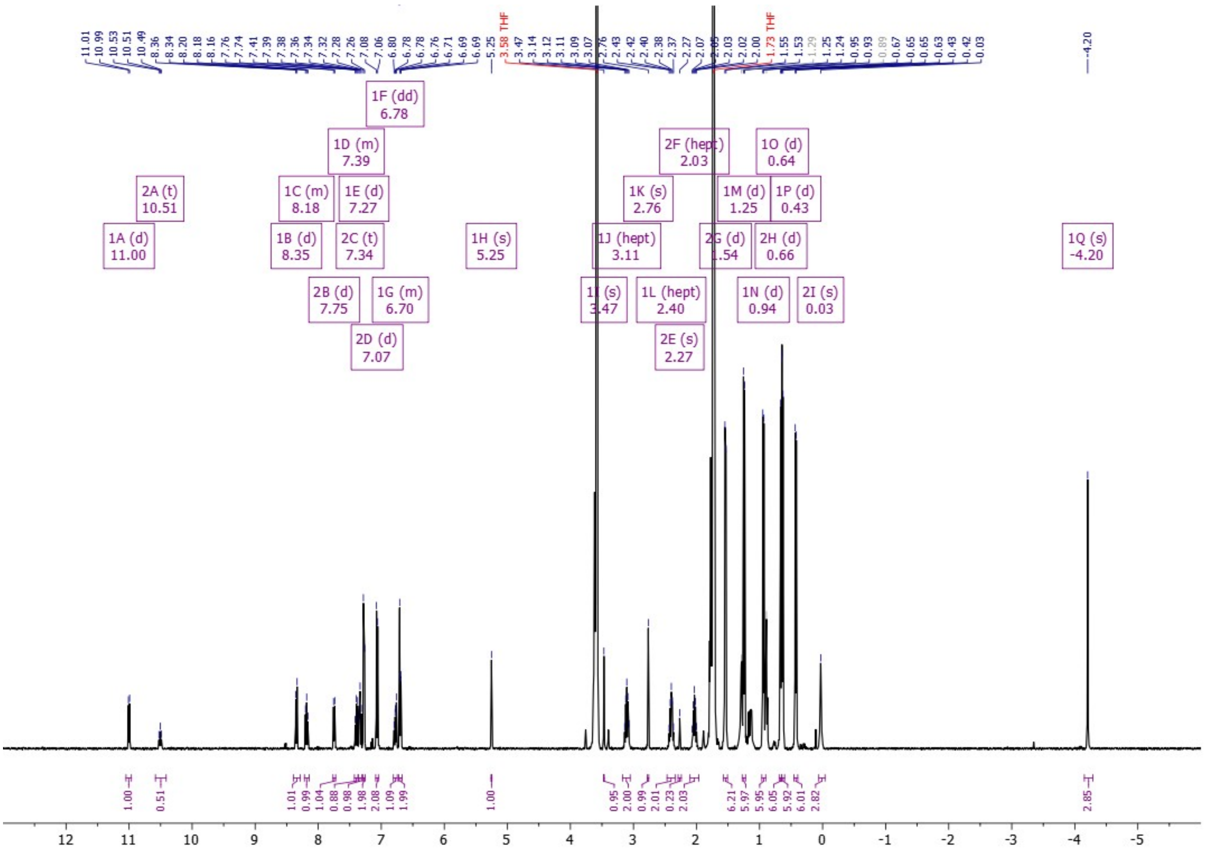

Figure S4. ^{1}H NMR (400 MHz) spectrum of Na(THF)Co(^{Dipp}PIEA)(CCH) in d_8-THF.

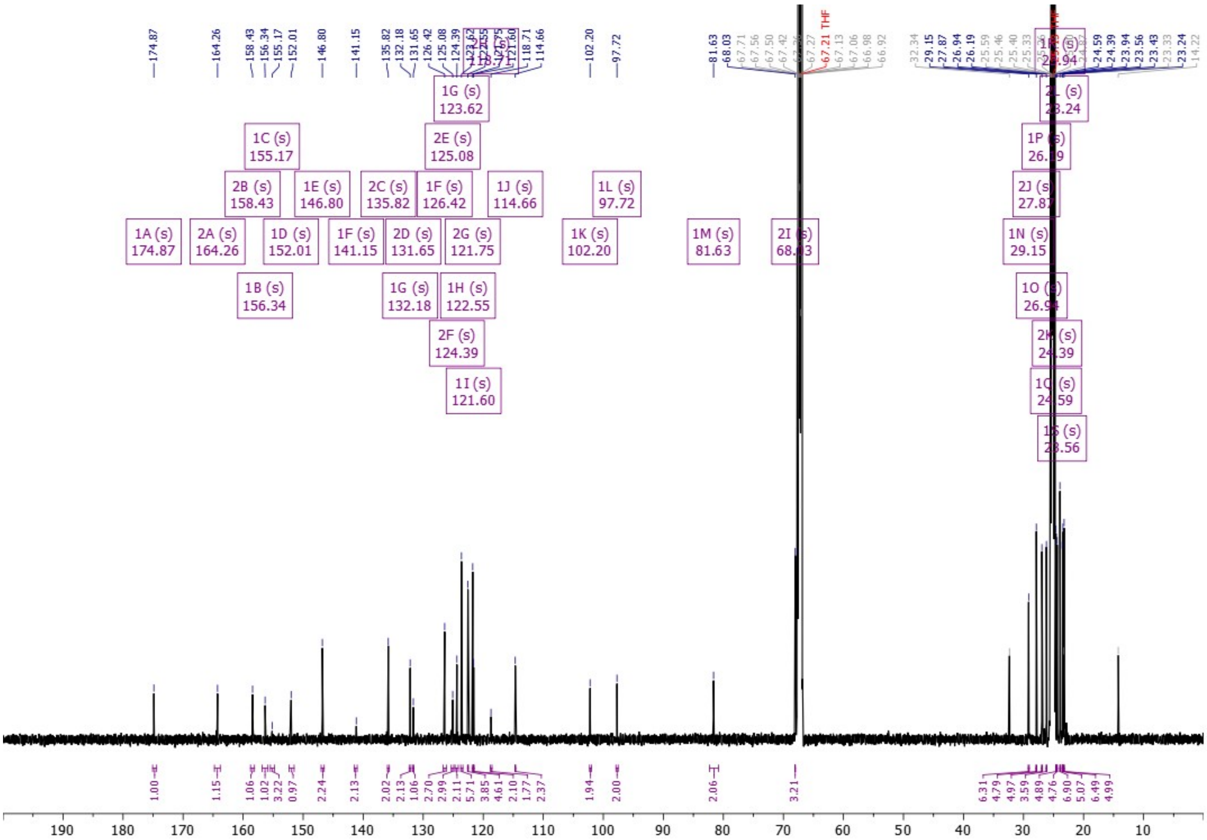

Figure S5. $^{13}C\{^{1}H\}$ NMR (151 MHz) spectrum of Na(THF)Co(^{Dipp}PIEA)(CCH) in d_8-THF.

1.2.3 Synthesis of [Co(^{Dipp}PDI)(CO)][BAr$^F_4$] (Co_{CO}^+)

Co(^{Dipp}PDI)Cl (100 mg, 0.17 mmol) and Na(BAr$^F_4$) (154 mg, 0.17 mmol) were dissolved in 1,2-DFB (3 mL). The solution was degassed, then placed under CO gas (1 bar). The dark blue solution was degassed then filtered. The filtrate was concentrated, then layered with pentane (30 mL) for one week. After filtration, [Co(^{Dipp}PDI)(CO)][BAr$^F_4$] was obtained as large dark blue crystals. Yield: 145 mg, 0.10 mmol, 58%. Anal. Calcd. (%) for $C_{66}H_{55}N_3BCoF_{24}O$: C, 55.36; H, 3.87; N, 2.93. Found: C, 54.92; H, 2.85; N, 2.87.

^{1}H NMR (400 MHz, 1,2-DFB/C_6D_6): δ(ppm) 8.17–8.09 (m, 8H, BAr$^F_4$ *ortho* C**H**) 7.69 (t, $^3J_{H-H}$ = 8.0, 1H, pyridine *para* C**H**), 7.49 (bs, 4H, BAr$^F_4$ *para* C**H**), 7.42 (d, $^3J_{H-H}$ = 8.0, 2H, pyridine *meta* C**H**), 3.01 (sept, $^3J_{H-H}$ = 6.8 Hz, 4H, Dipp C**H**(CH$_3$)$_2$), 1.59 (s, 6H, imine NCC**H**$_3$), 1.30 (d, $^3J_{H-H}$ = 6.8 Hz, 12H, Dipp CH(C**H**$_3$)$_2$), 1.04 (d, $^3J_{H-H}$ = 6.8 Hz, 12H, Dipp CH(C**H**$_3$)$_2$). The signals for some aromatic protons could not be located due to overlap with 1,2-DFB solvent peaks.

^{13}C{^{1}H} NMR (151 MHz, 1,2-DFB/C_6D_6): δ(ppm) 175.64 (s, imine N**C**CH$_3$), 162.60 (q, $^1J_{C-B}$ = 49.7 Hz, BAr$^F_4$ *ipso* C), 157.07 (s, Dipp *ipso* **C**), 144.91 (s, Dipp *ortho* **C**), 141.80 (s, pyridine *para* **C**), 138.29 (s, Ar**C**), 135.16 (bs, BAr$^F_4$ *ortho* **C**), 129.76 (qq, $^2J_{C-F}$ = 31.5 Hz, $^2J_{C-B}$ = 3.0 Hz, BAr$^F_4$ *meta* **C**), 128.86 (s, Ar**C**), 125.86 (s, pyridine *meta* **C**), 124.06 (s, Ar**C**), 117.73–117.56 (m, BAr$^F_4$ *para* **C**), 29.15 (s, Dipp **C**H(CH$_3$)$_2$), 23.01 (s, CH(**C**H$_3$)$_2$), 22.83 (s, Dipp CH(**C**H$_3$)$_2$), 16.48 (s, imine NC**C**H$_3$). The signals for some aromatic carbons could not be assigned due to overlap with 1,2-DFB solvent peaks.

UV-vis: λ_{max}(nm) 593.

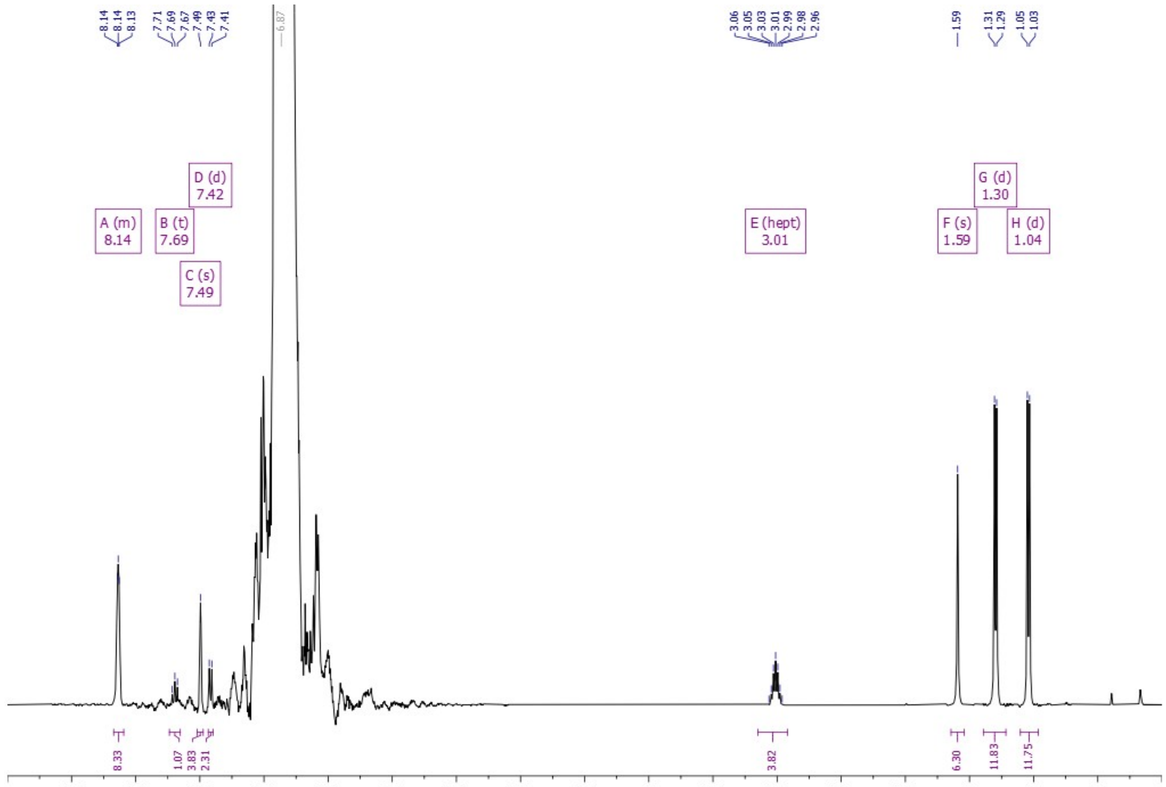

Fi

gure S6. ^{1}H NMR (400 MHz) spectrum of $\mathbf{Co_{CO}}^{+}$ in 1,2-DFB, locked to a C_6D_6 capillary.

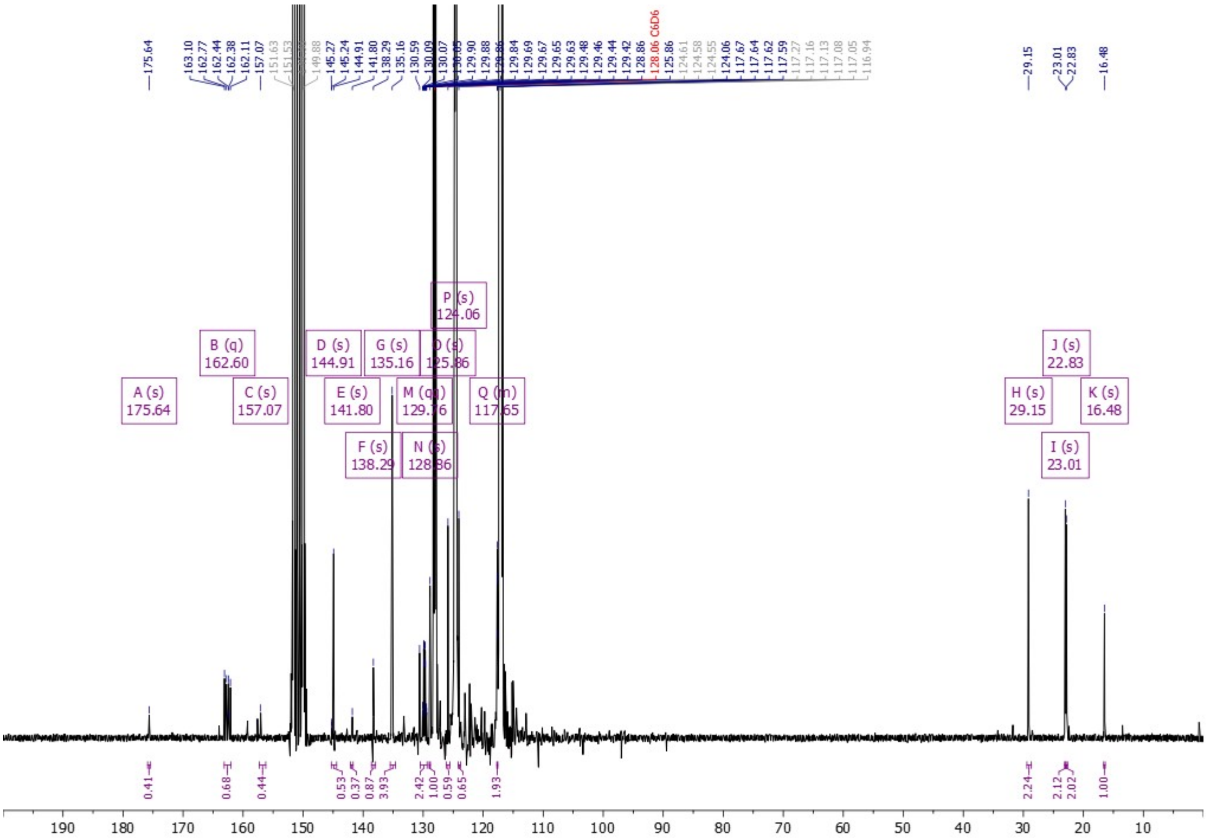

Figure S7. $^{13}C\{^{1}H\}$ NMR (600 MHz) spectrum of $\mathbf{Co_{CO}}^{+}$ in 1,2-DFB, locked to a C_6D_6 capillary.

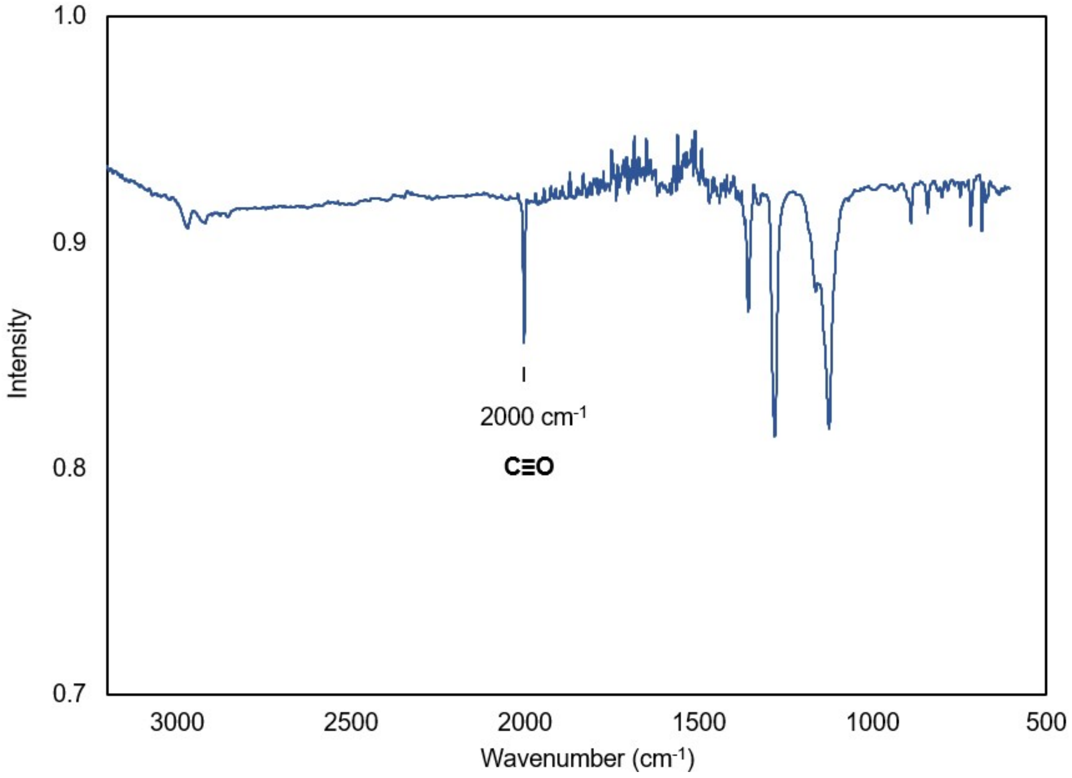

Figure S8. ATR-FTIR spectrum of $\mathbf{Co_{CO}^+}$.

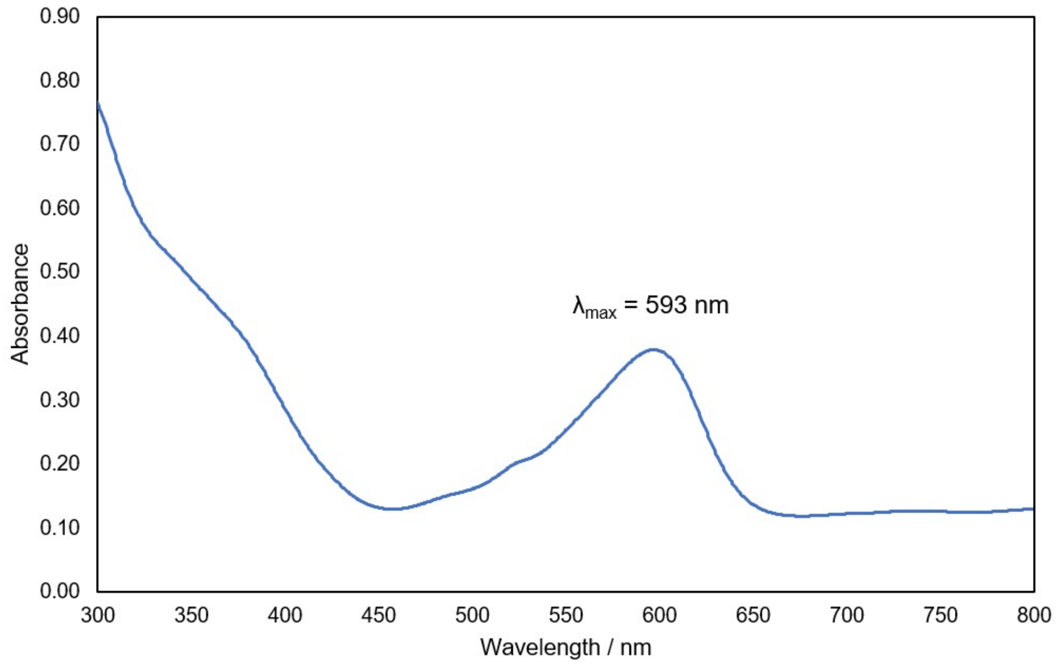

Figure S9. UV-vis absorption spectrum of $\mathbf{Co_{CO}^+}$ in toluene.

1.2.4 Synthesis of [Co(^{Dipp}PDI)(CN^{t}Bu)][BAr$^F_4$] (Co_{CNBu}^+)

Co(^{Dipp}PDI)Cl (50 mg, 0.09 mmol) and Na(BAr$^F_4$) (77 mg, 0.09 mmol) were dissolved in 1,2-DFB (3 mL). A solution of ^{t}BuNC (7 mg, 0.08 mmol) in DFB (0.5 mL) was added dropwise. The dark blue reaction mixture was stirred for 1 h, then filtered. The filtrate was concentrated, then layered with pentane (30 mL) for one week. After filtration, [Co(^{Dipp}PDI)(CN^{t}Bu)][BAr$^F_4$]

was obtained as dark blue crystals. Yield: 99 mg, 0.07 mmol, 77%. Anal. Calcd. (%) for $C_{70}H_{64}N_4BCoF_{24}$: C, 56.54; H, 4.34; N, 3.96. Found: C, 56.75; H, 3.95; N, 4.42.

^{1}H NMR (600 MHz, 1,2-DFB/C_6D_6): δ(ppm) 8.59 (t, $^3J_{H–H}$ = 7.9, 1H, pyridine *para* C**H**), 8.17–8.11 (m, 8H, BAr^F *ortho* C**H**), 7.50 (bs, 4H, BAr^F *para* C**H**), 7.23 (d, $^3J_{H–H}$ = 7.9, 2H, pyridine *meta* C**H**), 2.67 (sept, $^3J_{H–H}$ = 6.9 Hz, 4H, Dipp C**H**(CH_3)$_2$), 1.08 (d, $^3J_{H–H}$ = 6.9 Hz, 12H, Dipp CH(C**H**$_3$)$_2$), 0.96 (s, 6H, imine NCC**H**$_3$), 0.84 (d, $^3J_{H–H}$ = 6.9 Hz, 12H, Dipp CH(C**H**$_3$)$_2$), 0.64 (s, 9H, CNC(C**H**$_3$)$_3$). The signals for some aromatic protons could not be located due to overlap with 1,2-DFB solvent peaks.

^{13}C{^{1}H} NMR (151 MHz, 1,2-DFB/C_6D_6): δ(ppm) 173.05 (s, imine N**C**CH$_3$), 162.58 (q, $^1J_{C–B}$ = 49.7 Hz, BAr$^F{}_4$ *ipso* C), 155.05 (s Dipp *ipso* **C**), 148.99 (s, Dipp *ortho* **C**), 139.85 (s, Ar**C**), 135.13 (bs, BAr$^F{}_4$ *ortho* **C**), 131.46 (s, Ar**C**), 130.57 (s, Ar**C**), 129.74 (qq, $^2J_{C–F}$ = 31.8 Hz, $^2J_{C–B}$ = 2.9 Hz, BAr$^F{}_4$ *meta* **C**), 125.84 (s, Ar**C**), 123.22 (s, Ar**C**), 117.71–117.48 (m, BAr$^F{}_4$ *para* **C**), 58.63 (s, CN**C**(CH$_3$)$_3$), 29.24–28.94 (m, CNC(**C**H$_3$)$_3$ and Dipp **C**H(CH$_3$)$_2$), 23.05 (s, Dipp **C**H(CH$_3$)$_2$), 22.78 (s, Dipp CH(**C**H$_3$)$_2$), 18.05 (s, imine NC**C**H$_3$). The signals for some aromatic carbons were not assigned due to overlap with 1,2-DFB solvent peaks.

UV-vis: λ_{max}(nm) 593.

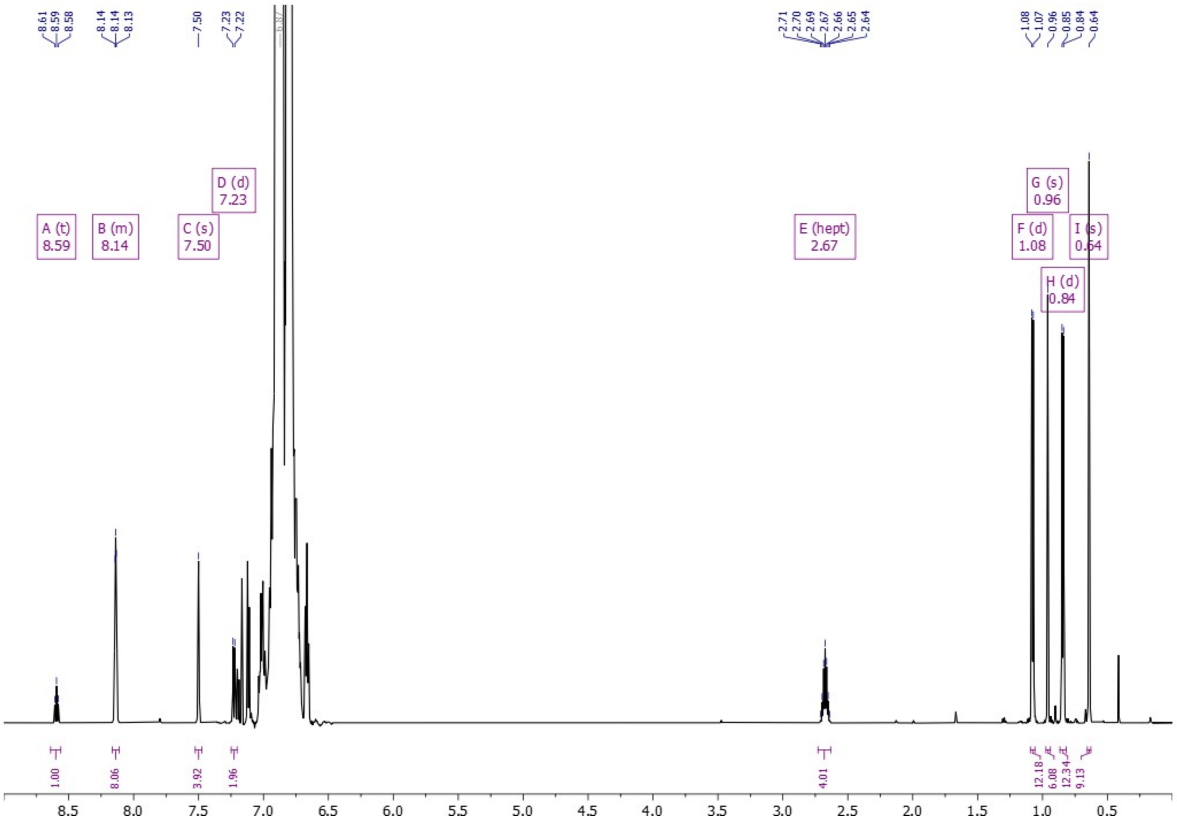

Figure S10. ^{1}H NMR (600 MHz) spectrum of **Co$_{CNBu}$$^+$** in 1,2-DFB, locked to a C_6D_6 capillary.

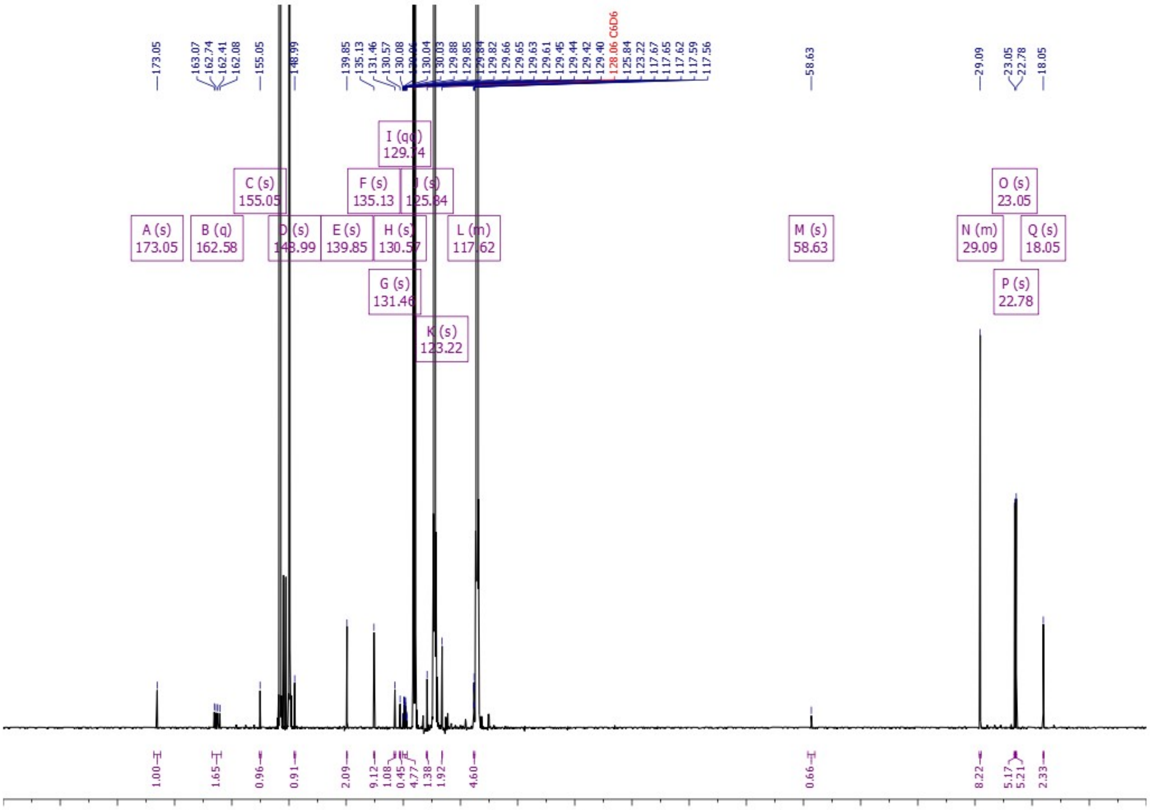

Figure S11. $^{13}C\{^{1}H\}$ NMR (600 MHz) spectrum of **Co_{CNBu}^{+}** in 1,2-DFB, locked to a C_6D_6 capillary.

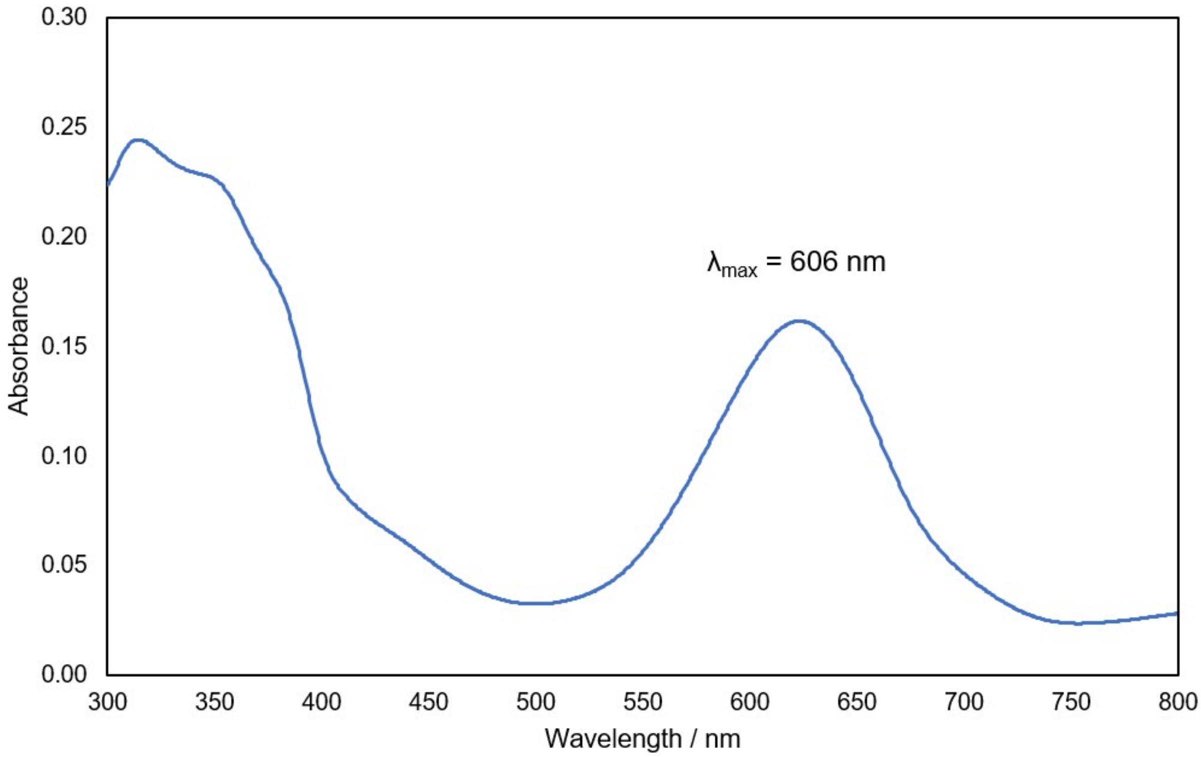

Figure S12. UV-vis absorption spectrum of **Co_{CNBu}^{+}** in toluene.

1.2.5 Synthesis of [Co(^{Dipp}PDI)(CN^{t}Bu)$_2$][BAr$^F_4$]

Co(^{Dipp}PDI)Cl (50 mg, 0.09 mmol) and Na(BAr$^F_4$) (77 mg, 0.09 mmol) were dissolved in 1,2-DFB (3 mL). ^{t}BuNC (17 mg, 0.2 mmol) was added, and the dark blue-green reaction mixture was stirred for 1 h, then filtered. The filtrate was concentrated, then layered with pentane (30 mL) for one week. After filtration, [Co(^{Dipp}PDI)(CN^{t}Bu)$_2$][BAr$^F_4$] was obtained as dark blue-green crystals. Yield: 140 mg, 0.08 mmol, 84%. Anal. Calcd. (%) for $C_{75}H_{73}N_5BCoF_{24}$: C, 57.37; H, 4.69; N, 4.46. Found: C, 57.59; H, 4.91; N, 4.25.

^{1}H NMR (400 MHz, 1,2-DFB/C_6D_6): δ(ppm) 8.17–8.11 (m, 8H, BAr$^F_4$ *ortho* C**H**), 7.84–7.75 (m, 3H, pyridine *para*/*meta* C**H**), 7.50 (bs, 4H, BAr$^F_4$ *para* C**H**), 2.67 (sept, $^3J_{H\text{–}H}$ = 6.7 Hz, 2H, Dipp C**H**(CH$_3$)$_2$), 2.23 (sept, $^3J_{H\text{–}H}$ = 6.9 Hz, 2H, Dipp C**H**(CH$_3$)$_2$), 1.66 (s, 6H, imine NCC**H**$_3$), 1.30 (d, $^3J_{H\text{–}H}$ = 6.7 Hz, 6H, Dipp CH(C**H**$_3$)$_2$), 1.11 (d, $^3J_{H\text{–}H}$ = 6.9 Hz, 6H, Dipp CH(CH$_3$)$_2$), 0.93 (d, $^3J_{H\text{–}H}$ = 6.9 Hz, 6H, Dipp CH(C**H**$_3$)$_2$), 0.90 (s, 9H, CNC(C**H**$_3$)$_3$), 0.80 (d, $^3J_{H\text{–}H}$ = 6.7 Hz, 6H, Dipp CH(C**H**$_3$)$_2$), 0.66 (s, 9H, CNC(C**H**$_3$)$_3$). The signals for some aromatic protons could not be located due to overlap with 1,2-DFB solvent peaks.

^{13}C{^{1}H} NMR (151 MHz, 1,2-DFB/C_6D_6): δ(ppm) 184.29 (s, imine N**C**CH$_3$), 162.58 (q, $^1J_{C\text{–}B}$ = 49.8 Hz, BAr$^F_4$ *ipso* C), 162.37 (s pyridine Ar**C**), 148.99 (s, pyridine *ortho* **C**), 141.10 (s, Dipp Ar**C**), 138.25 (s, Dipp Ar**C**), 135.13 (bs, BAr$^F_4$ *ortho* **C**), 130.57 (s, Dipp Ar**C**), 129.77 (qq, $^2J_{C\text{–}F}$ = 31.3 Hz, $^2J_{C\text{–}B}$ = 3.2 Hz, BAr$^F_4$ *meta* **C**), 127.40 (s, Dipp Ar**C**), 125.84 (s, Ar**C**), 125.20 (s, Ar**C**), 123.48 (s, Ar**C**), 117.73–117.56 (m, BAr$^F_4$ *para* **C**), 57.97 (s, CN**C**(CH$_3$)$_3$), 57.84 (s, CN**C**(CH$_3$)$_3$), 29.16 (s, CNC(**C**H$_3$)$_3$), 29.08 (s, CNC(**C**H$_3$)$_3$), 27.91 (s, Dipp **C**H(CH$_3$)$_2$), 27.83 (s, Dipp **C**H(CH$_3$)$_2$), 25.49 (s, Dipp CH(**C**H$_3$)$_2$), 23.65 (s, Dipp CH(**C**H$_3$)$_2$), 23.64 (s, Dipp CH(**C**H$_3$)$_2$), 22.71 (s, Dipp CH(**C**H$_3$)$_2$), 16.21 (s, imine NC**C**H$_3$). The signals for some aromatic carbons were not assigned due to overlap with 1,2-DFB solvent peaks.

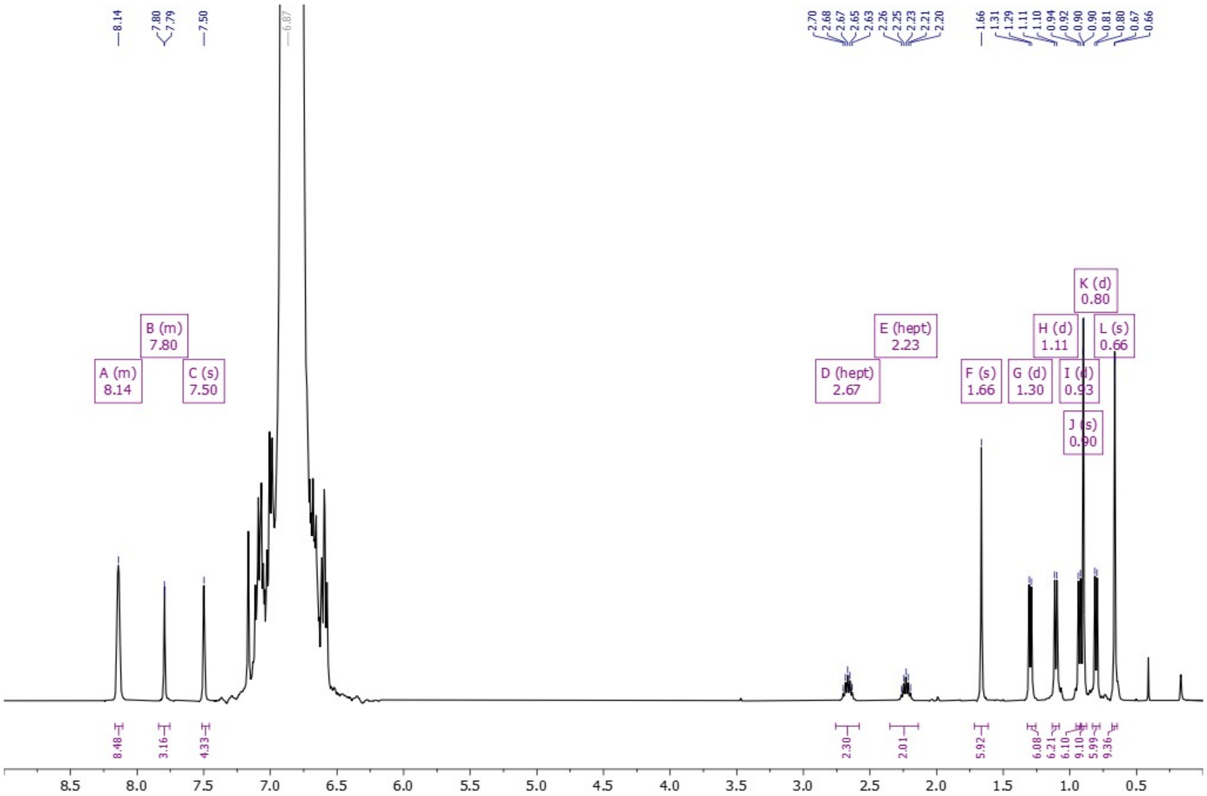

Figure S13. ^{1}H NMR (600 MHz) spectrum of [Co(^{Dipp}PDI)(CN^{t}Bu)$_2$][BAr^{F}$_4$] in 1,2-DFB, locked to a C_6D_6 capillary.

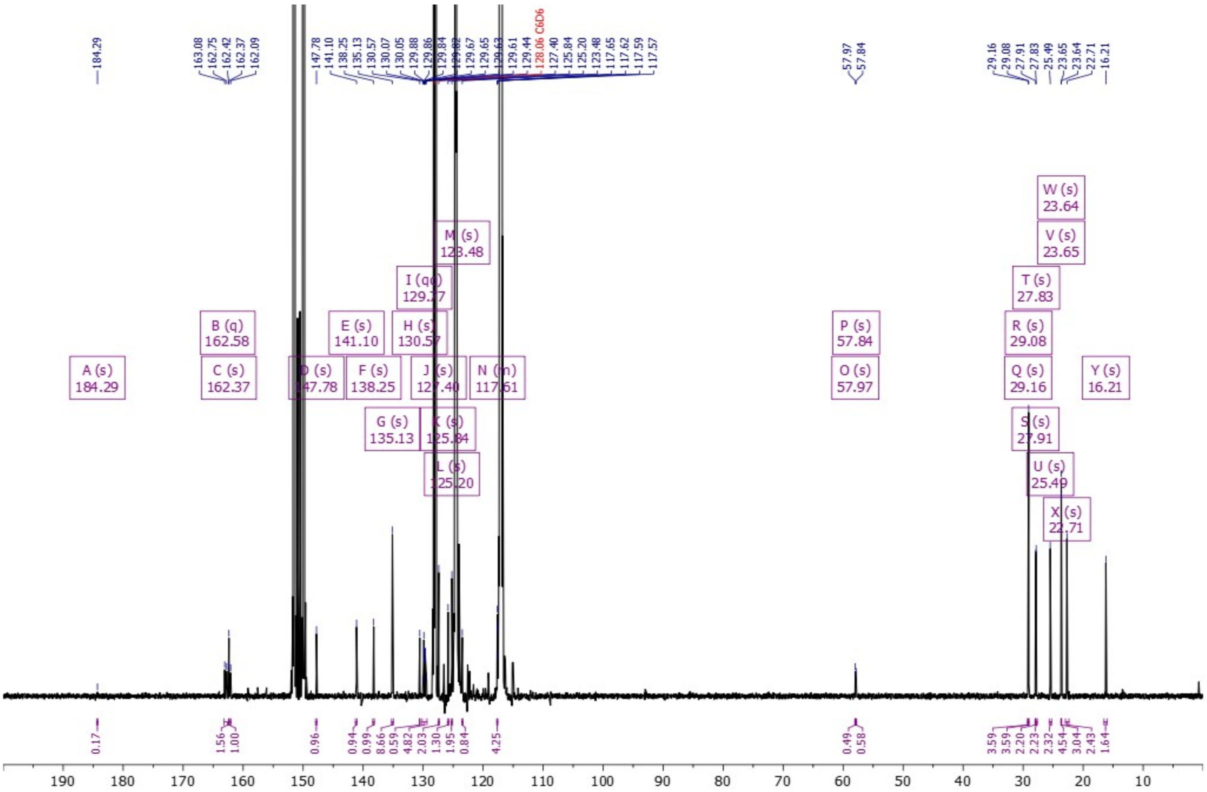

Figure S14. ^{13}C{^{1}H} NMR (600 MHz) spectrum of [Co(^{Dipp}PDI)(CN^{t}Bu)$_2$][BAr^{F}$_4$] in 1,2-DFB, locked to a C_6D_6 capillary.

1.2.6 Synthesis of Rh(^{Dipp}PDI)(CP) (Rh$_{CP}$)

Mg(^{Dipp}Nacnac)(dioxane)(CP) (approx. 65 mg, 0.11 mmol) was generated *in situ* in toluene (1 mL). Rh(^{Dipp}PDI)Cl (62 mg, 0.10 mmol) was added as a solid to the solution of Mg(^{Dipp}Nacnac)(dioxane)(CP), and the mixture stirred at room temperature for 3 days. The mixture was filtered, and the solvent removed from the filtrate *in vacuo*. The residue was washed with hexane (3 × 1 mL), then extracted with toluene (1 mL). The dark green solution was concentrated *in vacuo*, then layered with hexane and stored at −35 °C for 3 days, yielding Rh(^{Dipp}PDI)(CP) as green-black crystals. The crystals were isolated by filtration, and washed with cold hexane (2 × 0.5 mL). Yield: 14 mg, 0.02 mmol, 22%. Anal. Calcd. (%) for $C_{34}H_{43}N_3PRh$: C, 65.07; H, 6.91; N, 6.70. Found: C, 63.69; H, 5.91; N, 5.65.

^{1}H NMR (400 MHz, C_6D_6): δ(ppm) 7.48 (t, $^3J_{H\text{–}H}$ = 7.9, 1H, pyridine *para* C**H**), 7.26–7.22 (m, 6H, Dipp *para/meta* C**H**), 6.85 (d, $^3J_{H\text{–}H}$ = 7.9, 2H, pyridine *meta* C**H**), 3.28 (sept, $^3J_{H\text{–}H}$ = 6.8 Hz, 4H, Dipp C**H**(CH_3)$_2$), 1.53 (d, $^3J_{H\text{–}H}$ = 6.8 Hz, 12H, Dipp CH(C**H**$_3$)$_2$), 1.09 (d, $^3J_{H\text{–}H}$ = 6.8 Hz, 12H, Dipp CH(C**H**$_3$)$_2$), 1.09 (s, 6H, imine NCC**H**$_3$).

^{13}C{^{1}H} NMR (151 MHz, C_6D_6): δ(ppm) 264.15 (dd, $^1J_{C\text{–}Rh}$ = 54.8 Hz, $^1J_{C\text{–}P}$ = 22.9 Hz, Rh**C**P), 166.98 (s, imine N**C**CH$_3$), 156.34 (s, Dipp *ipso* **C**), 146.30 (s, Dipp *ortho* **C**), 140.63 (s, Dipp *para* **C**), 127.01 (s, Dipp *meta* **C**), 124.61 (s, pyridine *meta* **C**), 123.59 (s, pyridine *ortho* **C**), 121.61 (s, pyridine *para* **C**), 28.68 (s, Dipp **C**H(CH_3)$_2$), 24.05 s, Dipp CH(**C**H$_3$)$_2$), 23.88 (s, Dipp CH(**C**H$_3$)$_2$), 17.41 (s, imine NC**C**H$_3$).

^{31}P{^{1}H} NMR (162 MHz, C_6D_6): δ(ppm) 251.4 (d, $^2J_{P\text{–}Rh}$ = 6 Hz,C**P**).

Raman: 1334 cm^{-1} (m, *v*(C≡P)).

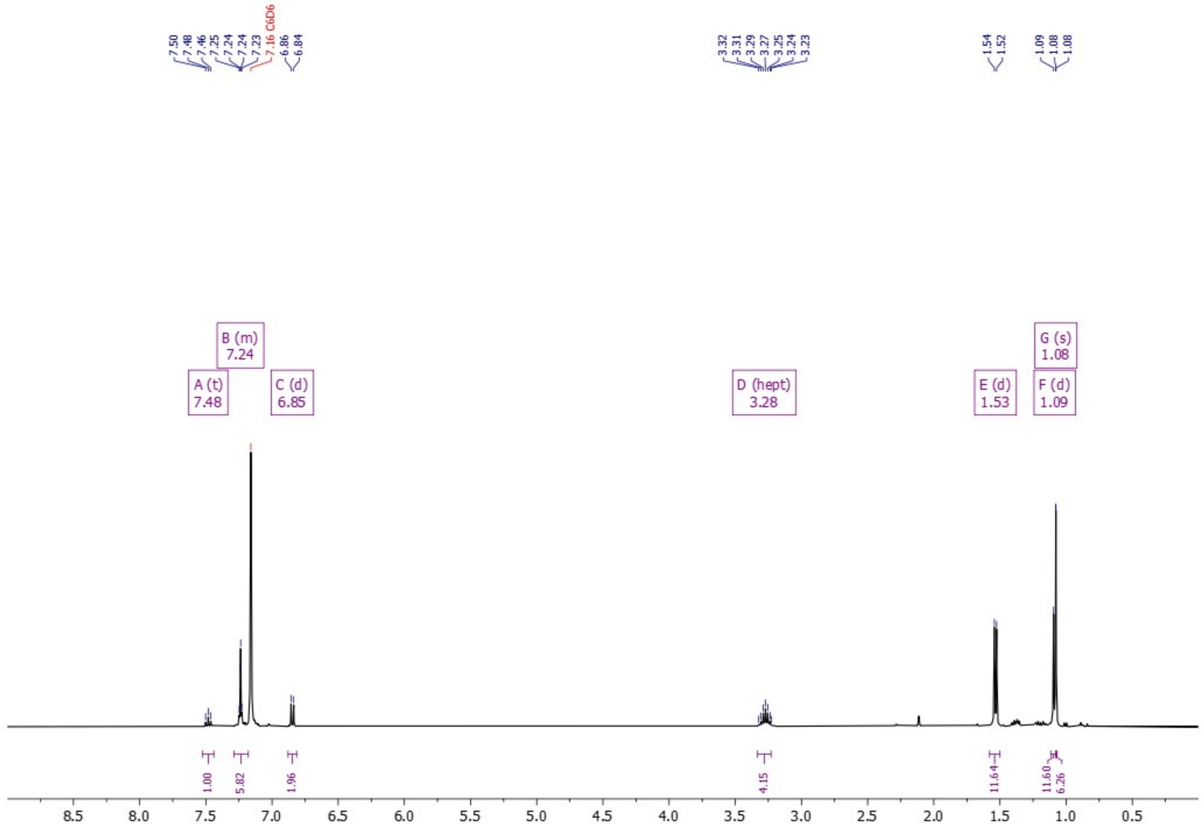

Figure S15. ^{1}H NMR (400 MHz) spectrum of **Rh$_{CP}$** in C_6D_6.

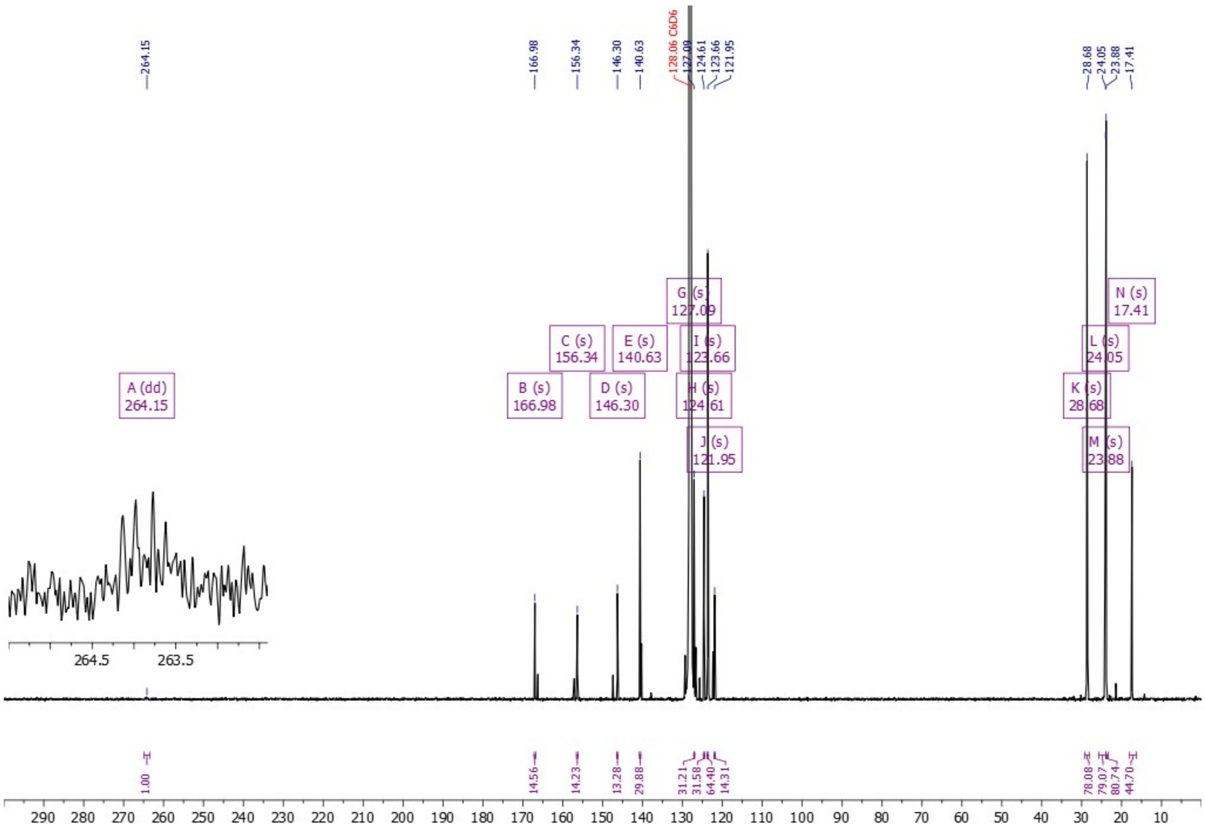

Figure S16. $^{13}C\{^1H\}$ NMR (151 MHz) spectrum of **Rh$_{CP}$** in C_6D_6.

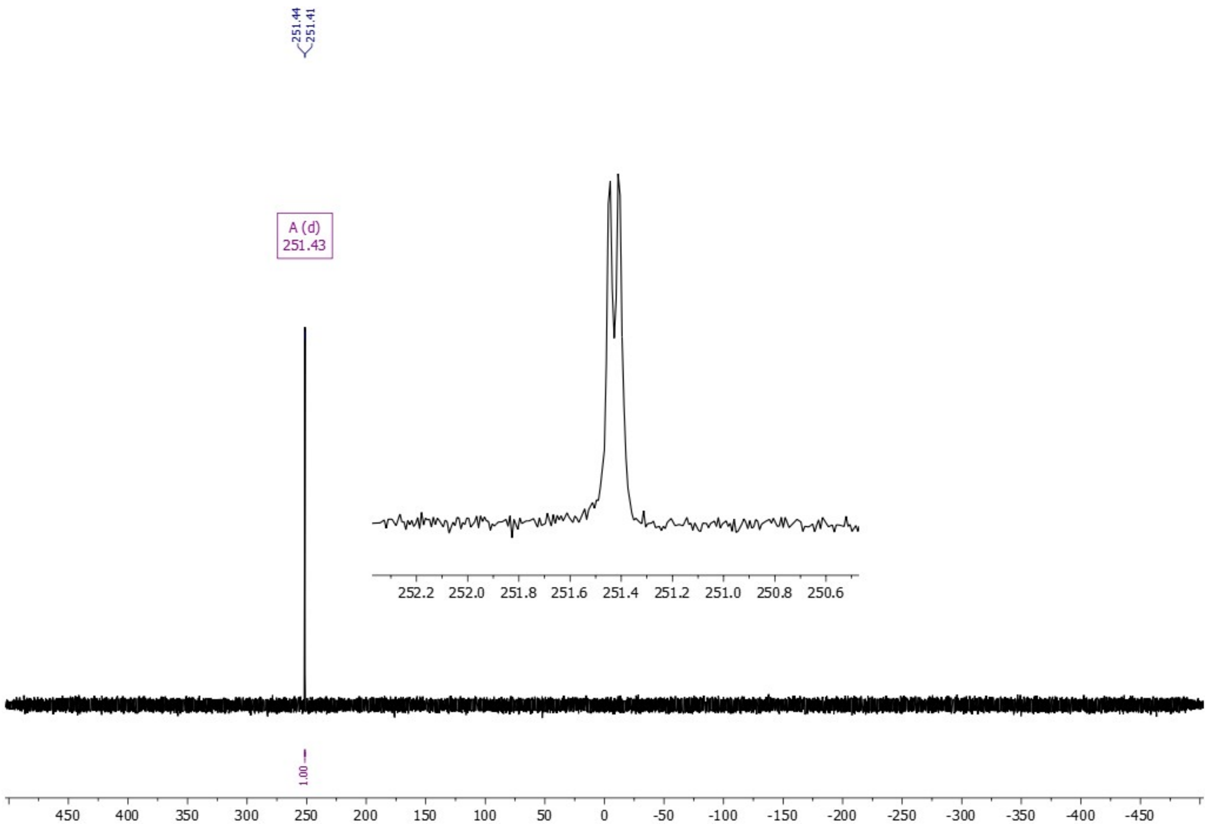

Figure S17. $^{31}P\{^{1}H\}$ NMR (162 MHz) spectrum of $\mathbf{Rh_{CP}}$ in C_6D_6.

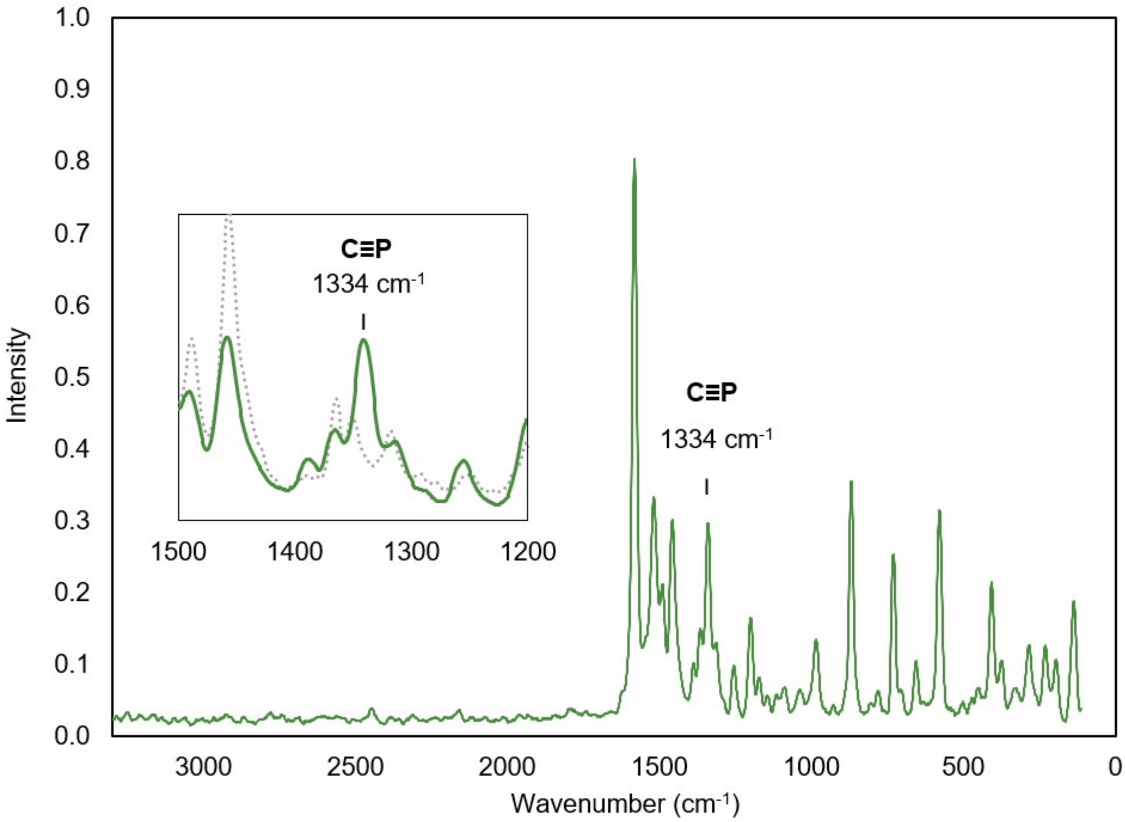

Figure S18. Dispersive Raman spectrum of $\mathbf{Rh_{CP}}$. For comparison, the Raman spectrum of Rh(^{Dipp}PDI)Cl between 1200–1500 cm^{-1} is shown as a dotted line in the inset.

1.2.7 Synthesis of Rh(^{Dipp}PDI)(CCH) (Rh$_{CCH}$)

Rh(^{Dipp}PDI)Cl (100 mg, 0.16 mmol) was dissolved in THF (10 mL). Ethynylmagnesium chloride (0.7 mL, 0.25 M in THF, 0.18 mmol) was added dropwise, and stirred for 3 days. This was then filtered, and the solvent removed under reduced pressure to give Rh(^{Dipp}PDI)(CCH) as a green solid. Yield: 60 mg, 0.10 mmol, 61%. Anal. Calcd. (%) for $C_{35}H_{44}N_3Rh$: C, 68.95; H, 7.27; N, 6.89. Found: C, 66.89; H, 6.37; N, 6.10. Crystals suitable for X-ray diffraction were obtained from toluene/pentane by vapor diffusion.

^{1}H NMR (600 MHz, C_6D_6): δ(ppm) 7.78 (t, $^3J_{H–H}$ = 7.9, 1H, pyridine *para* C**H**), 7.25–7.22 (m, 6H, Dipp *para/meta* C**H**), 6.97 (d, $^3J_{H–H}$ = 7.9, 2H, pyridine *meta* C**H**), 3.27 (sept, $^3J_{H–H}$ = 6.8 Hz, 4H, Dipp C**H**(CH_3)$_2$), 3.03 (d, $^3J_{H–Rh}$ = 1.3 Hz, 1H, CC**H**), 1.42 (d, $^3J_{H–H}$ = 6.8 Hz, 12H, Dipp CH(C**H**$_3$)$_2$), 1.12 (d, $^3J_{H–H}$ = 6.8 Hz, 12H, Dipp CH(C**H**$_3$)$_2$), 1.04 (s, 6H, imine NCC**H**$_3$).

^{13}C{^{1}H} NMR (151 MHz, C_6D_6): δ(ppm) 166.35 (s, imine N**C**CH$_3$), 156.41 (d, $^2J_{C–Rh}$ = 2.8 Hz, Dipp *ipso* **C**), 148.37 (s, Dipp *ortho* **C**), 140.41 (s, Dipp *para* **C**), 126.52 (s, Dipp *meta* **C**), 125.00 (s, pyridine *meta* **C**), 123.50 (s, pyridine *ortho* **C**), 123.49 (d, $^2J_{C–Rh}$ = 11.3 Hz, C**C**H), 122.94 (s, pyridine *para* **C**), 114.28 (d, $^1J_{C–Rh}$ = 60.6 Hz, **C**CH), 28.57 (s, Dipp **C**H(CH$_3$)$_2$), 23.96 (s, Dipp **C**H(CH$_3$)$_2$), 23.81 (s, Dipp CH(**C**H$_3$)$_2$), 17.58 (d, $^3J_{C–Rh}$ = 1.5 Hz imine NC**C**H$_3$).

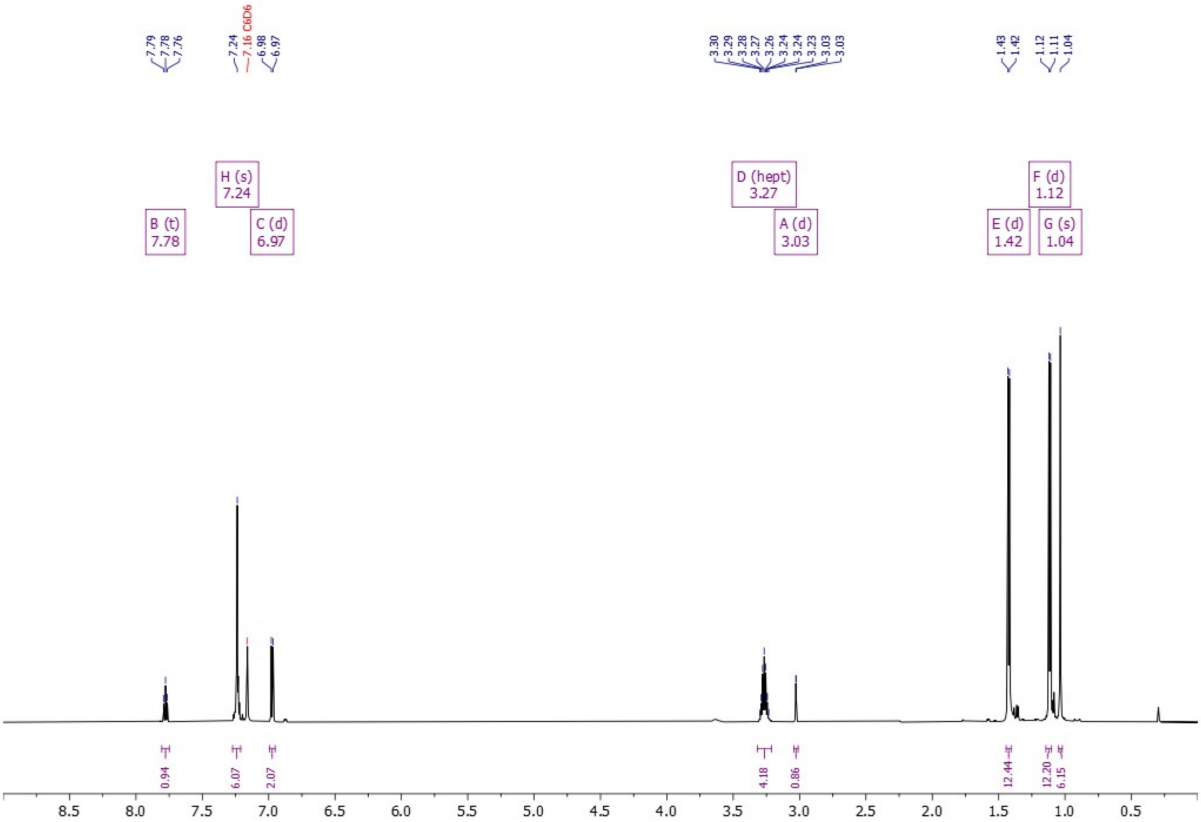

Figure S19. ^{1}H NMR (600 MHz) spectrum of **Rh$_{CCH}$** in C_6D_6.

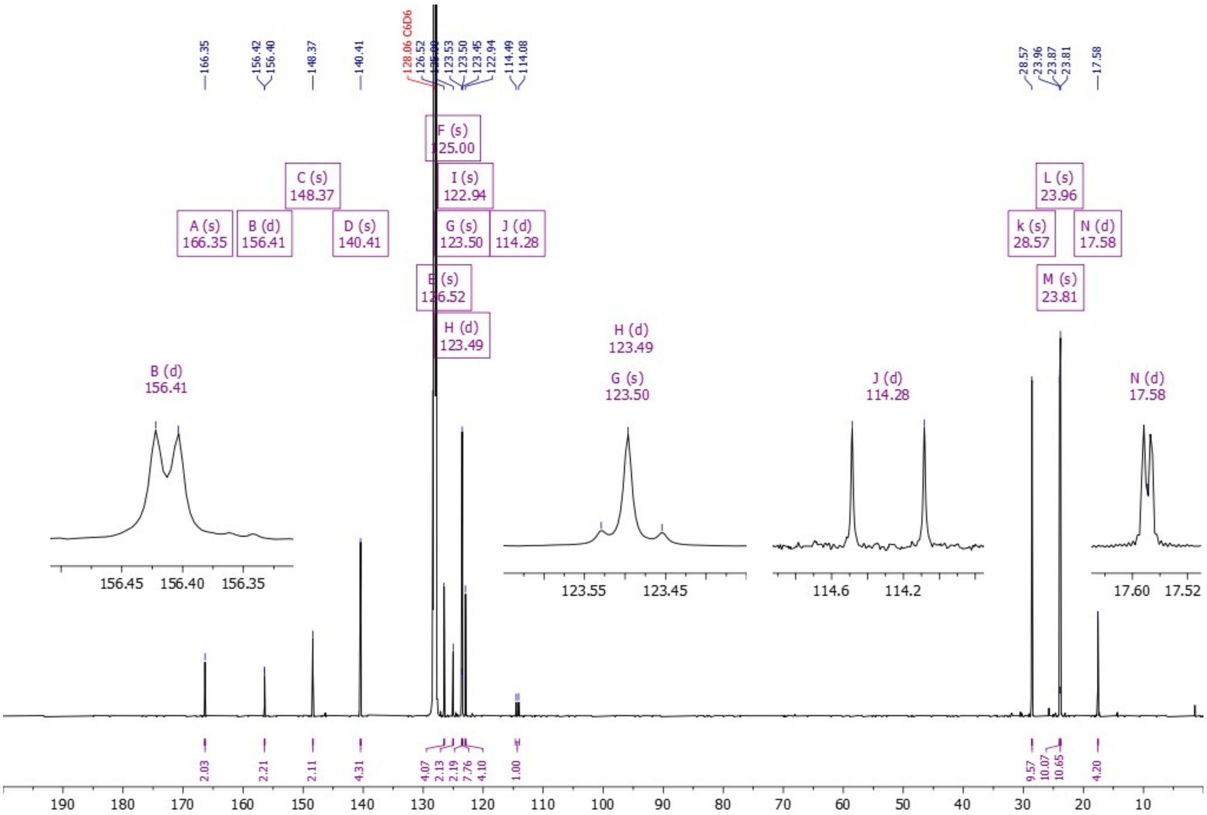

Figure S20. $^{13}C\{^{1}H\}$ NMR (151 MHz) spectrum of $\mathbf{Rh_{CCH}}$ in C_6D_6.

1.2.8 Synthesis of [Rh(^{Dipp}PDI)(CO)][BAr$^F_4$] ($\mathbf{Rh_{CO}^+}$)

Rh(^{Dipp}PDI)Cl (100 mg, 0.16 mmol) and Na(BAr$^F_4$) (144 mg, 0.16 mmol) were dissolved in 1,2-DFB (3 mL). The solution was degassed, then placed under CO gas (1 bar). After stirring for 1 hour, the solution was degassed then filtered. The solution was concentrated, then layered with pentane (30 mL) for one week. After filtration, [Rh(^{Dipp}PDI)(CO)][BAr$^F_4$] was obtained as large dark green crystals. Yield: 181 mg, 0.12 mmol, 76%. Anal. Calcd. (%) for $C_{66}H_{55}N_3BF_{24}ORh$: C, 53.71; H, 3.76; N, 2.85. Found: C, 53.39; H, 3.68; N, 2.74.

^{1}H NMR (600 MHz, 1,2-DFB/C_6D_6): δ(ppm) 8.17–8.09 (m, 8H, BAr$^F_4$ *ortho* C**H**) 7.86 (t, $^3J_{H–H}$ = 8.1, 1H, pyridine *para* C**H**), 7.63 (d, $^3J_{H–H}$ = 8.1, 2H, pyridine *meta* C**H**), 7.49 (bs, 4H, BAr$^F_4$ *para* C**H**), 3.16 (sept, $^3J_{H–H}$ = 6.8 Hz, 4H, Dipp C**H**(CH$_3$)$_2$), 1.97 (s, 6H, imine NCC**H**$_3$), 1.26 (d, $^3J_{H–H}$ = 6.8 Hz, 12H, Dipp CH(C**H**$_3$)$_2$), 1.08 (d, $^3J_{H–H}$ = 6.8 Hz, 12H, Dipp CH(C**H**$_3$)$_2$). The signals for some aromatic protons could not be located due to overlap with 1,2-DFB solvent peaks.

^{13}C{^{1}H} NMR (151 MHz, 1,2-DFB/C_6D_6): δ(ppm) 187.42 (d, $^1J_{C–Rh}$ = 77.2 Hz, **C**O), 176.19 (s, imine N**C**CH$_3$), 162.59 (q, $^1J_{C–B}$ = 49.7 Hz, BAr$^F_4$ *ipso* C), 156.04 (d, $^2J_{C–Rh}$ = 3.1 Hz, Dipp *ipso* **C**), 145.21 (s, Dipp *ortho* **C**), 142.26 (s, pyridine *para* **C**), 137.77 (s, Ar**C**), 135.14 (bs, BAr$^F_4$ *ortho* **C**), 129.75 (qq, $^2J_{C–F}$ = 31.6 Hz, $^2J_{C–B}$ = 2.9 Hz, BAr$^F_4$ *meta* **C**), 128.79 (s, Ar**C**), 125.85 (s, pyridine *meta* **C**), 117.70–117.58 (m, BAr$^F_4$ *para* **C**), 28.71 (s, Dipp **C**H(CH$_3$)$_2$), 22.78 (s, Dipp **C**H(CH$_3$)$_2$), 22.72 (s, Dipp CH(**C**H$_3$)$_2$), 16.44 (d, $^3J_{C–Rh}$ = 1.8 Hz, imine NC**C**H$_3$). The

signals for some aromatic carbons could not be located or were not assigned due to overlap with 1,2-DFB solvent peaks.

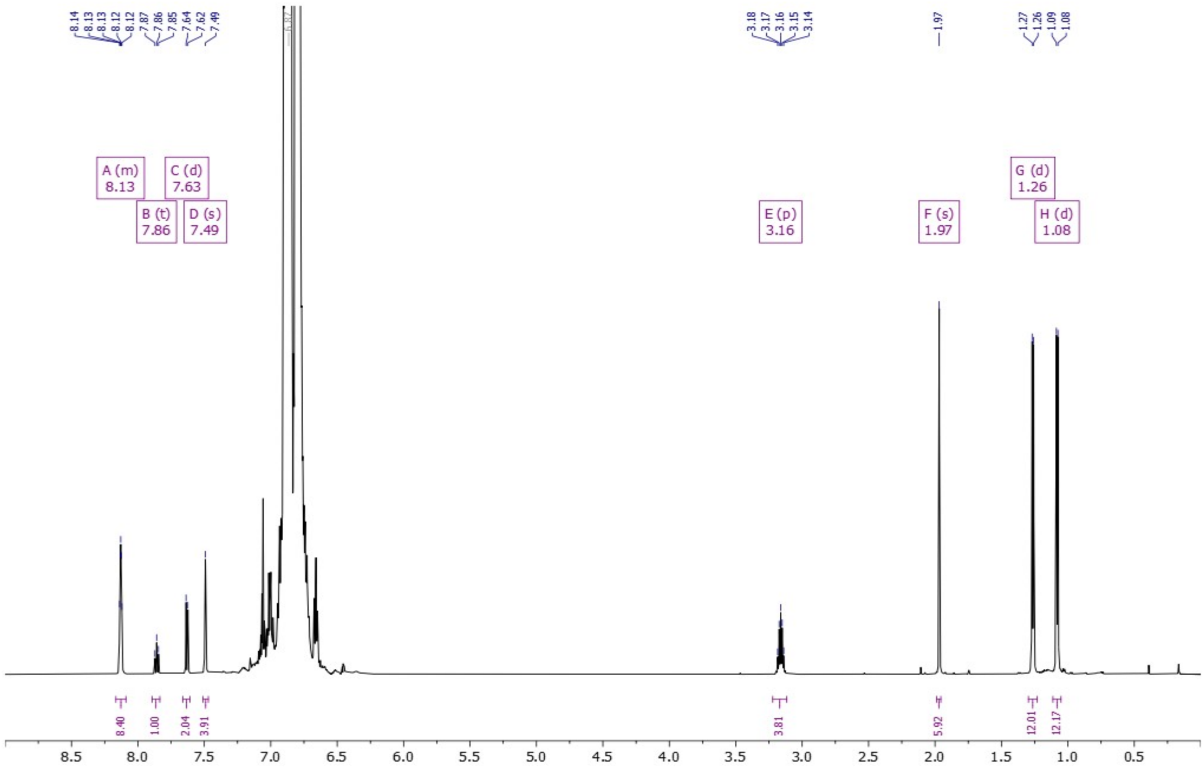

Figure S21. ^{1}H NMR (600 MHz) spectrum of $\mathbf{Rh_{CO}}^{+}$ in 1,2-DFB, locked to a C_6D_6 capillary.

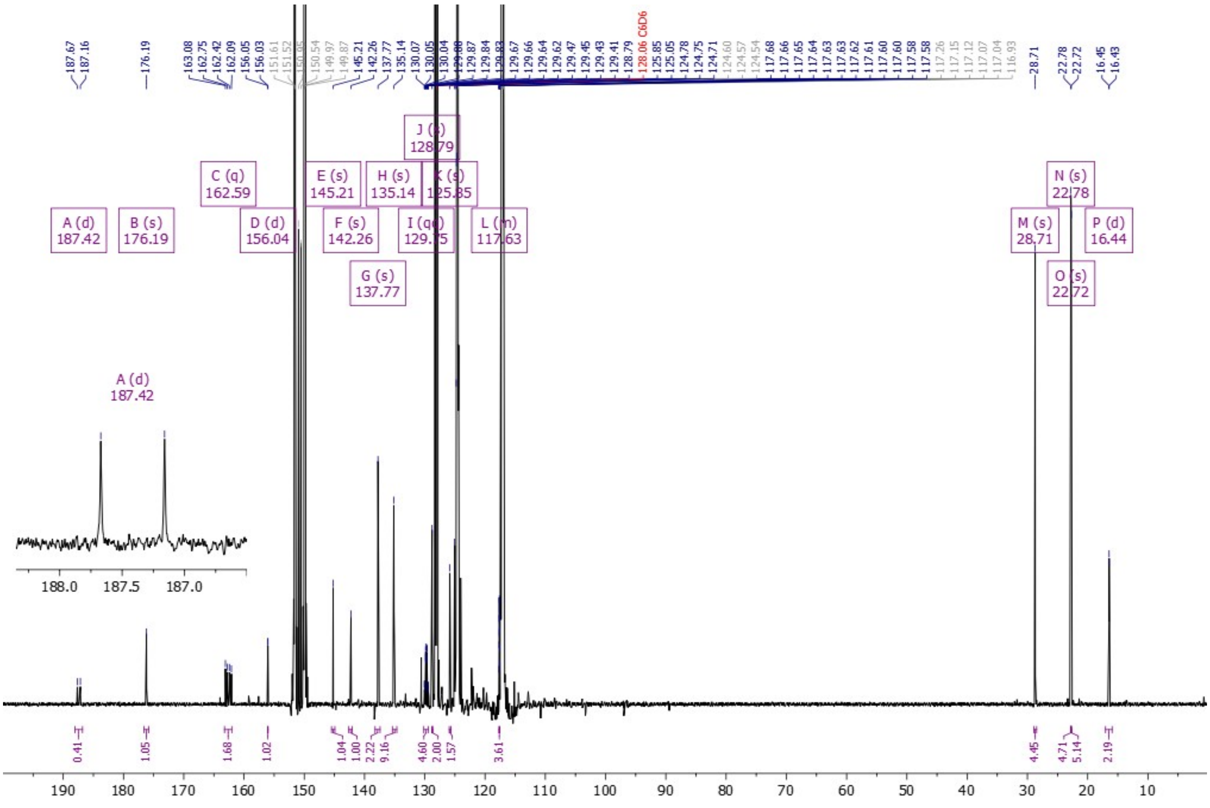

Figure S22. ^{13}C{^{1}H} NMR (600 MHz) spectrum of $\mathbf{Rh_{CO}}^{+}$ in 1,2-DFB, locked to a C_6D_6 capillary.

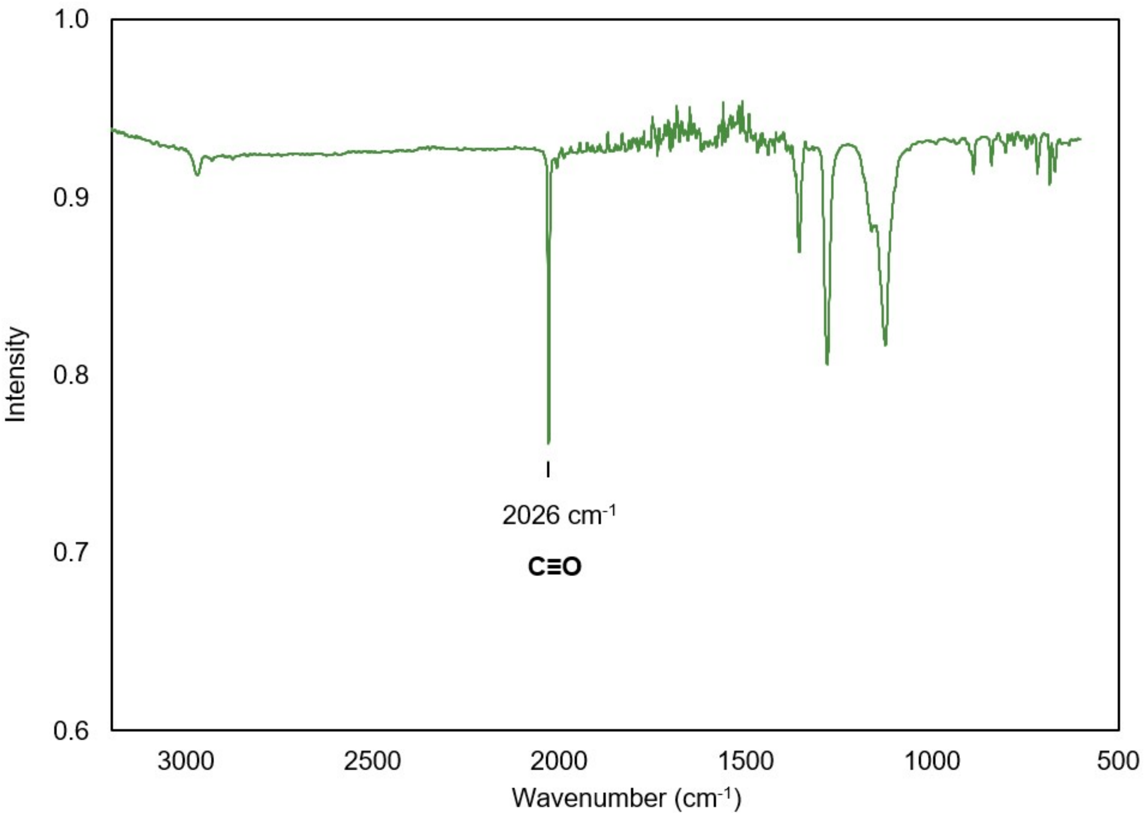

Figure S23. ATR-FTIR spectrum of **Rh$_{CO}$$^+$**.

1.2.9 Synthesis of [Rh(^{Dipp}PDI)(CN^{t}Bu)][BAr$^F_4$] (Rh$_{CNBu}$$^+$)

Rh(^{Dipp}PDI)Cl (50 mg, 0.08 mmol) and Na(BAr$^F_4$) (72 mg, 0.08 mmol) were dissolved in 1,2-DFB (3 mL). A solution of ^{t}BuNC (7 mg, 0.08 mmol) in DFB (0.5 mL) was added dropwise. The reaction mixture was stirred for 3 days, then filtered. The filtrate was concentrated, then layered with pentane (30 mL) for one week. After filtration, [Rh(^{Dipp}PDI)(CN^{t}Bu)][BAr$^F_4$] was obtained as dark green crystals. Yield: 101 mg, 0.07 mmol, 82%. Anal. Calcd. (%) for $C_{70}H_{64}N_4BF_{24}Rh$: C, 54.92; H, 4.21; N, 3.66. Found: C, 54.17; H, 3.60; N, 2.79.

^{1}H NMR (600 MHz, 1,2-DFB/C$_6$D$_6$): δ(ppm) 8.18–8.12 (m, 8H, BAr$^F_4$ *ortho* C**H**) 7.73 (t, $^3J_{H–H}$ = 8.1, 1H, pyridine *para* C**H**), 7.50 (bs, 4H, BAr$^F_4$ *para* C**H**), 7.46 (d, $^3J_{H–H}$ = 8.1, 2H, pyridine *meta* C**H**), 3.06 (sept, $^3J_{H–H}$ = 6.9 Hz, 4H, Dipp C**H**(CH$_3$)$_2$), 1.79 (s, 6H, imine NCC**H**$_3$), 1.17 (d, $^3J_{H–H}$ = 6.9 Hz, 12H, Dipp CH(C**H**$_3$)$_2$), 1.07 (d, $^3J_{H–H}$ = 6.9 Hz, 12H, Dipp CH(C**H**$_3$)$_2$), 0.70 (s, 9H, CNC(C**H**$_3$)$_3$). The signals for some aromatic protons could not be located due to overlap with 1,2-DFB solvent peaks.

^{13}C{^{1}H} NMR (151 MHz, 1,2-DFB/C$_6$D$_6$): δ(ppm) 178.60 (d, $^1J_{C–Rh}$ = 43.8 Hz, **C**N^{t}Bu), 172.01 (d, $^2J_{C–Rh}$ = 1.6 Hz, imine N**C**CH$_3$), 162.59 (q, $^1J_{C–B}$ = 49.8 Hz, BAr$^F_4$ *ipso* C), 156.01 (d, $^2J_{C–Rh}$ = 3.2 Hz, Dipp *ipso* **C**), 146.13 (s, Dipp *ortho* **C**), 138.58 (s, Ar**C**), 137.42 (s, Ar**C**), 135.14 (bs, BAr$^F_4$ *ortho* **C**), 130.29–129.35 (m, BAr$^F_4$ *meta* **C**), 127.65 (s, Ar**C**), 125.85 (s, Ar**C**), 117.71–

117.58 (m, BAr^F_4 *para* **C**), 56.95 (s, CN**C**(CH_3)$_3$), 29.27 (s, CNC(**C**H_3)$_3$), 28.47 (s, Dipp **C**H(CH_3)$_2$), 23.00 (s, Dipp **C**H(CH_3)$_2$), 22.77 (s, Dipp CH(**C**H_3)$_2$), 16.05 (s, imine NC**C**H_3). The signals for some aromatic carbons could not be located or were not assigned due to overlap with 1,2-DFB solvent peaks.

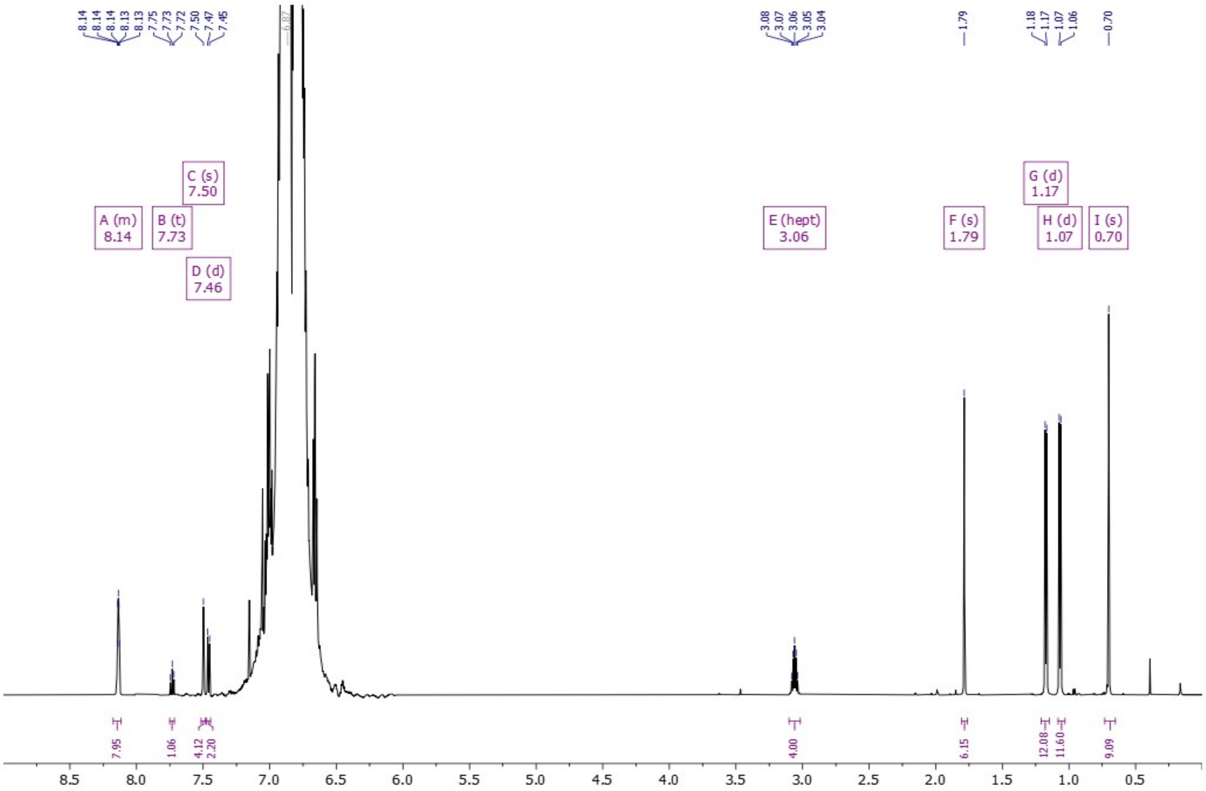

Figure S24. ^{1}H NMR (600 MHz) spectrum of **$Rh_{CNBu}$$^+$** in 1,2-DFB, locked to a C_6D_6 capillary.

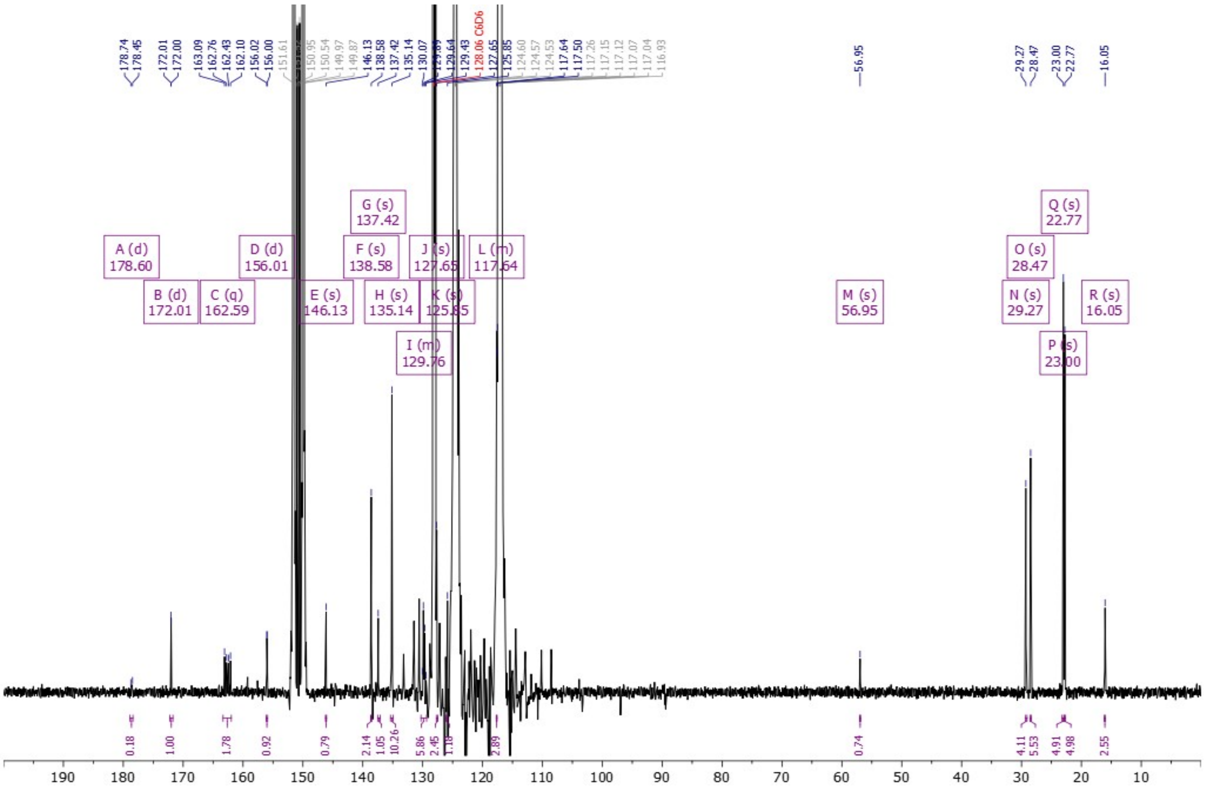

Figure S25. $^{13}C\{^{1}H\}$ NMR (600 MHz) spectrum of **Rh_{CNBu}^{+}** in 1,2-DFB, locked to a C_6D_6 capillary.

1.2.10 Synthesis of [Rh(^{Dipp}PDI)(C_2H_4)][$BAr^F{}_4$] (Rh_{ethene}^{+})

[Rh(^{Dipp}PDI)(C_2H_4)][$BAr^F{}_4$] was synthesized using a modified literature procedure.[7] Rh(^{Dipp}PDI)Cl (50 mg, 0.08 mmol) and Na($BAr^F{}_4$) (72 mg, 0.08 mmol) were dissolved in 1,2-DFB (3 mL). The solution was degassed, then placed under ethylene gas (1 bar). After stirring for 2 hours, the solution was degassed then filtered. The solution was concentrated, then layered with pentane (20 mL) for one week. After filtration, [Rh(^{Dipp}PDI)(C_2H_4)][$BAr^F{}_4$] was obtained as large dark crystals suitable for X-ray diffraction. Yield: 114 mg, 0.08 mmol, 96%.

^{1}H NMR (600 MHz, 1,2-DFB/C_6D_6): δ(ppm) 8.16–8.12 (m, 8H, $BAr^F{}_4$ *ortho* C**H**) 7.88 (t, $^{3}J_{H–H}$ = 8.1, 1H, pyridine *para* C**H**), 7.61 (d, $^{3}J_{H–H}$ = 8.1, 2H, pyridine *meta* C**H**), 7.49 (bs, 4H, $BAr^F{}_4$ *para* C**H**), 3.62 (d, $^{2}J_{H–Rh}$ = 1.6 Hz, 4H, C_2**H_4**), 2.93 (sept, $^{3}J_{H–H}$ = 6.8 Hz, 4H, Dipp C**H**(CH_3)$_2$), 1.85 (s, 6H, imine NCC**H_3**), 1.17 (d, $^{3}J_{H–H}$ = 6.9 Hz, 12H, Dipp CH(C**H_3**)$_2$), 0.96 (d, $^{3}J_{H–H}$ = 6.9 Hz, 12H, Dipp CH(C**H_3**)$_2$). The signals for some aromatic protons could not be located due to overlap with 1,2-DFB solvent peaks.

$^{13}C\{^{1}H\}$ NMR (151 MHz, 1,2-DFB/C_6D_6): δ(ppm) 174.84 (s, imine N**C**CH_3), 162.58 (q, $^{1}J_{C–B}$ = 49.8 Hz, $BAr^F{}_4$ *ipso* C), 155.40 (d, $^{2}J_{C–Rh}$ = 2.9 Hz, Dipp *ipso* **C**), 141.62 (s, Dipp *ortho* **C**), 139.72 (s, Ar**C**), 138.98 (s, Ar**C**), 135.14 (bs, $BAr^F{}_4$ *ortho* **C**), 129.75 (qq, $^{2}J_{C–F}$ = 31.5 Hz, $^{2}J_{C–B}$ = 3.0 Hz, $BAr^F{}_4$ *meta* **C**), 125.85 (s, Ar**C**), 117.72–117.56 (m, $BAr^F{}_4$ *para* **C**), 81.80 (d, $^{1}J_{C–Rh}$ = 10.1 Hz, **C**$_2H_4$), 28.41 (s, Dipp **C**H(CH_3)$_2$), 23.57 (s, Dipp **C**H(CH_3)$_2$), 22.59 (s, Dipp

CH(**C**H_3)$_2$), 18.05 (s, imine NC**C**H_3). The signals for some aromatic carbons could not be located or were not assigned due to overlap with 1,2-DFB solvent peaks.

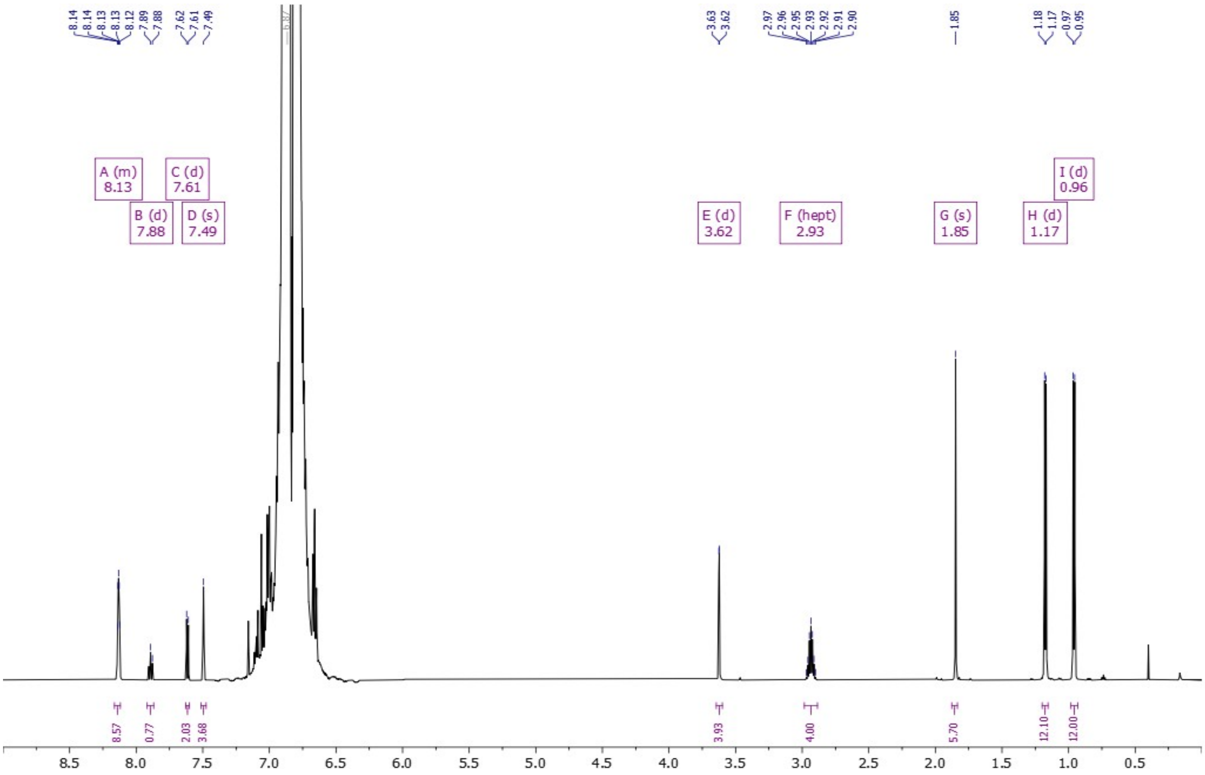

Figure S26. ^{1}H NMR (600 MHz) spectrum of **Rh$_{ethene}$$^+$** in 1,2-DFB, locked to a C_6D_6 capillary.

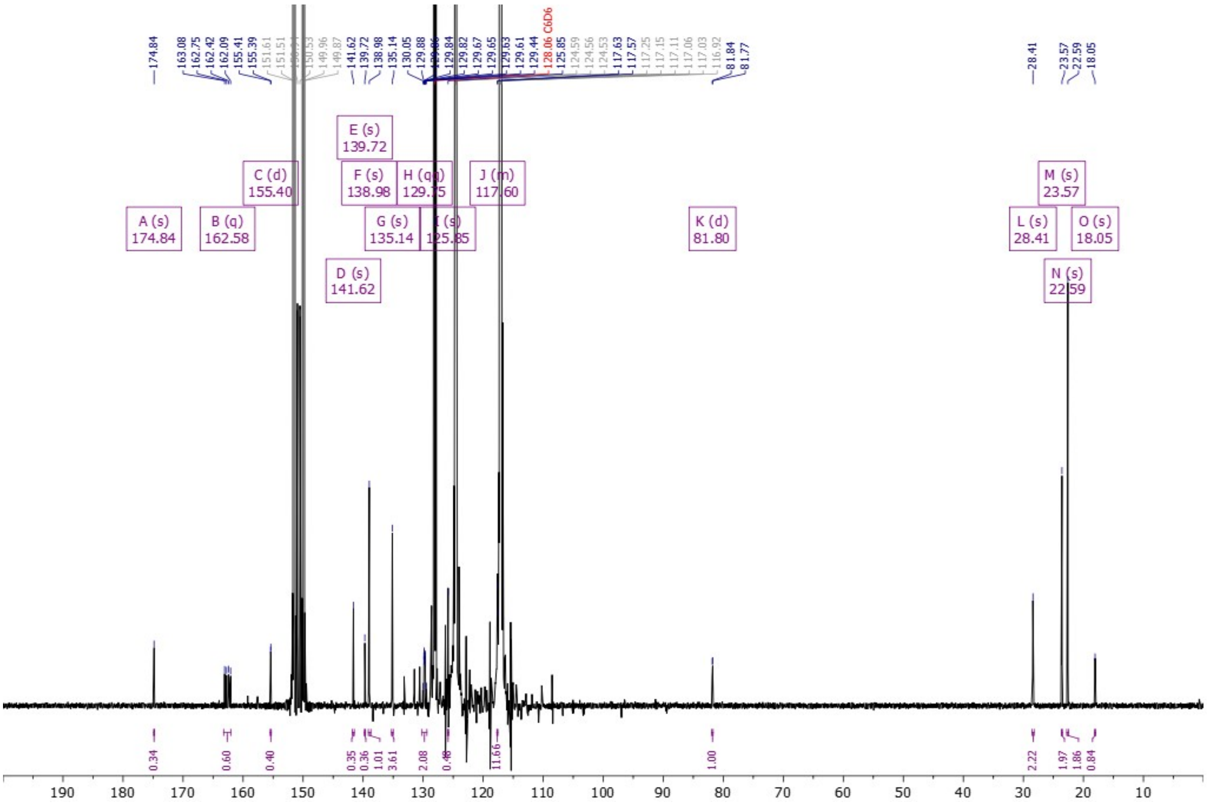

Figure S27. $^{13}C\{^{1}H\}$ NMR (600 MHz) spectrum of **Rh$_{ethene}$$^+$** in 1,2-DFB with a C_6D_6 capillary.

1.2.11 Attempted syntheses of Co(^{Dipp}PDI)(CN) (Co_{CN}) and Rh(^{Dipp}PDI)(CN) (Rh_{CN})

Method A: M(^{Dipp}PDI)Cl (M = Co, Rh) (1 eq.) and KCN (1 eq.) were suspended in THF. The reaction mixture was heated to reflux for 3 days, after which ^{1}H NMR spectroscopy showed only unreacted starting material.

Method B: M(^{Dipp}PDI)Cl (M = Co, Rh) (1 eq.) and KCN (1 eq.) and 18-crown-6 (1 eq.) were suspended in THF. The reaction mixture was heated to reflux for 3 days, after which ^{1}H NMR spectroscopy showed only unreacted starting material.

Method C: Rh(^{Dipp}PDI)Cl (1 eq.) and KCN (1 eq.) were dissolved in methanol. The reaction mixture was stirred for 1 h, after which ^{1}H NMR spectroscopy showed a mixture of products including free ^{Dipp}PDI ligand.

Method D: M(^{Dipp}PDI)Cl (M = Co, Rh) (1 eq.) was dissolved in toluene. A solution of Me_3SiCN in toluene (0.2 M, 1 eq.) was added dropwise at −78 ºC, resulting in discoloration of the solution to red (M = Co) or brown (M = Rh). After stirring overnight, ^{1}H NMR spectroscopy showed a mixture of products including free ^{Dipp}PDI ligand for M = Rh, and in the case of M = Co, unreacted Co(^{Dipp}PDI)Cl as well as several paramagnetic species.

Method E: M(^{Dipp}PDI)Cl (M = Co, Rh) (1 eq.) and $NaBAr^F_4$ (1 eq.) were dissolved in 1,2-DFB. A solution of $[Bu_4N]CN$ (1 eq.) in 1,2-DFB was added at −35 ºC, resulting in a color change to green (M = Co) or black (M = Rh). ^{1}H NMR spectroscopy showed a mixture of products, including unreacted M(^{Dipp}PDI)Cl.

Method F: Rh(^{Dipp}PDI)(C_2H_4) (1 eq.) was dissolved in 1,2-DFB. A solution of $[Bu_4N]CN$ (1 eq.) in 1,2-DFB was added at −35 ºC, resulting in an initial color change to bright green, then black. ^{1}H NMR spectroscopy showed a mixture of products including unreacted Rh(^{Dipp}PDI)Cl.

1.3 Other characterization data

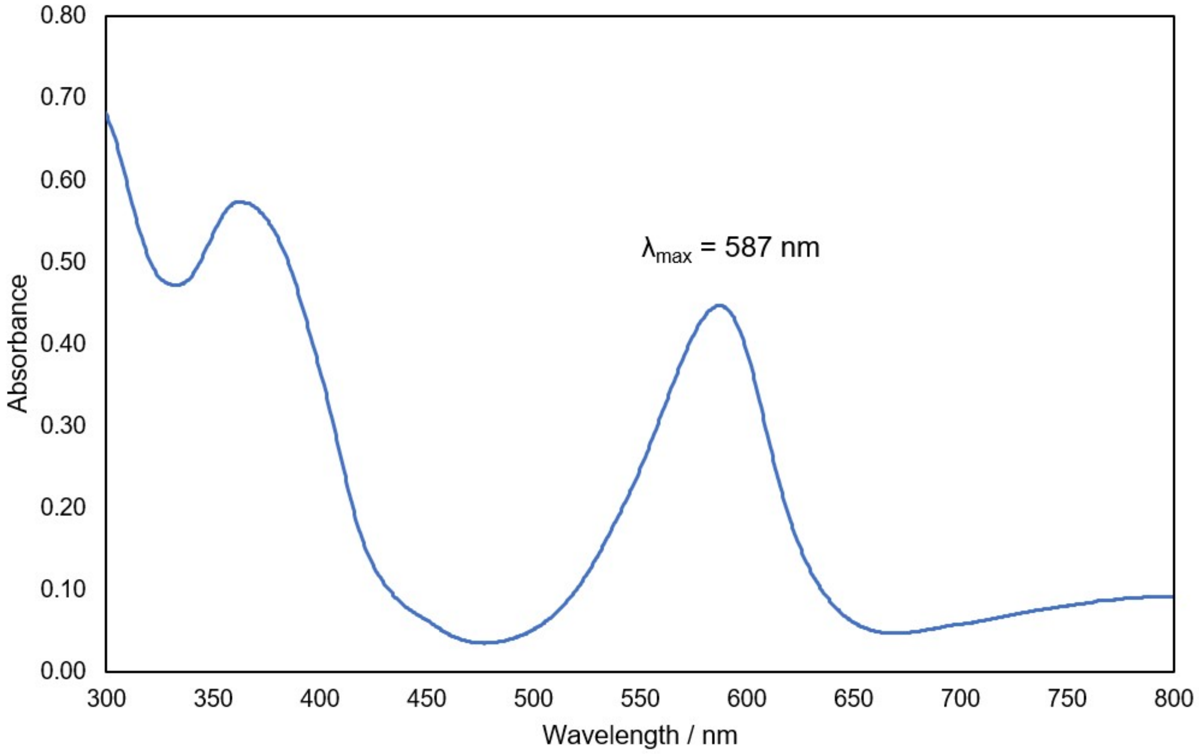

Figure S28. UV-vis absorption spectrum of Co(^{Dipp}PDI)(CP) in toluene.

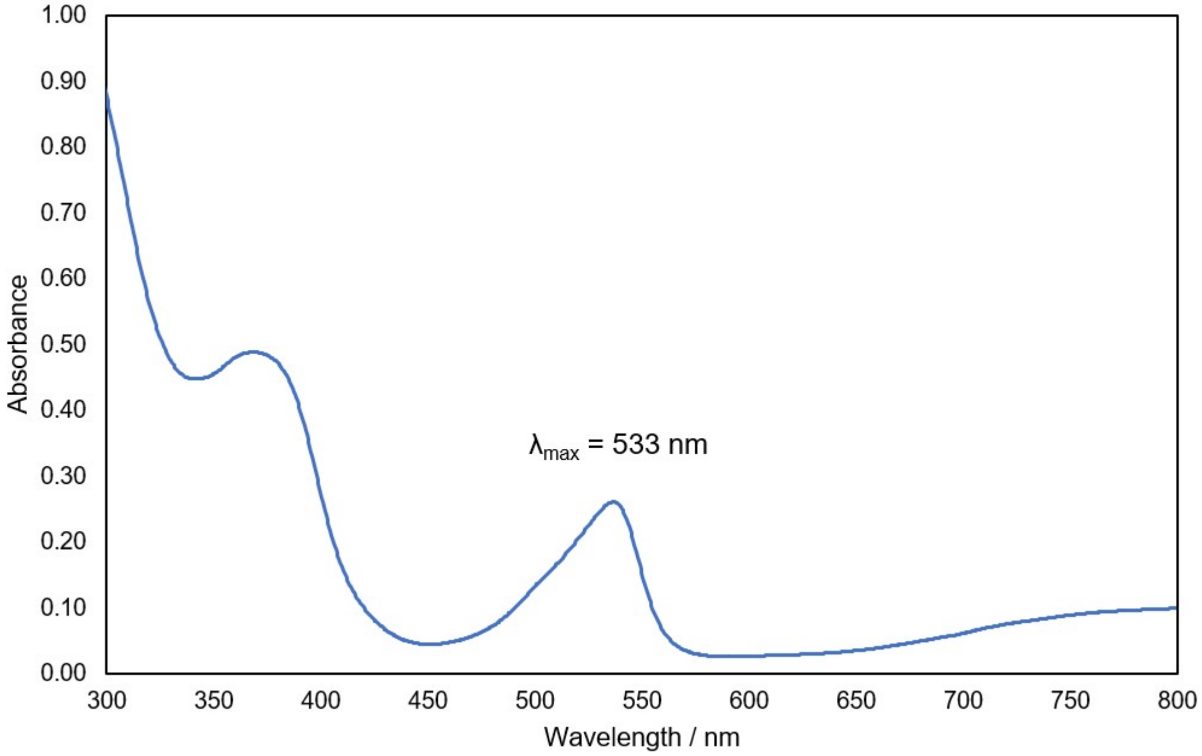

Figure S29. UV-vis absorption spectrum of Co(^{Dipp}PDI)Cl in toluene.

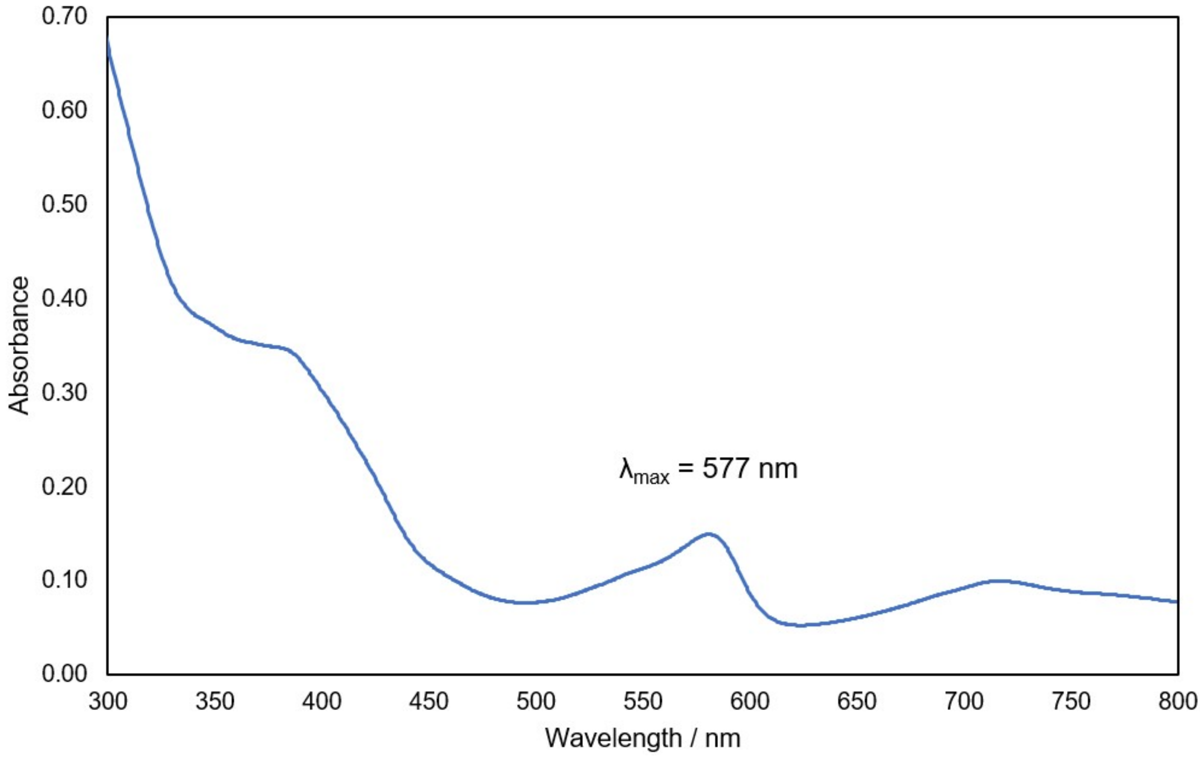

Figure S30. UV-vis absorption spectrum of Co(^{Dipp}PDI)Me in toluene.

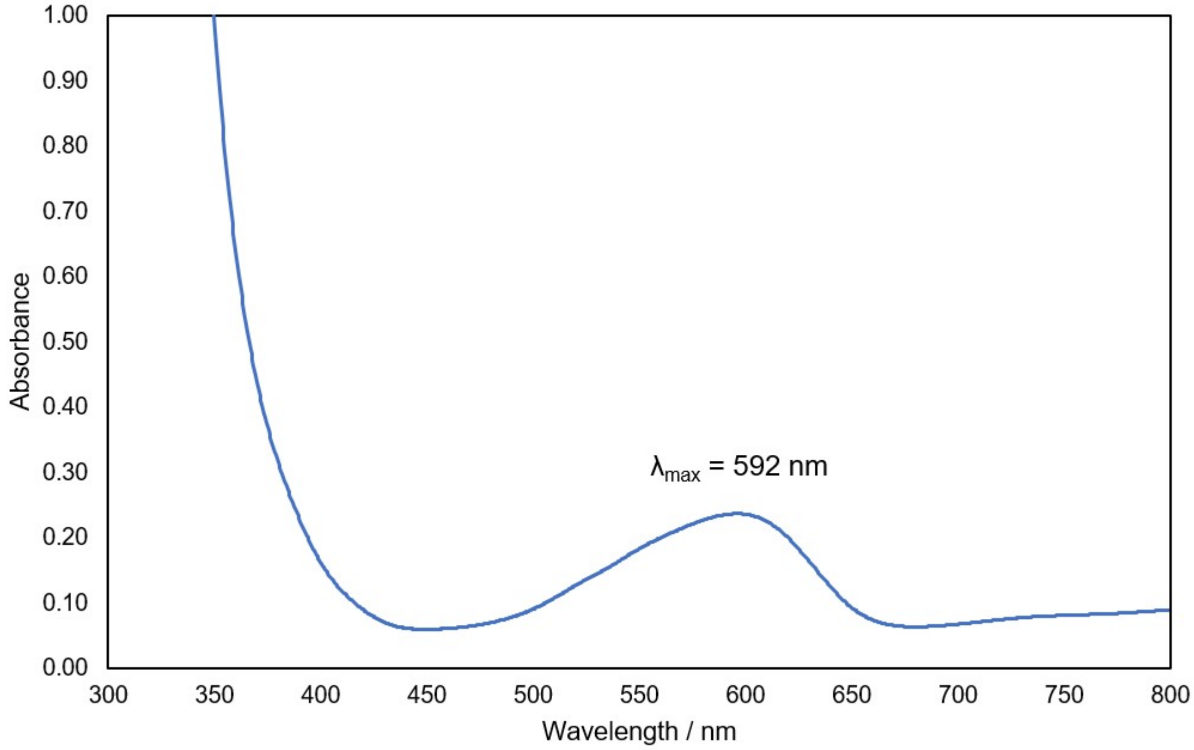

Figure S31. UV-vis absorption spectrum of [Co(^{Dipp}PDI)(N_2)][B(Me)(C_6F_5)$_3$] in toluene.

2. Single crystal X-ray diffraction data

Single-crystal X-ray diffraction data were collected using an Oxford Diffraction Supernova dual-source diffractometer equipped with a 135 mm Atlas CCD area detector. Crystals were selected under Paratone-N oil, mounted on micro-mount loops and quench-cooled using an Oxford Cryosystems open flow N_2 cooling device. Data were collected at 150 K using mirror monochromated Cu Kα (λ = 1.54184 Å) or Mo Kα (λ = 0.71073 Å) radiation and processed using the CrysAlisPro package, including unit cell parameter refinement and inter-frame scaling (carried out using SCALE3 ABSPACK).[8] Equivalent reflections were merged and diffraction patterns processed with the CrysAlisPro suite. Structures were subsequently solved using direct methods and refined on F^2 using the SHELXL package.[9]

Table S1. Selected X-ray data collection/refinement parameters for $\mathbf{Co_{CCH}}$, Na(THF)Co(PIEA)(CCH) and $\mathbf{Co_{CO}}^+(BAr^F{}_4)^-$.

	$\mathbf{Co_{CCH}}$	Na(THF)Co(PIEA)(CCH)	$\mathbf{Co_{CO}}^+(BAr^F{}_4)^-$
Formula	$C_{35}H_{44}CoN_3$	$C_{39}H_{51}CoN_3NaO$	$C_{66}H_{55}BCoF_{24}N_3O$
CCDC	2242900	2242901	2242902
Fw [g mol^{-1}]	565.66	659.74	1431.87
Crystal system	orthorhombic	monoclinic	monoclinic
Space group	$P2_12_12_1$	$P2_1/c$	$P2_1/c$
a (Å)	8.47050(10)	11.78850(10)	17.4943(5)
b (Å)	17.9094(3)	16.6101(2)	17.8665(6)
c (Å)	20.2338(3)	18.5455(2)	22.1081(6)
α (°)	90	90	90
β (°)	90	99.7630(10)	104.528(3)
γ (°)	90	90	90
V (Å^3)	3069.50(8)	3578.77(7)	6689.2(4)
Z	4	4	4
Radiation, λ (Å)	Mo K_α, 0.71073	Cu K_α, 1.54184	Mo K_α, 0.71073
Temp (K)	150(2)	150(2)	150(2)
ρ_{calc} (g cm^{-3})	1.224	1.224	1.422
μ (mm^{-1})	0.586	4.125	0.367
Reflections collected	54167	78701	107320
Indep. reflections	8746	7475	15340
Parameters	367	418	1007
R(int)	0.0472	0.0465	0.0566
R1/wR2,[a] I ≥ 2σI (%)	3.64/7.21	3.01/8.06	6.14/15.31
R1/wR2,[a] all data (%)	4.84/7.69	3.40/8.31	9.24/16.60
GOF	1.068	1.037	1.061

[a] $R1 = [\Sigma||F_o| - |F_c||]/\Sigma|F_o|$; $wR2 = \{[\Sigma w[(F_o)^2 - (F_c)^2]^2]/[\Sigma w(F_o{}^2)^2]\}^{1/2}$; $w = [\sigma^2(F_o)^2 + (AP)^2 + BP]^{-1}$, where $P = [(F_o)^2 + 2(F_c)^2]/3$ and the A and B values are 0.264 and 1.06 for $\mathbf{Co_{CCH}}$, 0.456 and 1.0611 for Na(THF)Co(PIEA)(CCH), and 0.0642 and 9.16 for $\mathbf{Co_{CO}}^+(BAr^F{}_4)^-$.

Table S2. Selected X-ray data collection/refinement parameters for **Co_{CNBu}**$^{+}$(BAr^{F}_{4})$^{-}$, **$Co2_{CNBu}$**$^{+}$(BAr^{F}_{4})$^{-}$ and **Rh_{Cl}**·1.33THF·0.66pent.

	**Co_{CNBu}**$^{+}$(BAr^{F}_{4})$^{-}$	**$Co2_{CNBu}$**$^{+}$(BAr^{F}_{4})$^{-}$	**Rh_{Cl}**·1.33THF·0.66pent
Formula	$C_{70}H_{64}BCoF_{24}N_4$	$C_{75}H_{73}BCoF_{24}N_5$	$C_{41.66}H_{61.66}ClN_3O_{1.33}Rh$
CCDC	2242903	2242904	2242905
Fw [g mol^{-1}]	1486.99	1570.12	764.30
Crystal system	triclinic	triclinic	orthorhombic
Space group	*P*–1	*P*–1	$C222_1$
a (Å)	14.3644(2)	12.9433(2)	17.36740(10)
b (Å)	15.5532(2)	15.1992(3)	29.7047(2)
c (Å)	16.5724(2)	20.7647(3)	23.95240(10)
α (°)	91.0850(10)	76.385(2)	90
β (°)	102.7870(10)	79.845(2)	90
γ (°)	99.3700(10)	87.162(2)	90
V (Å^3)	3556.66(8)	3907.87(12)	12356.88(12)
Z	2	2	12
Radiation, λ (Å)	Cu K_{α}, 1.54184	Cu K_{α}, 1.54184	Cu K_{α}, 1.54184
Temp (K)	150(2)	150(2)	150(2)
ρ_{calc} (g cm^{-3})	1.388	1.334	1.232
μ (mm^{-1})	2.835	2.613	4.200
Reflections collected	89994	44787	102303
Indep. reflections	14746	16152	12891
Parameters	954	1039	666
R(int)	0.0372	0.0355	0.0664
R1/wR2,[a] I ≥ 2σI (%)	4.58/12.42	5.22/13.83	3.80/9.81
R1/wR2,[a] all data (%)	4.85/12.66	6.09/14.70	4.15/10.20
GOF	1.077	1.044	1.036

[a] $R1 = [\Sigma||F_o| - |F_c||]/\Sigma|F_o|$; $wR2 = \{[\Sigma w[(F_o)^2 - (F_c)^2]^2]/[\Sigma w(F_o^2)^2]\}^{1/2}$; $w = [\sigma^2(F_o)^2 + (AP)^2 + BP]^{-1}$, where $P = [(F_o)^2 + 2(F_c)^2]/3$ and the A and B values are 0.0663 and 1.48 for **Co_{CNBu}**$^{+}$(BAr^{F}_{4})$^{-}$, 0.0818 and 0.98 for **$Co2_{CNBu}$**$^{+}$(BAr^{F}_{4})$^{-}$, 0.0599 and 14.42 for **Rh_{Cl}**·1.33THF·0.66pent.

Table S3. Selected X-ray data collection/refinement parameters for **Rh$_{Me}$**·0.25tol·0.25hex**,** **Rh$_{CP}$**, **Rh$_{CCH}$**.

	**Rh$_{Me}$**·0.25tol·0.25hex	**Rh$_{CP}$**	**Rh$_{CCH}$**
Formula	$C_{37.25}H_{51.5}N_3Rh$	$C_{34}H_{43}N_3PRh$	$C_{35}H_{44}N_3Rh$
CCDC	2242906	2242907	2242908
Fw [g mol^{-1}]	644.22	627.59	609.64
Crystal system	triclinic	orthorhombic	monoclinic
Space group	P–1	$P2_12_12_1$	$P2_1/n$
a (Å)	16.0874(11)	8.49660(10)	10.43020(10)
b (Å)	16.5041(10)	17.9973(2)	24.0268(2)
c (Å)	17.1430(8)	20.3635(2)	12.31850(10)
α (°)	86.593(4)	90	90
β (°)	64.600(5)	90	93.2010(10)
γ (°)	61.518(7)	90	90
V (Å^3)	3553.1(4)	3113.90(6)	3082.25(5)
Z	4	4	4
Radiation, λ (Å)	Cu K_α, 1.54184	Cu K_α, 1.54184	Cu K_α, 1.54184
Temp (K)	150(2)	150(2)	150(2)
ρ_{calc} (g cm^{-3})	1.204	1.339	1.314
μ (mm^{-1})	4.075	5.110	4.671
Reflections collected	39322	64682	17807
Indep. reflections	14641	6499	6385
Parameters	813	362	361
R(int)	0.0721	0.0453	0.0242
R1/wR2,[a] I ≥ 2σI (%)	5.74/14.90	2.14/5.62	2.92/7.41
R1/wR2,[a] all data (%)	7.42/16.93	2.25/5.69	3.02/7.48
GOF	1.031	1.068	1.102

[a] $R1 = [\Sigma||F_o| - |F_c||]/\Sigma|F_o|$; $wR2 = \{[\Sigma w[(F_o)^2 - (F_c)^2]^2]/[\Sigma w(F_o{}^2)^2]\}^{1/2}$; $w = [\sigma^2(F_o)^2 + (AP)^2 + BP]^{-1}$, where $P = [(F_o)^2 + 2(F_c)^2]/3$ and the A and B values are 0.0987 and 0.86 for **Rh$_{Me}$**·0.25tol·0.25hex, 0.036 and 0.80 for **Rh$_{CP}$**, 0.0398 and 2.42 for **Rh$_{CCH}$**.

Table S4. Selected X-ray data collection/refinement parameters for **Rh_{CO}**$^{+}$(BAr^{F}_{4})$^{–}$, **Rh_{CNBu}**$^{+}$(BAr^{F}_{4})$^{–}$ and **Rh_{ethene}**$^{+}$(BAr^{F}_{4})$^{–}$.

	**Rh_{CO}**$^{+}$(BAr^{F}_{4})$^{–}$	**Rh_{CNBu}**$^{+}$(BAr^{F}_{4})$^{–}$	**Rh_{ethene}**$^{+}$(BAr^{F}_{4})$^{–}$
Formula	$C_{66}H_{55}BF_{24}N_{3}ORh$	$C_{70}H_{64}BF_{24}N_{4}Rh$	$C_{67}H_{59}BF_{24}N_{3}Rh$
CCDC	2242909	2242910	2242911
Fw [g mol$^{–1}$]	1475.85	1530.97	1475.89
Crystal system	monoclinic	triclinic	monoclinic
Space group	$P2_1/c$	P–1	$P2_1/c$
a (Å)	17.5140(1)	14.4106(4)	17.5964(2)
b (Å)	17.7479(1)	15.5555(3)	17.6516(2)
c (Å)	22.3688(1)	16.6974(5)	22.6324(2)
α (°)	90	90.441(2)	90
β (°)	104.161(1)	103.544(2)	104.7830(10)
γ (°)	90	100.297(2)	90
V (Å^3)	6741.75(7)	3575.45(17)	6797.04(13)
Z	4	2	4
Radiation, λ (Å)	Cu K_α, 1.54184	Cu K_α, 1.54184	Cu K_α, 1.54184
Temp (K)	150(2)	150(2)	150(2)
ρ_{calc} (g cm$^{–3}$)	1.454	1.422	1.442
μ (mm$^{–1}$)	3.031	2.871	2.994
Reflections collected	48569	39212	41283
Indep. reflections	13941	14755	13995
Parameters	935	914	935
R(int)	0.0325	0.0382	0.0288
R1/wR2,[a] I ≥ 2σI (%)	4.11/10.80	4.17/10.85	4.77/12.86
R1/wR2,[a] all data (%)	4.59/11.28	4.47/11.17	5.46/13.66
GOF	1.060	1.019	1.041

[a] $R1 = [\Sigma||F_o| – |F_c||]/\Sigma|F_o|$; $wR2 = \{[\Sigma w[(F_o)^2 – (F_c)^2]^2]/[\Sigma w(F_o^2)^2]\}^{1/2}$; $w = [\sigma^2(F_o)^2 + (AP)^2 + BP]^{–1}$, where $P = [(F_o)^2 + 2(F_c)^2]/3$ and the A and B values are 0.0579 and 6.52 and for **Rh_{CO}**$^{+}$(BAr^{F}_{4})$^{–}$, 0.0629 and 3.07 for **Rh_{CNBu}**$^{+}$(BAr^{F}_{4})$^{–}$, and 0.0792 and 6.55 for **Rh_{ethene}**$^{+}$(BAr^{F}_{4})$^{–}$.

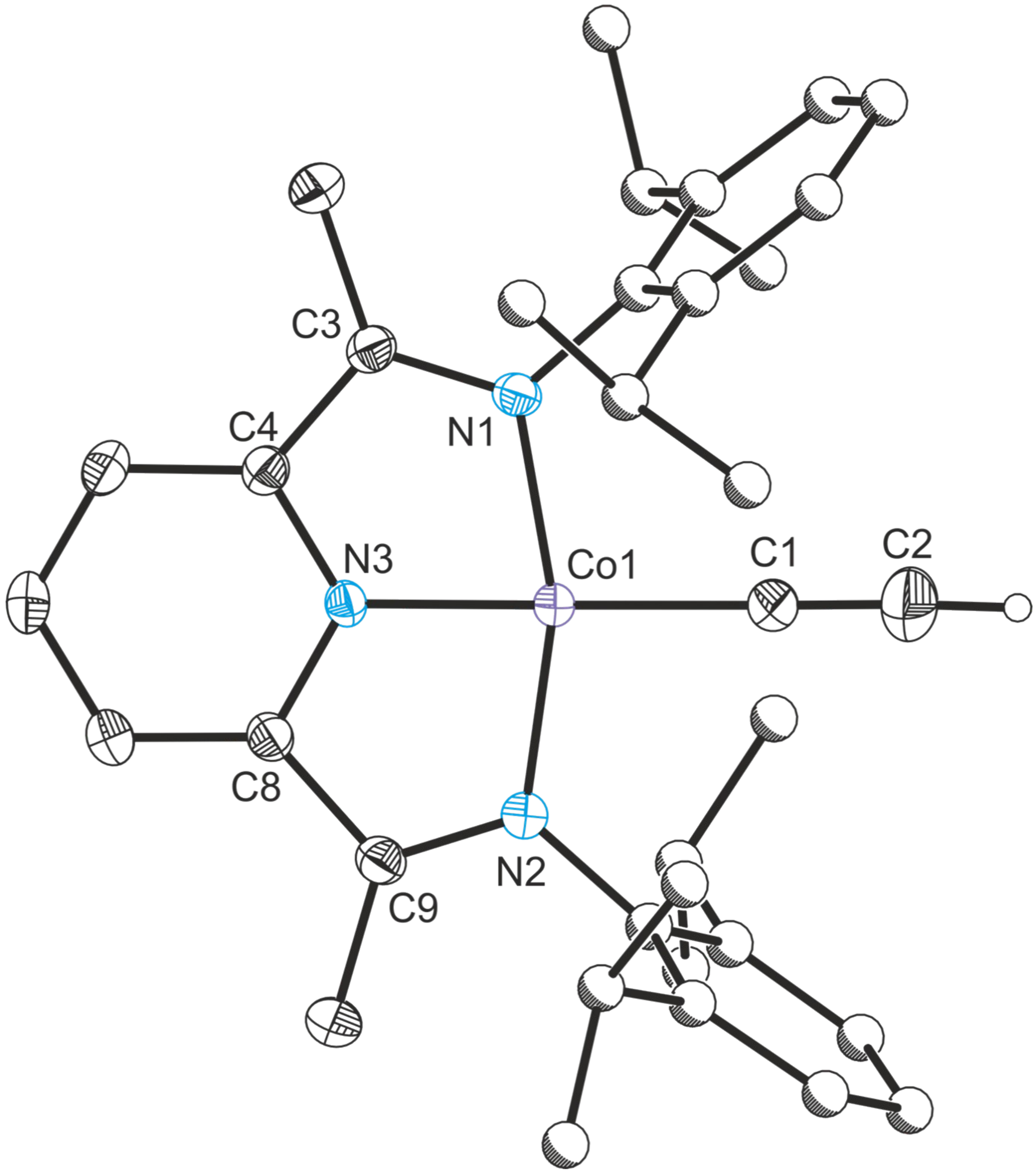

Figure S32. Single crystal X-ray structure of **Co$_{CCH}$**. Thermal ellipsoids displayed at 50% probability. Atoms of Dipp groups pictured as spheres of arbitrary radius. Hydrogen atoms (with the exception of that bonded to C2) have been removed for clarity. Selected bond distances (Å) and angles (°): Co1–C1 1.906(3), C1–C2 1.192(4), Co1–N1 1.906(2), Co1–N2 1.904(2), Co1–N3 1.8117(19), N1–C3 1.319(3), C3–C4 1.442(3), C4–N3 1.367(3), N3–C8 1.374(3), C8–C9 1.445(3), C9–N2 1.321(3); C1-Co1-N1 98.14(10), C1-Co1-N2 99.28(10), C1-Co1-N3 179.31(11), N1-Co1-N2 162.54(8), N1-Co1-N3 81.38(9), Ni2-Co1-N3, 81.19(9).

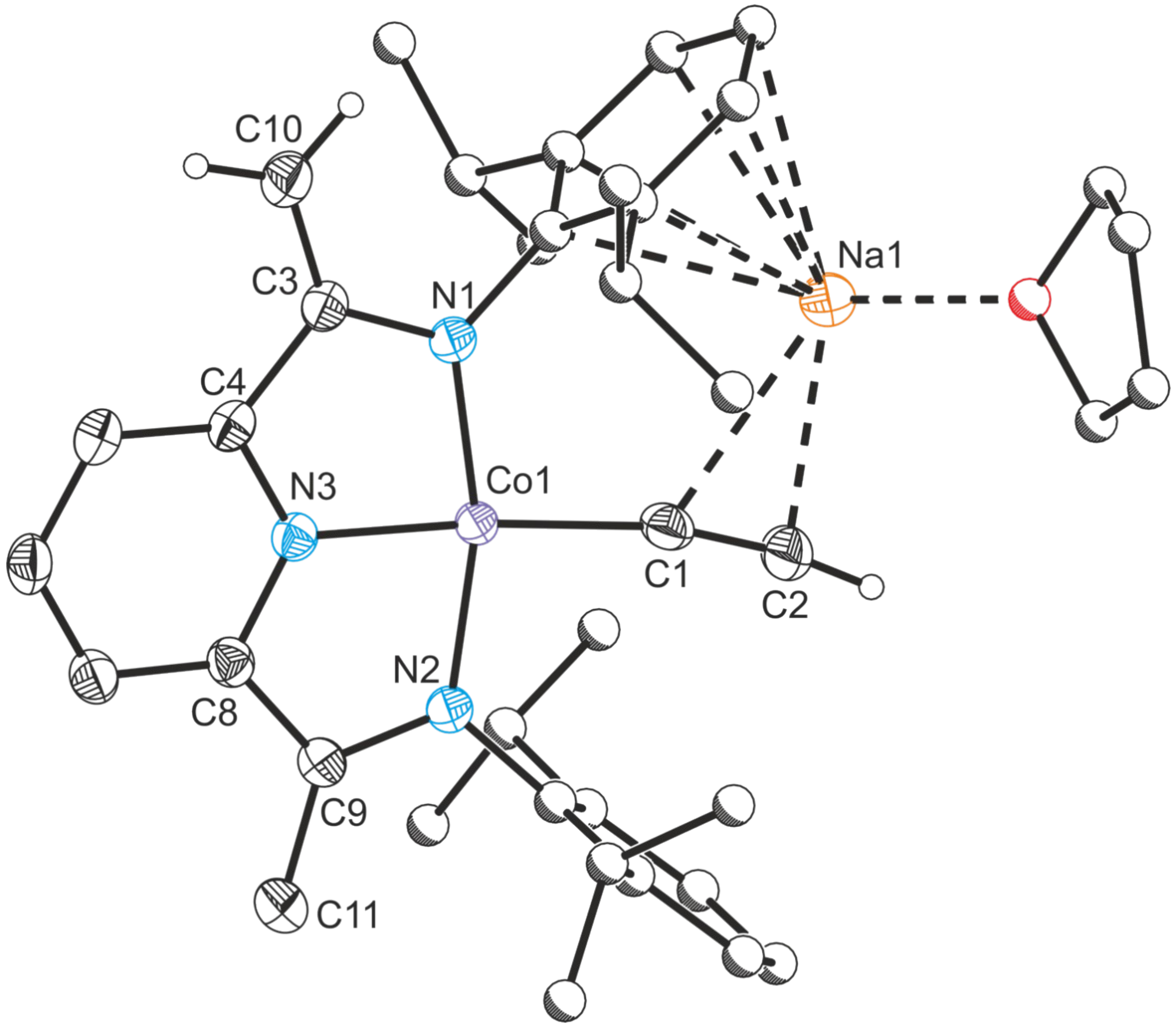

Figure S33. Single crystal X-ray structure of Na(THF)Co(PIEA)(CCH). Thermal ellipsoids displayed at 50% probability. Atoms of Dipp groups and THF molecule pictured as spheres of arbitrary radius. Hydrogen atoms (with the exception of that bonded to C2) have been removed for clarity. Selected bond distances (Å) and angles (°): Co1–C1 1.8970(15), C1–C2 1.212(2), Co1–N1 1.8759(11), Co1–N2 1.8686(11), Co1–N3 1.8399(11), N1–C3 1.3866(17), C3–C10 1.378(2), C3–C4 1.4681(19), C4–N3 1.3561(17), N3–C8 1.3678(17), C8–C9 1.4281(19), C9–C11 1.4680(19), C9–N2 1.3614(18); C1-Co1-N1 101.89(5), C1-Co1-N2 93.89(5), C1-Co1-N3 174.24(6), N1-Co1-N2 164.19(5), N1-Co1-N3 82.28(5), Ni2-Co1-N3, 81.91(5).

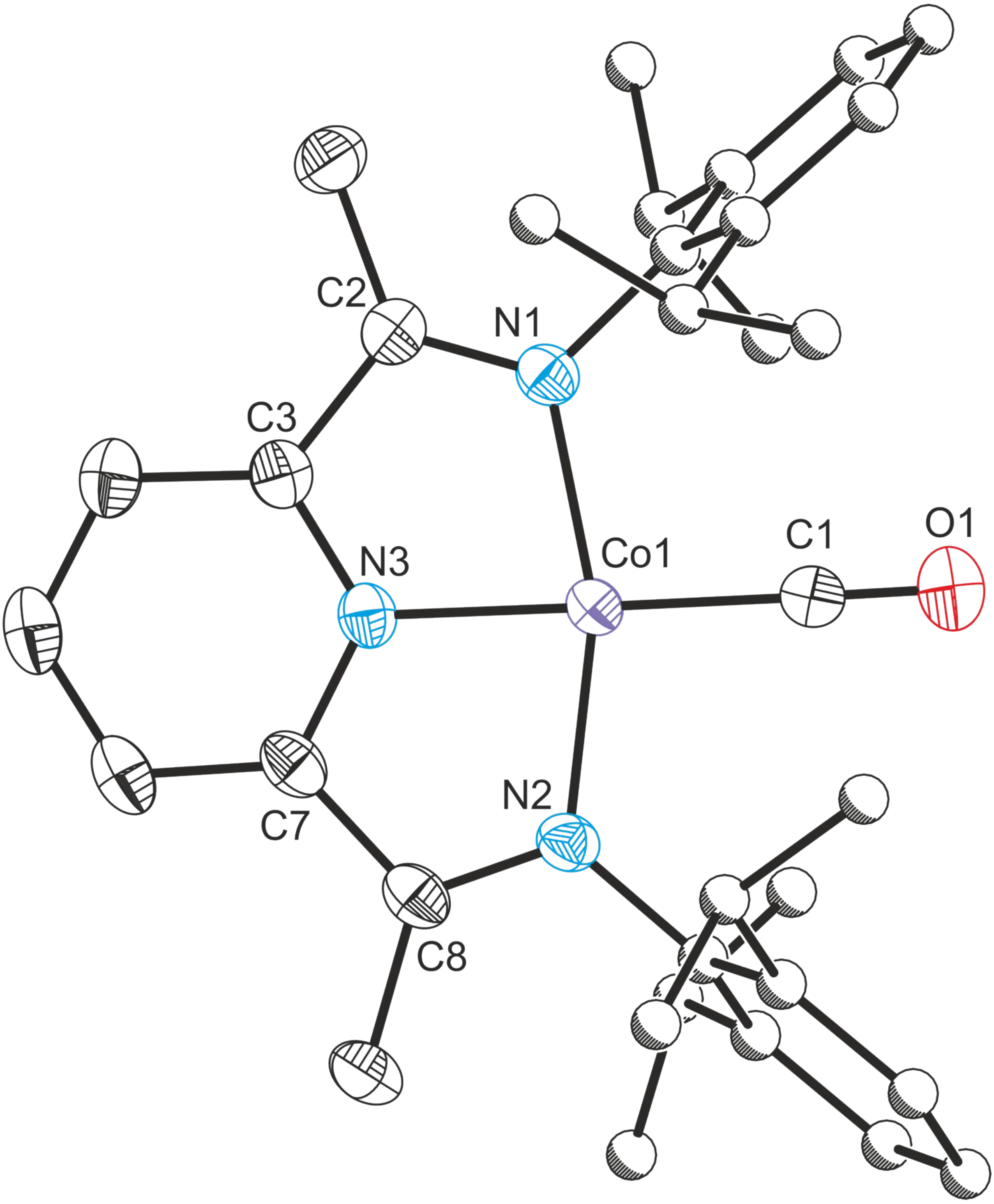

Figure S34. Single crystal X-ray structure of $\mathbf{Co_{CO}}^+$. Thermal ellipsoids displayed at 50% probability. Atoms of Dipp groups pictured as spheres of arbitrary radius. Hydrogen atoms have been removed for clarity. Selected bond distances (Å) and angles (°): Co1–C1 1.773(3), C1–O1 1.129(4), Co1–N1 1.919(2), Co1–N2 1.920(2), Co1–N3 1.847(2), N1–C2 1.300(3), C2–C3 1.473(4), C3–N3 1.343(3), N3–C7 1.346(3), C7–C8 1.462(4), C8–N2 1.307(3); C1-Co1-N1 98.67(11), C1-Co1-N2 99.09(11), C1-Co1-N3 174.59(13), N1-Co1-N2 162.17(10), N1-Co1-N3 80.73(9), Ni2-Co1-N3, 81.43(10).

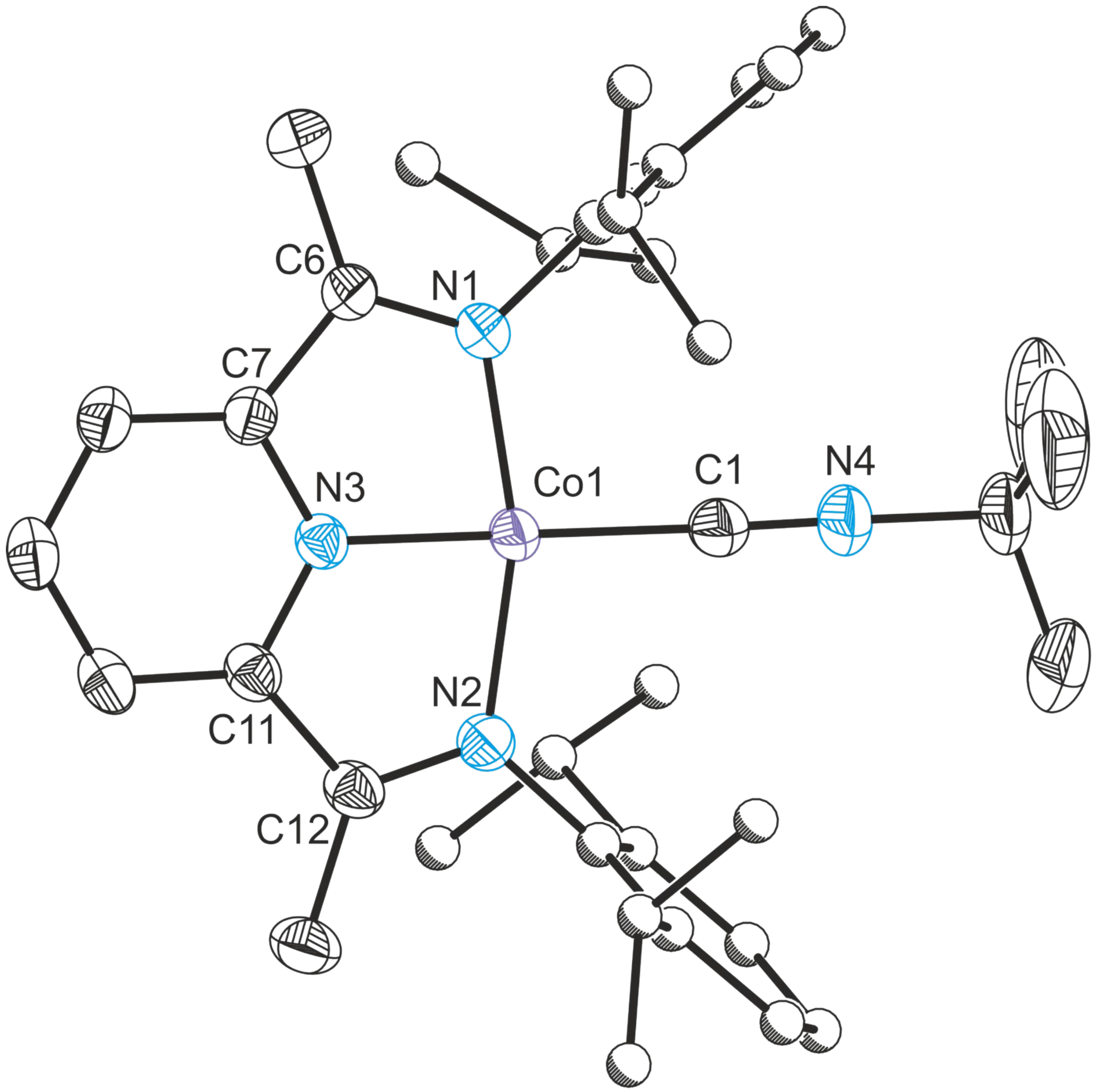

Figure S35. Single crystal X-ray structure of $\mathbf{Co_{CNBu}}^{+}$. Thermal ellipsoids displayed at 50% probability. Atoms of Dipp groups pictured as spheres of arbitrary radius. Hydrogen atoms have been removed for clarity. Selected bond distances (Å) and angles (°): Co1–C1 1.8755(19), C1–N4 1.154(3), Co1–N1 1.9072(14), Co1–N2 1.9198(15), Co1–N3 1.8186(14), N1–C6 1.312(2), C6–C7 1.451(2), C7–N3 1.357(2), N3–C11 1.353(2), C11–C12 1.457(2), C12–N2 1.309(2); C1-Co1-N1 97.83(7), C1-Co1-N2 99.30(7), C1-Co1-N3 171.81(8), N1-Co1-N2 162.44(6), N1-Co1-N3 81.03(6), Ni2-Co1-N3, 81.46(6).

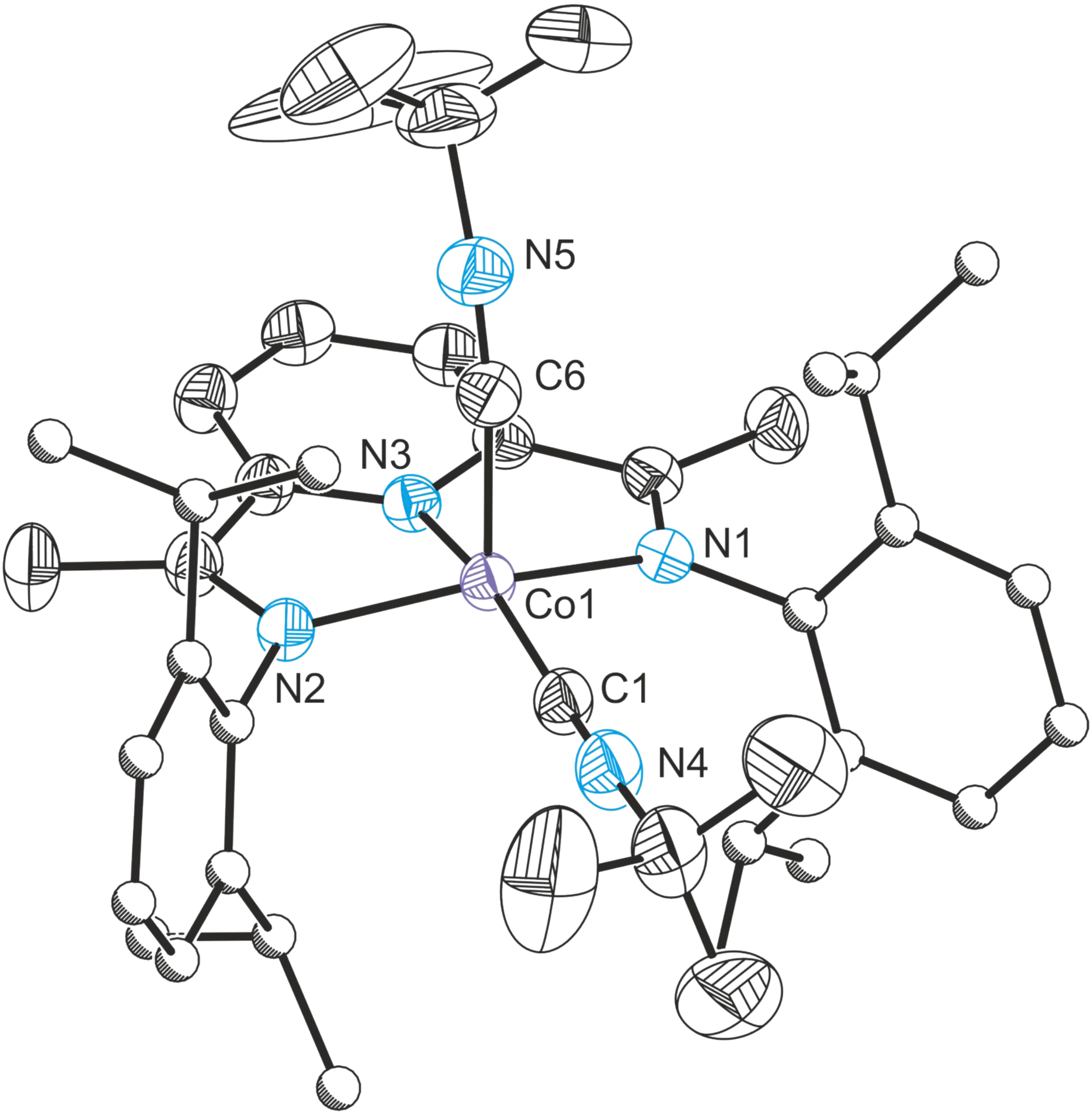

Figure S36. Single crystal X-ray structure of **Co_{2CNBu}^{+}**. Thermal ellipsoids displayed at 50% probability. Atoms of Dipp groups pictured as spheres of arbitrary radius. Hydrogen atoms have been removed for clarity. Selected bond distances (Å) and angles (°): Co1–C1 1.872(2), C1–N4 1.147(3), Co1–C6 1.918(2), C6–N5 1.146(3), Co1–N1 1.9329(16), Co1–N2 1.9319(16), Co1–N3 1.8194(16); C1-Co1-N1 97.75(8), C1-Co1-N2 97.04(8), C1-Co1-N3 166.86(9), C1-Co1-C6, 102.43(9), N1-Co1-N2 155.87(7), N1-Co1-N3 80.78(7), N1-Co1-C6 96.63(8), Ni2-Co1-N3, 80.48(7), N2-Co1-C6 98.67(8), N3-Co1-C6 90.70(8).

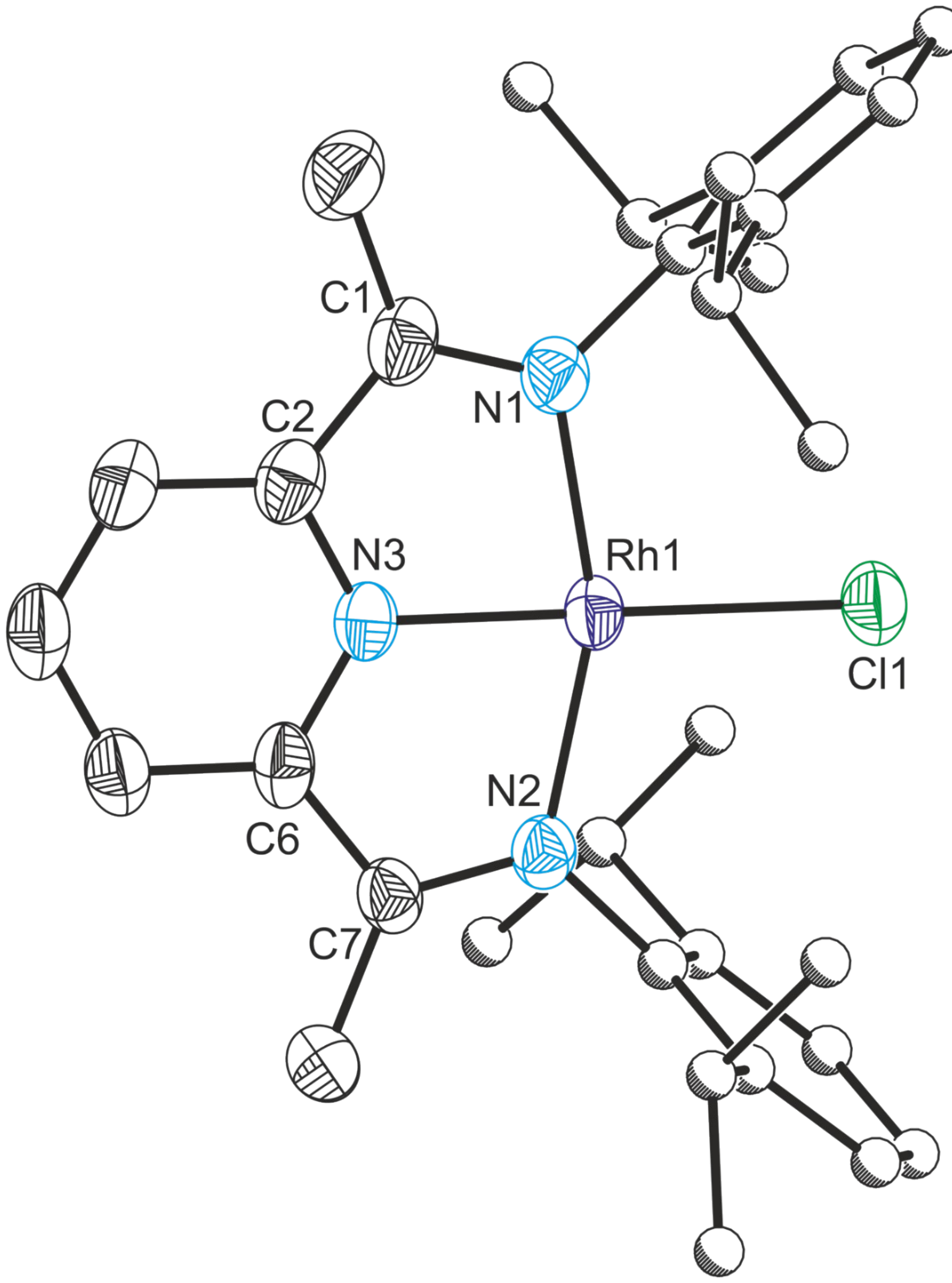

Figure S37. Single crystal X-ray structure of **Rh_{Cl}**. Thermal ellipsoids displayed at 50% probability. Atoms of Dipp groups pictured as spheres of arbitrary radius. Hydrogen atoms have been removed for clarity. Selected bond distances (Å) and angles (°): Rh1–Cl1 2.3191(12), Rh1–N1 2.032(4), Rh1–N2 2.036(4), Rh1–N3 1.897(4), N1–C1 1.310(7), C1–C2 1.467(8), C2–N3 1.365(6), N3–C6 1.353(7), C6–C7 1.455(7), C7–N2 1.311(6); Cl1-Rh1-N1 100.08(13), Cl1-Rh1-N2 100.85(12), Cl1-Rh1-N3 179.38(13), N1-Rh1-N2 159.06(16), N1-Rh1-N3 80.03(17), N2-Rh1-N3, 79.05(17).

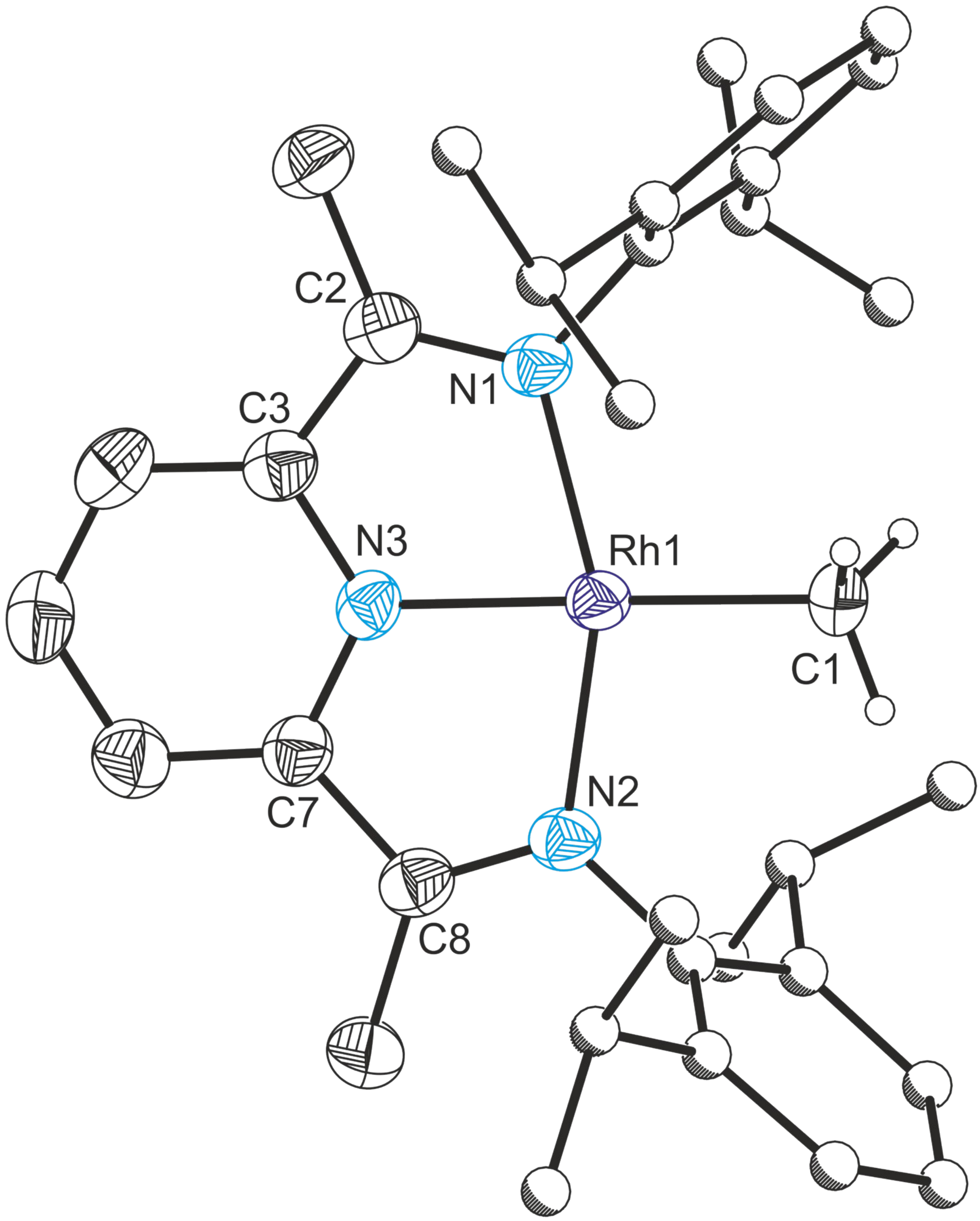

Figure S38. Single crystal X-ray structure of **Rh$_{Me}$**. Thermal ellipsoids displayed at 50% probability. Atoms of Dipp groups pictured as spheres of arbitrary radius. Hydrogen atoms (with the exception of those bonded to C1) have been removed for clarity. Selected bond distances (Å) and angles (°): Rh1–C1 2.074(4), Rh1–N1 2.015(3), Rh1–N2 2.022(3), Rh1–N3 1.936(3), N1–C2 1.329(6), C2–C3 1.454(6), C3–N3 1.364(5), N3–C7 1.367(6), C7–C8 1.465(6), C8–N2 1.310(6); C1-Rh1-N1 101.41(16), C1-Rh1-N2 101.14(16), C1-Rh1-N3 78.48(14), N1-Rh1-N2 157.31(15), N1-Rh1-N3 78.48(14), N2-Rh1-N3, 78.84(14).

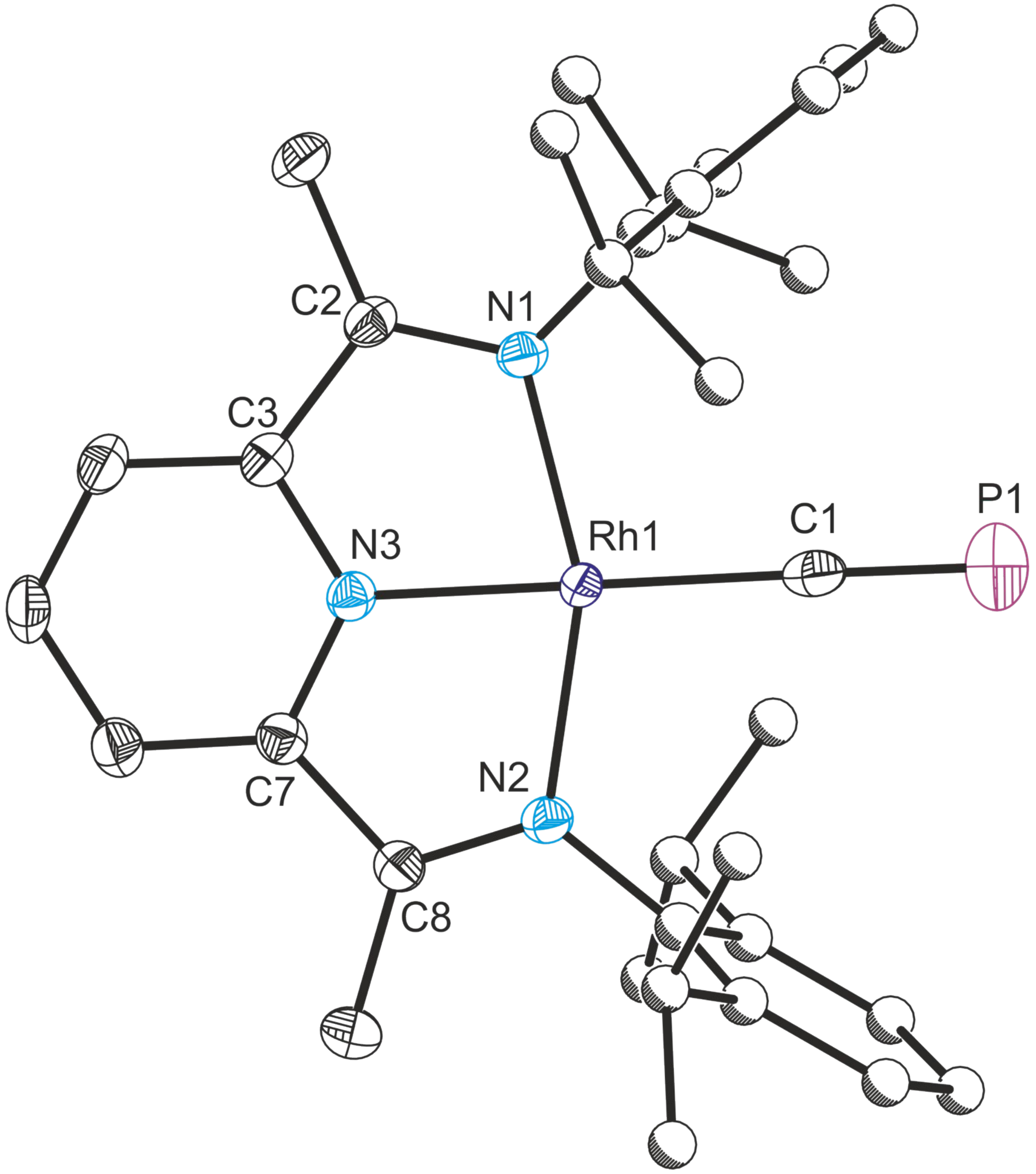

Figure S39. Single crystal X-ray structure of **Rh$_{CP}$**. Thermal ellipsoids displayed at 50% probability. Atoms of Dipp groups pictured as spheres of arbitrary radius. Hydrogen atoms have been removed for clarity. Selected bond distances (Å) and angles (°): Rh1–C1 1.969(3), C1–P1 1.542(4), Rh1–N1 2.019(2), Rh1–N2 2.023(2), Rh1–N3 1.942(2), N1–C2 1.309(4), C2–C3 1.470(4), C3–N3 1.360(4), N3–C7 1.357(4), C7–C8 1.468(4), C8–N2 1.315(4); C1-Rh1-N1 101.06(11), C1-Rh1-N2 101.41(11), C1-Rh1-N3 179.24(14), N1-Rh1-N2 157.48(10), N1-Rh1-N3 78.92(10), N2-Rh1-N3, 78.59(10).

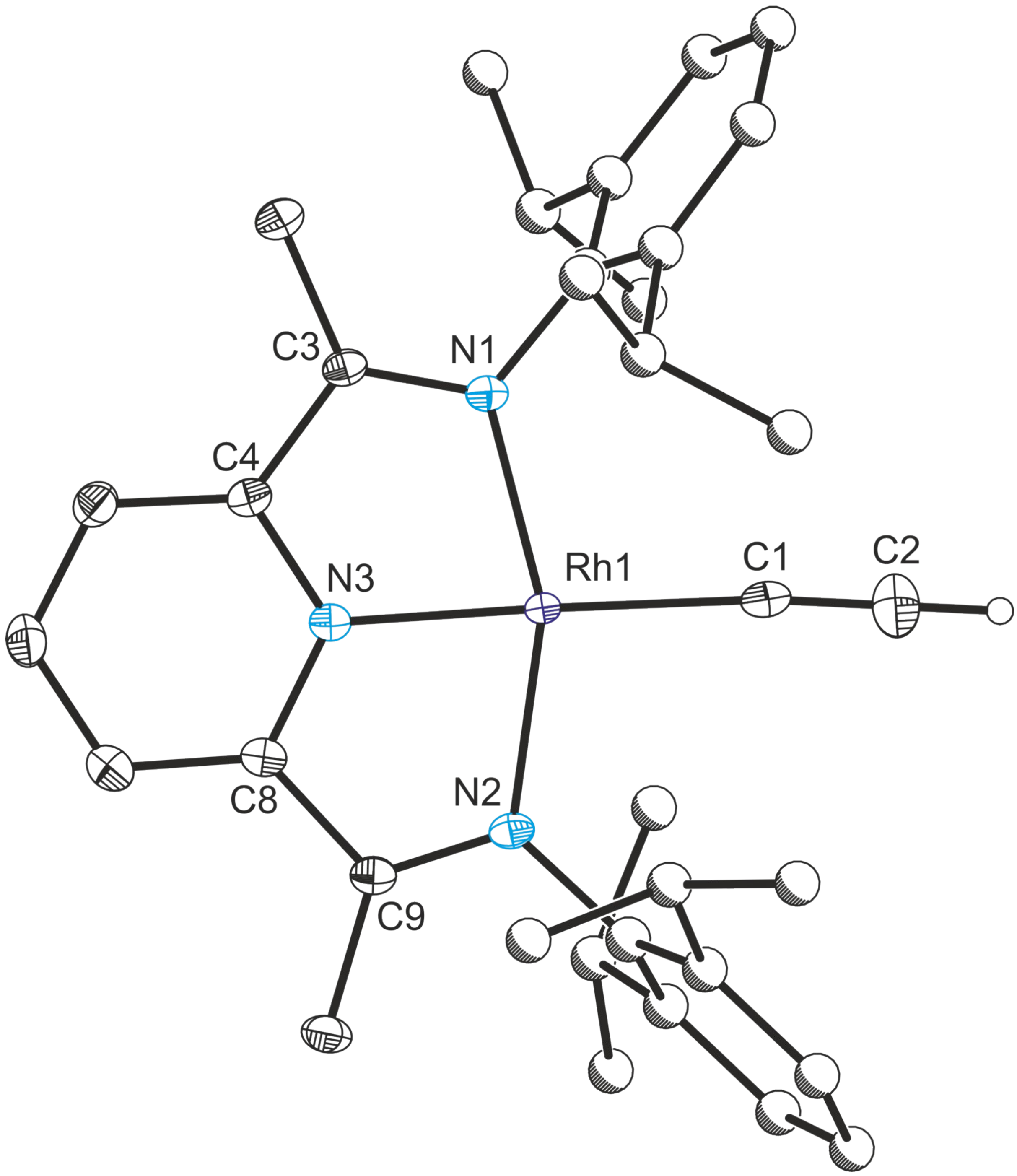

Figure S40. Single crystal X-ray structure of **Rh_{CCH}**. Thermal ellipsoids displayed at 50% probability. Atoms of Dipp groups pictured as spheres of arbitrary radius. Hydrogen atoms (with the exception of that bonded to C2) have been removed for clarity. Selected bond distances (Å) and angles (°): Rh1–C1 2.025(2), C1–C2 1.188(3), Rh1–N1 2.0297(16), Rh1–N2 2.0378(16), Rh1–N3 1.9262(16), N1–C3 1.312(3), C3–C4 1.460(3), C4–N3 1.361(2), N3–C8 1.366(2), C8–C9 1.464(3), C9–N2 1.313(3); C1-Rh1-N1 103.08(7), C1-Rh1-N2 99.49(7), C1-Rh1-N3 177.18(7), N1-Rh1-N2 157.38(6), N1-Rh1-N3 78.78(6), N2-Rh1-N3, 78.73(7).

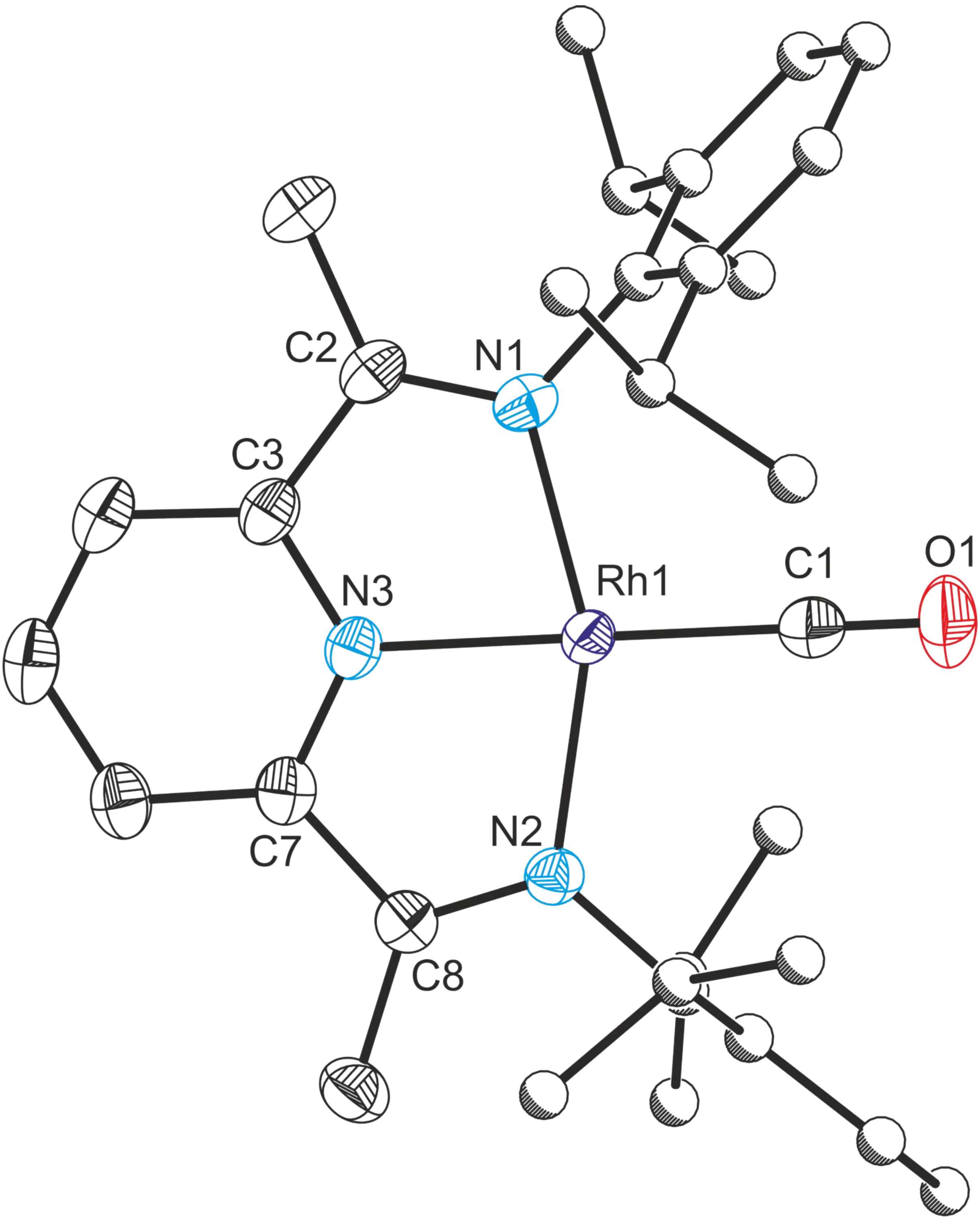

Figure S41. Single crystal X-ray structure of **Rh_{CO}^{+}**. Thermal ellipsoids displayed at 50% probability. Atoms of Dipp groups pictured as spheres of arbitrary radius. Hydrogen atoms have been removed for clarity. Selected bond distances (Å) and angles (°): Rh1–C1 1.870(3), C1–O1 1.135(3), Rh1–N1 2.0442(19), Rh1–N2 2.0427(19), Rh1–N3 1.9684(19), N1–C2 1.301(3), C2–C3 1.482(3), C3–N3 1.341(3), N3–C7 1.343(3), C7–C8 1.484(3), C8–N2 1.302(3); C1-Rh1-N1 101.71(9), C1-Rh1-N2 101.07(9), C1-Rh1-N3 178.10(11), N1-Rh1-N2 157.22(8), N1-Rh1-N3 78.75(8), N2-Rh1-N3, 78.49(8).

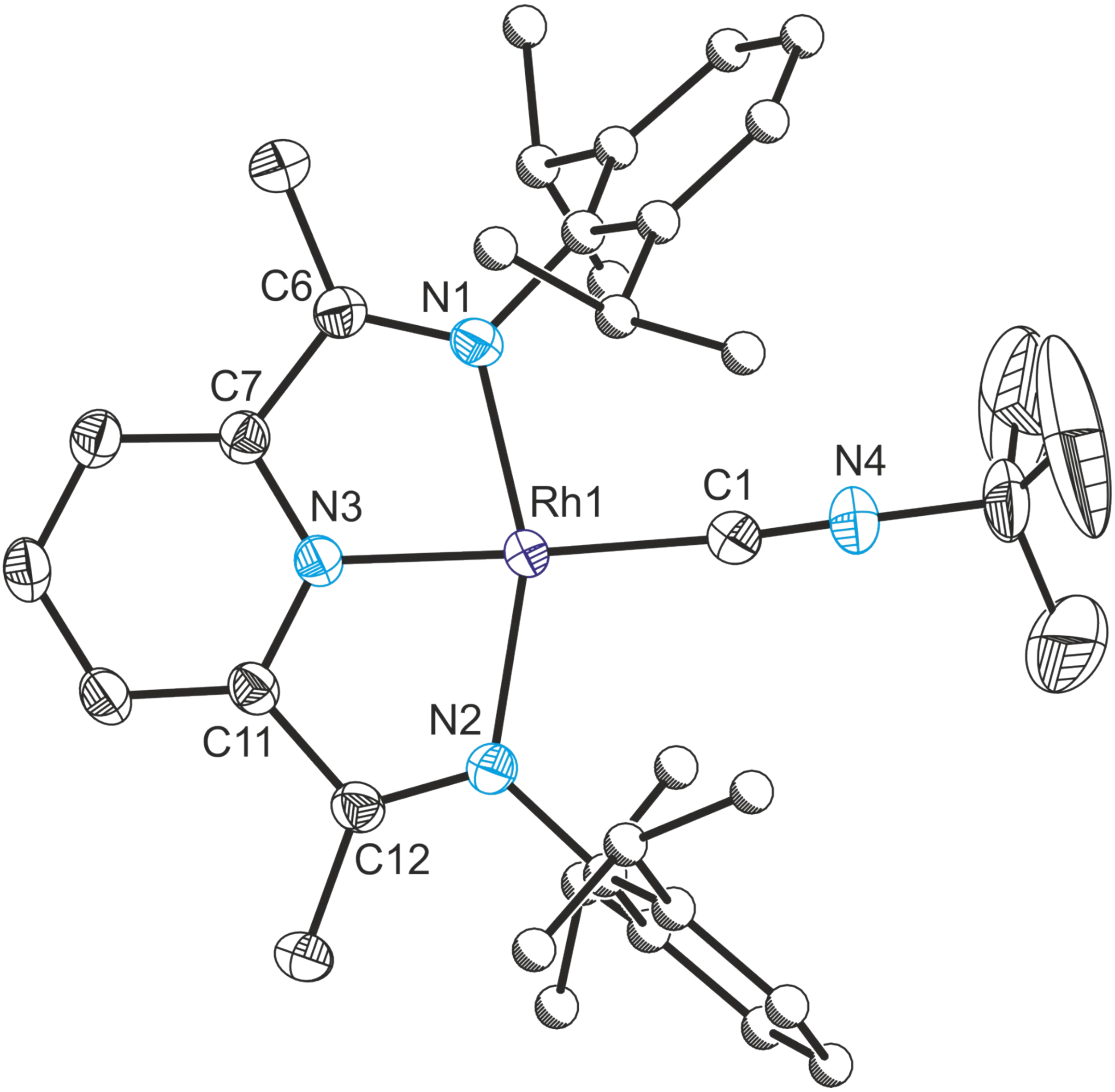

Figure S42. Single crystal X-ray structure of $\mathbf{Rh_{CNBu}^{+}}$. Thermal ellipsoids displayed at 50% probability. Atoms of Dipp groups pictured as spheres of arbitrary radius. Hydrogen atoms have been removed for clarity. Selected bond distances (Å) and angles (°): Rh1–C1 1.954(2), C1–N4 1.150(3), Rh1–N1 2.0310(18), Rh1–N2 2.0417(18), Rh1–N3 1.9422(17), N1–C6 1.306(3), C6–C7 1.475(3), C7–N3 1.348(3), N3–C11 1.345(3), C11–C12 1.479(3), C12–N2 1.301(3); C1-Rh1-N1 99.45(8), C1-Rh1-N2 102.87(8), C1-Rh1-N3 174.74(9), N1-Rh1-N2 157.60(7), N1-Rh1-N3 78.67(7), N2-Rh1-N3, 78.93(7).

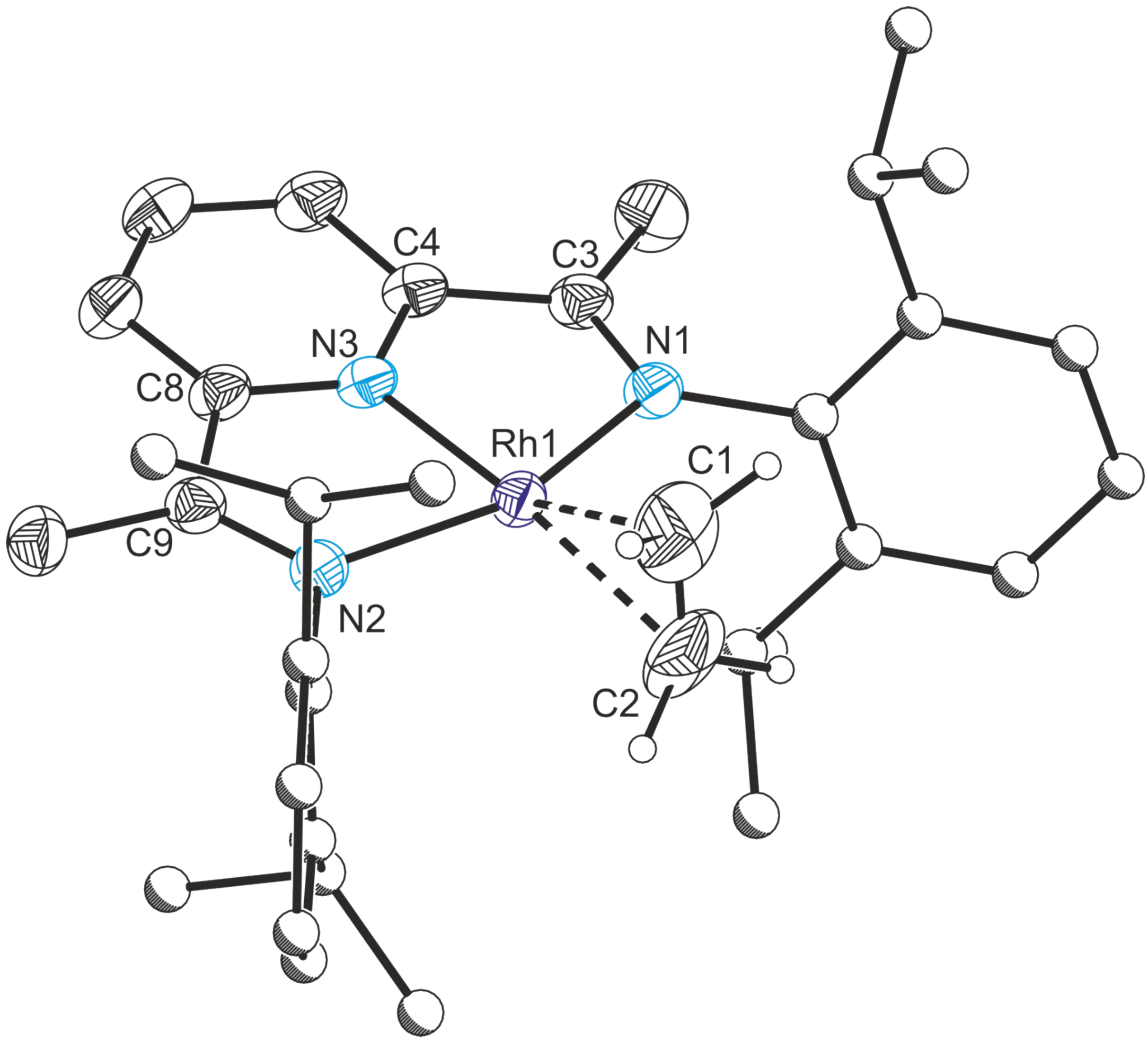

Figure S43. Single crystal X-ray structure of **Rh_{ethene}^{+}**. Thermal ellipsoids displayed at 50% probability. Atoms of Dipp groups pictured as spheres of arbitrary radius. Hydrogen atoms have been removed for clarity. Selected bond distances (Å) and angles (°): Rh1–C1 2.163(4), Rh1–C2 2.183(3), C1–C2 1.301(6), Rh1–N1 2.057(2), Rh1–N2 2.056(2), Rh1–N3 1.960(2), N1–C3 1.306(3), C3–C4 1.473(4), C4–N3 1.343(3), N3–C8 1.342(3), C8–C9 1.478(4), C9–N2 1.304(3); C1-Rh1-C2 34.84(17), C1-Rh1-N1 102.00(15), C1-Rh1-N2 100.06(15), C1-Rh1-N3 153.30(15), C2-Rh1-N1 100.71(14), C2-Rh1-N2 101.60(14), C2-Rh1-N3 171.86(14), N1-Rh1-N2 156.73(9), N1-Rh1-N3 78.51(9), N2-Rh1-N3, 78.47(9).

3. Computational details

3.1. General computational methods

Density functional theory (DFT) calculations were performed using the ORCA 5.0.2 software package.[10–12] All methods were used as implemented. Geometries were optimized using the B97-D3 functional and the def2-SVP basis set.[13,14] Analytical frequency calculations were carried out to verify all molecular geometries as true minima (N_{imag} = 0). Single point calculations were performed on all molecules and fragments using the ωB97X-D3 functional[15] and the Resolution of Identity approximation (RIJCOSX),[15,16] and corrected for relativistic effects using the zeroth order regular approximation (ZORA). The segmented all-electron relativistically contracted basis set SARC-ZORA-TZVP was used for Au and Rh, and the relativistically contracted triple-zeta basis set ZORA-def2-TZVP was used for all other atoms, along with the SARC/J auxiliary basis set.[16,17] Natural Bond Order analysis and Natural Population Analysis were carried out using the NBO 7.0 program.[18] Energy decomposition analysis was carried out using the ETS-NOCV approach[19,20] as implemented in ORCA 5.0.2, with difference densities generated by Multiwfn 3.8.[21]

3.2. Calculations on anions

Calculations on the cyanide ($C{\equiv}N^-$), acetylide ($C{\equiv}CH^-$) and cyaphide ($C{\equiv}P^-$) anions were performed using a method benchmarked against experimental and calculated electron affinities (EA) for ($C{\equiv}N^\bullet$),[22] ($C{\equiv}CH^\bullet$),[23] and ($C{\equiv}P^\bullet$)[24]: the range separated hybrid functional wB97X-D3, the zero order regular approximation (ZORA) using HF model densities, and the relativistically contracted quadruple-zeta basis set with added polarization functions ZORA-def2-QZVPP.

Table S5. Frontier orbitals of the cyanide, acetylide, and cyaphide ions ($C{\equiv}E^-$, E = N, CH, P).

	Isosurface	Reduced Loewdin Population		Energy /eV	EA($CE^\bullet$) / eV
		C	E		
$C{\equiv}N^-$ HOMO−1/HOMO−2	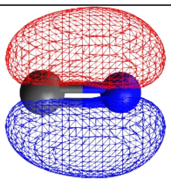	44.2%	55.8%	−4.47	
$C{\equiv}N^-$ HOMO	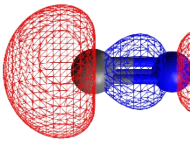	71.6%	28.4%	−3.62	3.862[22]
$C{\equiv}N^-$ LUMO/LUMO+1	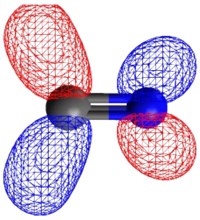	79.3%	20.7%	9.34	
$C{\equiv}CH^-$ HOMO−2	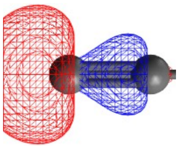	80.9%	19.1%	−3.19	
$C{\equiv}CH^-$ HOMO/HOMO−1	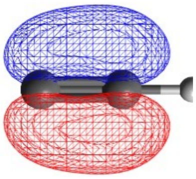	43.6%	56.4%	−3.08	2.969[23]

$C{\equiv}CH^-$ LUMO	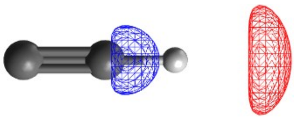	7.5%	92.5%	7.75	
$C{\equiv}CH^-$ LUMO+1/LUMO+2	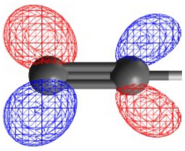	48.5%	51.5%	8.98	
$C{\equiv}P^-$ HOMO−1/HOMO−2	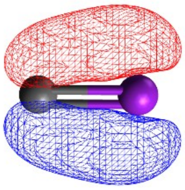	43.5%	56.5%	−3.36	
$C{\equiv}P^-$ HOMO	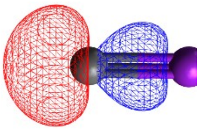	70.1%	29.9%	−3.06	2.806[24]
$C{\equiv}P^-$ LUMO/LUMO+1	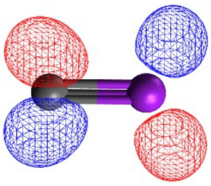	41.0%	59.0%	6.69	

3.3. Natural Bond Orbital analysis

Table S6. Summary of NBO and NPA analysis of Au(IDipp)(L) complexes, including second order perturbation energies corresponding to Au–L π back-donation ($E^{(2)}_{\pi}$).

NBO Lewis Structure	NPA Charge			Wiberg Bond Index		$E^{(2)}_{\pi}$(Au–L) / kcal mol^{-1}
	Au	C_{NHC}	C_L	C_{NHC}–Au	Au–L	
IDipp→Au–C≡P	0.42	0.14	−0.98	0.49	0.58	22.0
IDipp→Au–C≡N	0.37	0.14	−0.15	0.49	0.58	19.9
IDipp→Au–C≡N(BCF)	0.43	0.13	0.18	0.51	0.56	27.8
IDipp→Au–C≡CH	0.40	0.15	−0.46	0.50	0.58	19.2
IDipp→Au–Me	0.32	0.16	−1.12	0.47	0.61	6.2
IDipp–$Au^{\oplus}$←$Cl^{\ominus}$	0.41	0.16	–	0.60	0.45	–
IDipp–$Au^{\oplus}$←$OH^{\ominus}$	0.48	0.18	–	0.64	0.42	–

Table S7. Summary of NBO and NPA analysis of Rh(^{Dipp}PDI)(L) complexes, including second order perturbation energies corresponding to Rh–L π back-donation ($E^{(2)}_{\pi}$).

NBO Lewis Structure	NPA Charge		Wiberg Bond Index		$E^{(2)}_{\pi}$(Rh–L) / kcal mol^{-1}
	Rh	(^{Dipp}PDI)	Σ(N_{PDI}–Rh)	Rh–L	
(^{Dipp}PDI)→Rh–C≡P	0.23	0.43	1.19	0.73	44.1
(^{Dipp}PDI)→Rh–C≡N	0.21	0.43	1.26	0.63	28.7
(^{Dipp}PDI)→Rh–C≡CH	0.26	0.34	1.29	0.63	26.0
(^{Dipp}PDI)→Rh–Me	0.40	0.02	1.39	0.62	4.7
(^{Dipp}PDI)→$Rh^{\oplus}$–C≡O	0.21	0.66	1.14	0.93	78.1
(^{Dipp}PDI)→$Rh^{\oplus}$–C≡N–^{t}Bu	0.21	0.56	1.19	0.72	52.5
(^{Dipp}PDI)→$Rh^{\oplus}$←‖	0.37	0.61	1.26	0.66	–
(^{Dipp}PDI)→$Rh^{\oplus}$←$Cl^{\ominus}$	0.36	0.32	1.46	0.39	–

3.4. Energy decomposition analysis

Table S8. Summary of EDA-NOCV analysis of the M–L bond in Au(IDipp)(L) and Rh(^{Dipp}PDI)(L) complexes, including ETS-NOCV energies from orbital contributions due to σ donation (ΔE_{orb}) and π back-donation (ΔE_{orb}), in kcal mol^{-1}.

	ΔE_{tot}	ΔE_{steric}	ΔE_{disp}	ΔE_{oi}	ΔE_{orb}	ΔE_{orb}
IDippAu–C≡P	−111.6	5.4	−31.4	−85.6	−44.5	−18.0
IDippAu–C≡P / Ni(^{Me}I^{i}Pr)$_2$	−126.3	54.3	−77.8	−102.8	−51.0	−17.5
IDippAu–C≡P / Rh(Cp*)(PMe$_3$)	−101.5	51.7	−59.5	−93.7	−46.9	−17.2
IDippAu–C≡P(W(CO)$_5$) / Rh(Cp*)(PMe$_3$)	−109.2	54.9	−71.4	−92.7	−47.0	−18.4
IDippAu–C≡N	−101.0	2.9	−31.0	−72.8	−40.8	−12.3
IDippAu–C≡N(BCF)	−73.9	50.1	−54.1	−69.8	−35.1	−19.1
IDippAu–C≡CH	−107.7	4.2	−31.1	−80.8	−42.5	−12.8
IDippAu–Me	−107.7	4.9	−30.7	−81.9	−50.5	−8.3
IDippAu–Cl	−91.1	7.4	−31.0	−67.5	−38.4	−5.6
IDippAu–OH	−113.9	1.9	−30.5	−85.3	−46.0	−6.5
^{Dipp}PDIRh–C≡P	−108.1	28.8	−39.4	−97.4	−46.5	−21.5
^{Dipp}PDIRh–C≡N	−98.4	15.5	−38.1	−75.8	−38.3	−11.1
^{Dipp}PDIRh–C≡CH	−113.2	12.5	−38.4	−87.3	−41.6	−13.5
^{Dipp}PDIRh–Me	−106.8	13.7	−38.6	−81.9	−50.5	−8.3
[^{Dipp}PDIRh–C≡O]$^+$	−50.2	67.5	−37.6	−80.1	−31.1	−42.2
[^{Dipp}PDIRh–C≡N–^{t}Bu]$^+$	−62.7	50.0	−46.9	−65.8	−32.2	−25.5
[^{Dipp}PDIRh–‖]$^+$	−43.6	53.6	−39.8	−57.4	−22.3	−25.0
^{Dipp}PDIRh–Cl	−92.4	10.7	−38.0	−65.1	−28.2	−5.5

$\Delta E_{tot} = \Delta E_{steric} + \Delta E_{disp} + \Delta E_{oi} = \Delta E_{steric} + \Delta E_{disp} + \Sigma\Delta E_{orb}$

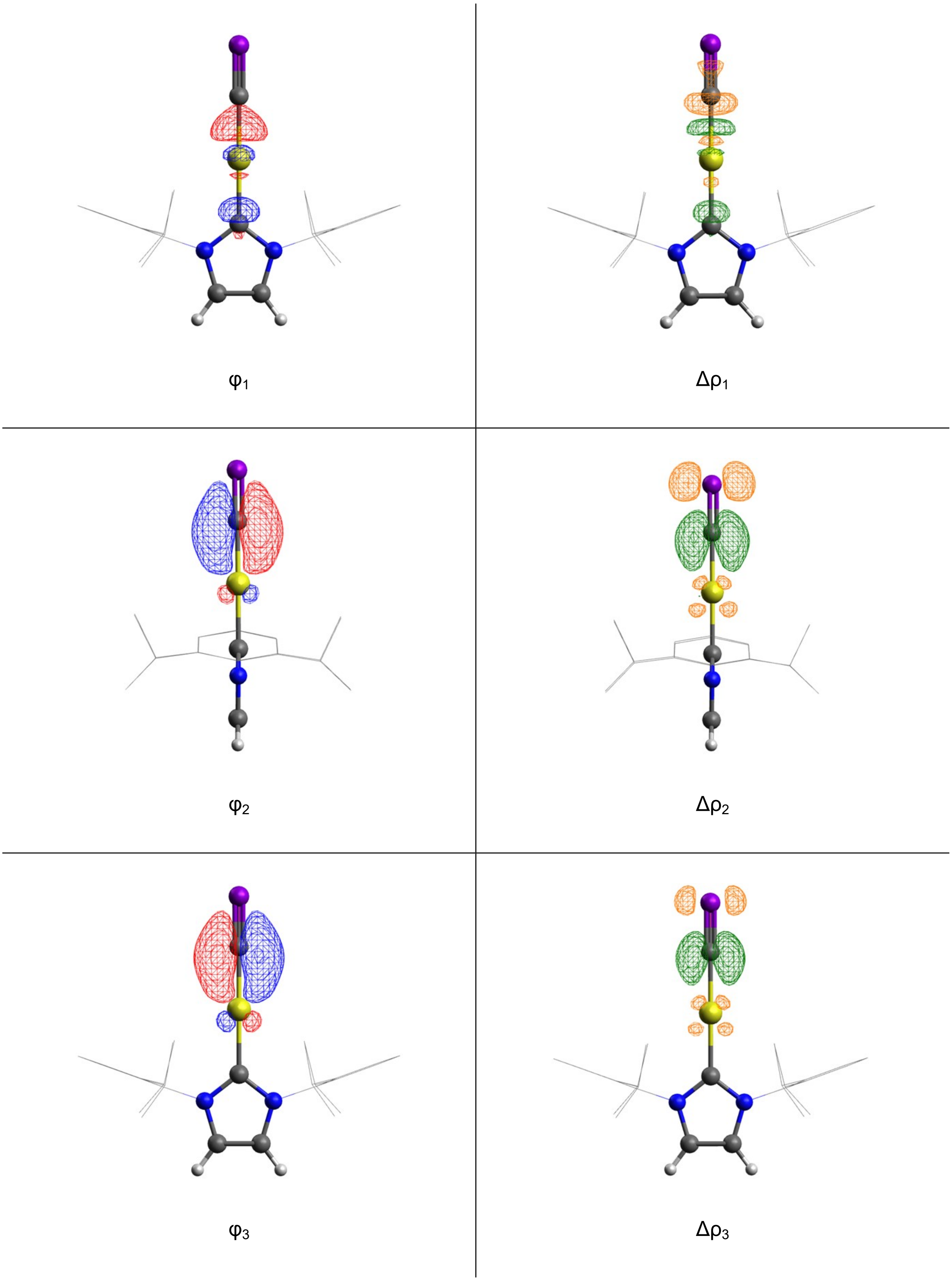

Figure S44. Contours of NOCVs φ_1, φ_2, and φ_3, ($\nu > 0$), as well as their contributions to the deformation density, $\Delta\rho_1$, $\Delta\rho_2$, and $\Delta\rho_3$, (green: $\Delta\rho>0$, orange: $\Delta\rho<0$), for the Au–CP interaction in Au(IDipp)(CP). φ_1 corresponds to σ donation, whereas φ_2 and φ_3 correspond to π back-donation.

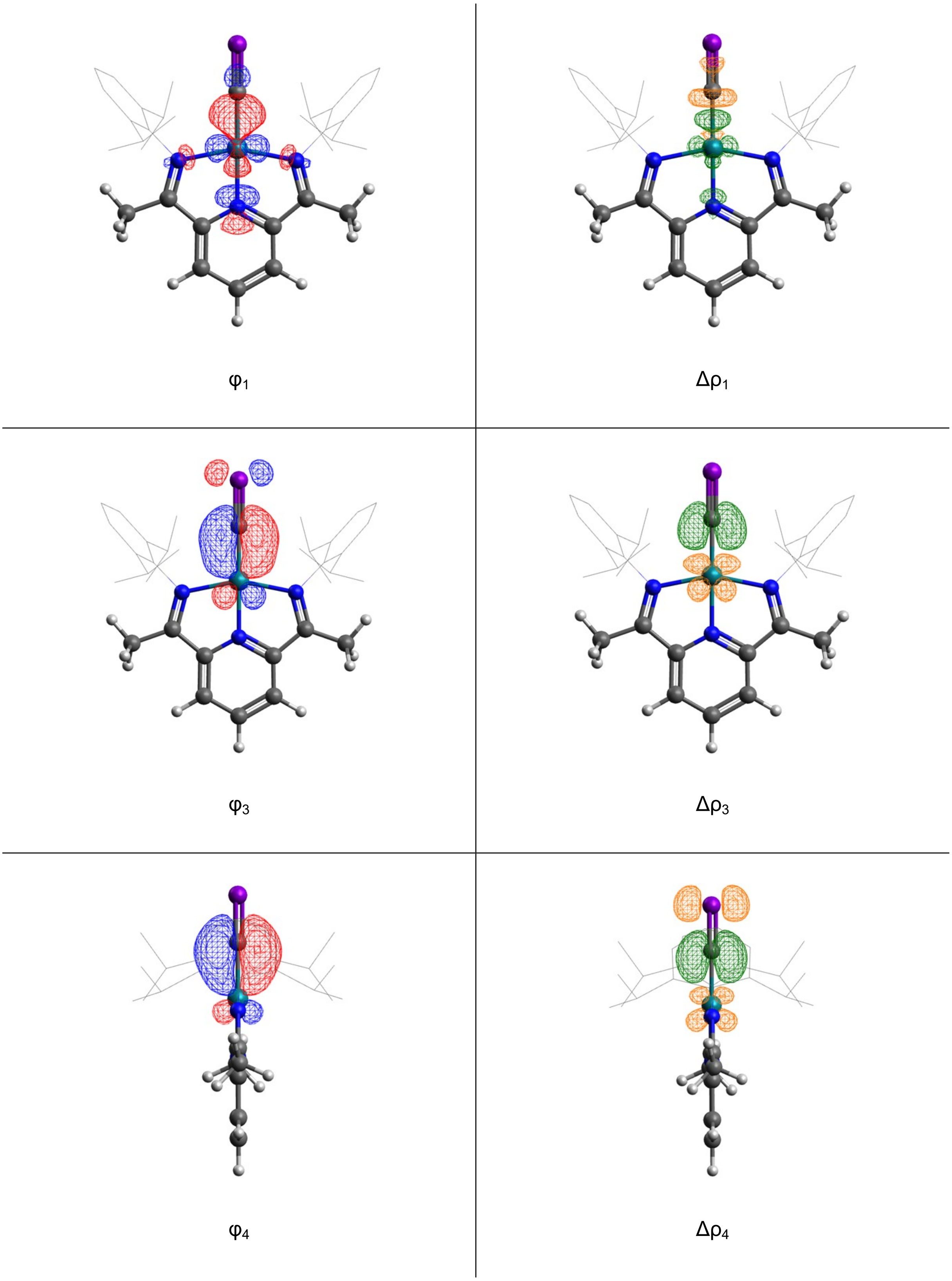

Figure S45. Contours of NOCVs φ_1, φ_3, and φ_4, ($v > 0$), as well as their contributions to the deformation density, $\Delta\rho_1$, $\Delta\rho_3$, and $\Delta\rho_4$, (green: $\Delta\rho>0$, orange: $\Delta\rho<0$), for the Rh–CP interaction in Rh(^{Dipp}PDI)(CP). φ_1 corresponds to σ donation, whereas φ_3 and φ_4 correspond to π back-donation.

3.5. Data analysis

Table S9. $^{13}C_{NHC}$ NMR shifts and C_{NHC}–Au bond lengths for Au(IDipp)(L) complexes.

L	$^{13}C_{NHC}$ / ppm	C_{NHC}–Au / Å	Ref.
CP^-	193.0	2.035(6)	6
$(\mu\text{-CP})\{Ni(^{Me}I^{i}Pr)_2\}^-$	198.5	2.024(3)	25
$(\mu\text{-CP})\{Rh(Cp^*)(PMe_3)\}^-$	195.6	2.026(4)	25
$(\mu_3\text{-CP})\{Rh(Cp^*)(PMe_3)\}\{W(CO)_5\}^-$	193.9	2.025(4)	25
CN^-	186.2	2.00(2)	26
$(\mu\text{-CN})(BCF)^-$	183.1	2.005(3)	25
$CCPh^-$	190.9	2.018(7)	26
$BPin^-$	216.7	2.084(4)	27
H^-	204.9	2.045(3)	28
Me^-	201.4	2.038(5)	29
OH^-	171.9	1.935(9)	26
Cl^-	175.1	1.9394(1)	30,31
CO	174.6	1.971(5)	32
CNXyl	178.6	–	33
IDipp	184.2	2.025(7)	34
P^tBu_3	191.9	2.045(7)	34
PPh_3	188.2	2.039(5)	34
Py	167.1	1.961(7)	35
MeCN	166.0	1.974(2)	35

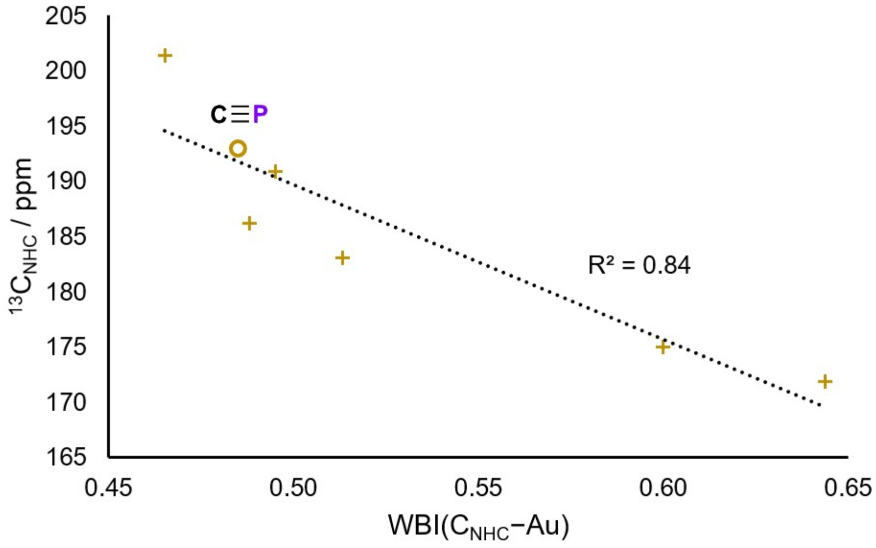

Figure 46. Calculated C_{NHC}–Au bond order (Wiberg) plotted against experimental $^{13}C_{NHC}$ NMR shifts for selected Au(IDipp)(L) complexes.

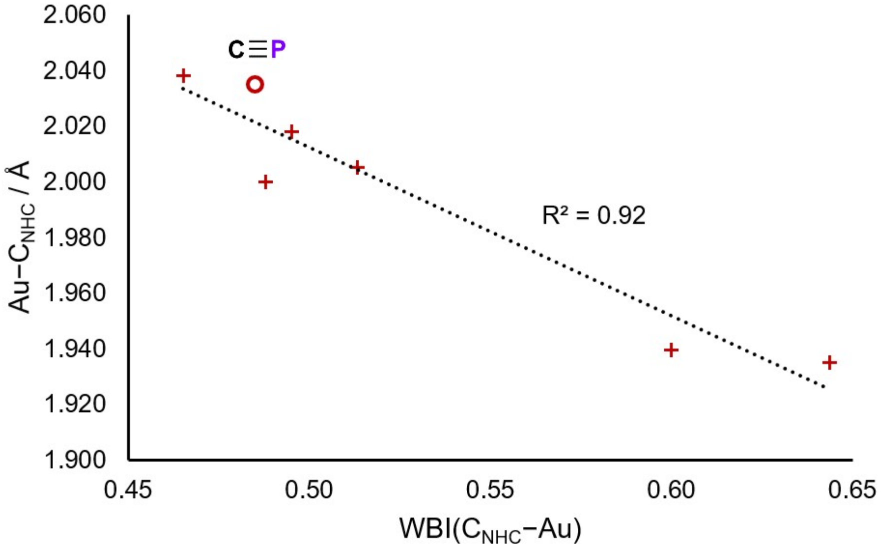

Figure S47. Calculated C_{NHC}–Au bond order (Wiberg) plotted against experimental solid-state C_{NHC}–Au bond lengths for selected Au(IDipp)(L) complexes.

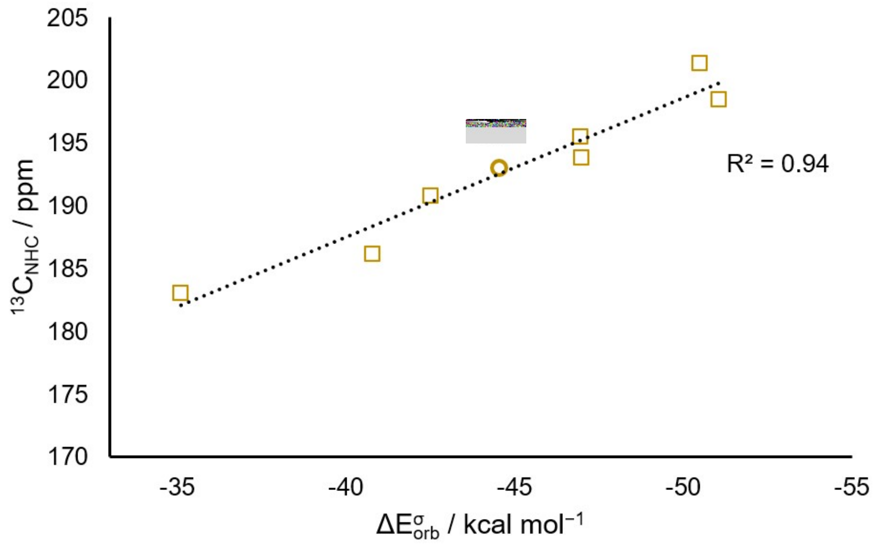

Figure S48. Calculated L–Au σ donation energies (ETS-NOCV, ΔE_{orb}) plotted against experimental $^{13}C_{NHC}$ NMR shifts for selected Au(IDipp)(L) complexes.

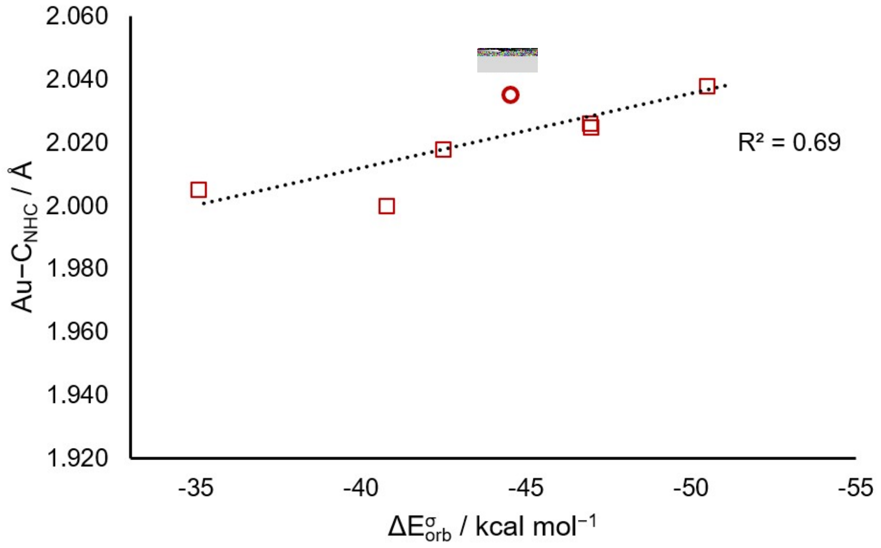

Figure S49. Calculated L–Au σ donation energies (ETS-NOCV, ΔE_{orb}) plotted against experimental solid-state C_{NHC}–Au bond lengths for selected Au(IDipp)(L) complexes.

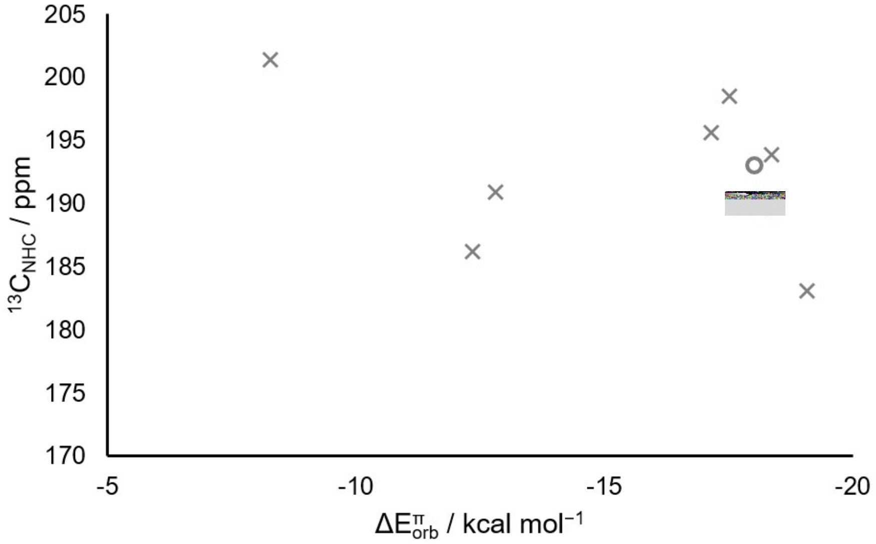

Figure S50. Calculated Au–L π back-donation energies (ETS-NOCV, ΔE_{orb}) plotted against experimental $^{13}C_{NHC}$ NMR shifts for selected Au(IDipp)(L) complexes.

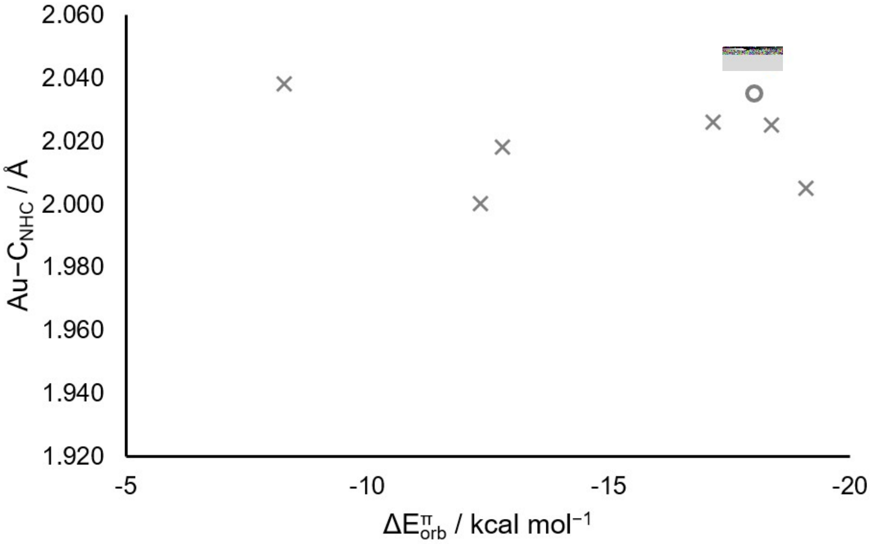

Figure S51. Calculated Au–L π back-donation energies (ETS-NOCV, ΔE_{orb}) plotted against experimental solid-state C_{NHC}–Au bond lengths for selected Au(IDipp)(L) complexes.

Table S10. δ(PDI) values and N_{py}–Co bond lengths for Co(^{Dipp}PDI)(L) complexes.

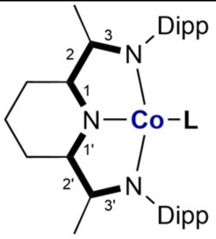

$$\delta(\text{PDI}) = \left(\frac{d2 + d2'}{2}\right) - \left(\frac{d1 + d1' + d3 + d3'}{4}\right)$$

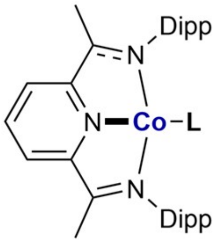

L	δ(PDI)*	N_{py}–Co / Å	Ref.
CP^{-}	0.131(9)	1.825(3)	6
CCH^{-}	0.098(7)	1.812(2)	–
Me^{-}	0.090(9)	1.834(3)	36
N_2	0.132(9)	1.812(3)	3
CO	0.144(7)	1.847(2)	–
CN^{t}Bu	0.121(4)	1.819(2)	–
Cl^{-}	0.096(12)	1.798(3)	3

*Uncertainties were determined by error propagation, where

$$\sigma(\delta(\text{PDI})) = \sqrt{(\frac{\sigma(d2)}{2})^2 + (\frac{\sigma(d2')}{2})^2 - (\frac{\sigma(d1)}{4})^2 - (\frac{\sigma(d1')}{4})^2 - (\frac{\sigma(d3)}{4})^2 - (\frac{\sigma(d3')}{4})^2}$$

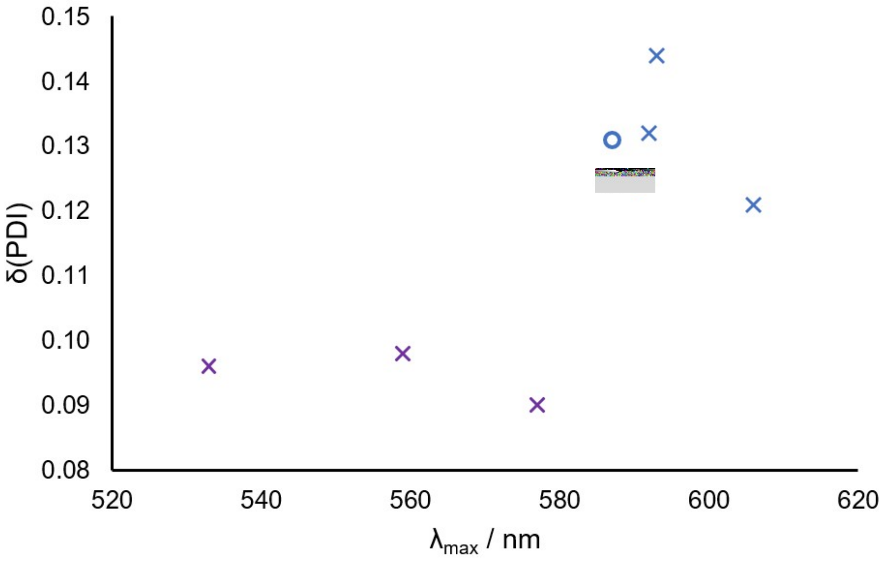

Figure S52. Experimental UV-vis absorption maxima (λ_{max}) plotted against experimental δ(PDI) values for Co(^{Dipp}PDI)(L) complexes.

Table S11. δ(PDI) values and N_{py}–Rh bond lengths for Rh(^{Dipp}PDI)(L) complexes.

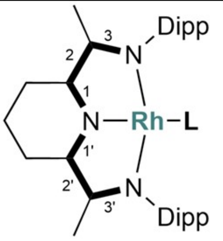

$$\delta(\text{PDI}) = \left(\frac{d2 + d2'}{2}\right) - \left(\frac{d1 + d1' + d3 + d3'}{4}\right)$$

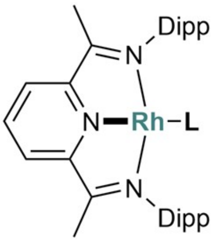

L	δ(PDI)*	N_{py}–Rh / Å
CP^-	0.134(3)	1.943(2)
CCH^-	0.124(2)	1.926(2)
Me^-	0.110(6)	1.936(3)
CO	0.161(3)	1.969(2)
CN^tBu	0.152(3)	1.942(3)
C_2H_4	0.152(3)	1.960(2)
Cl	0.108(6)	1.898(4)

*Uncertainties were determined by error propagation, where $\sigma(\delta(\text{PDI})) = \sqrt{(\frac{\sigma(d2)}{2})^2 + (\frac{\sigma(d2')}{2})^2 - (\frac{\sigma(d1)}{4})^2 - (\frac{\sigma(d1')}{4})^2 - (\frac{\sigma(d3)}{4})^2 - (\frac{\sigma(d3')}{4})^2}$

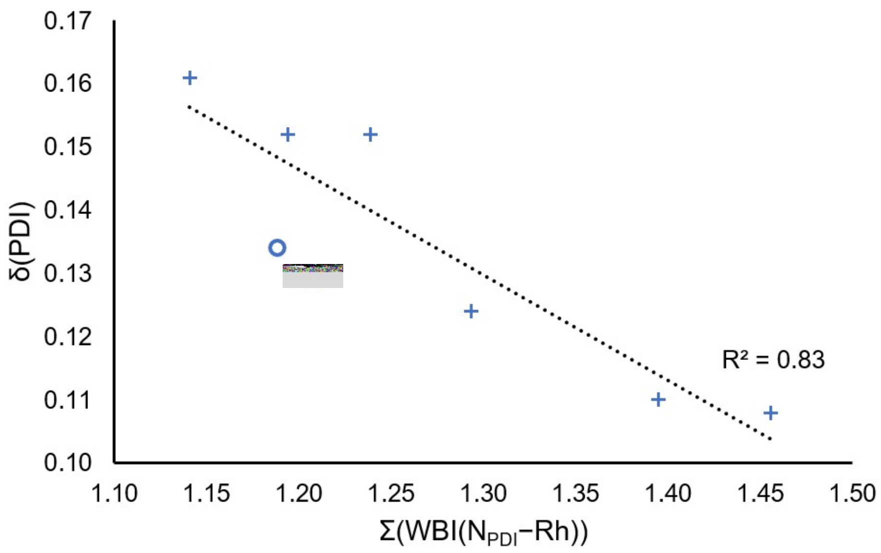

Figure S53. Calculated N_{PDI}–Rh bond orders (Wiberg) plotted against experimental δ(PDI) values for selected Rh(^{Dipp}PDI)(L) complexes.

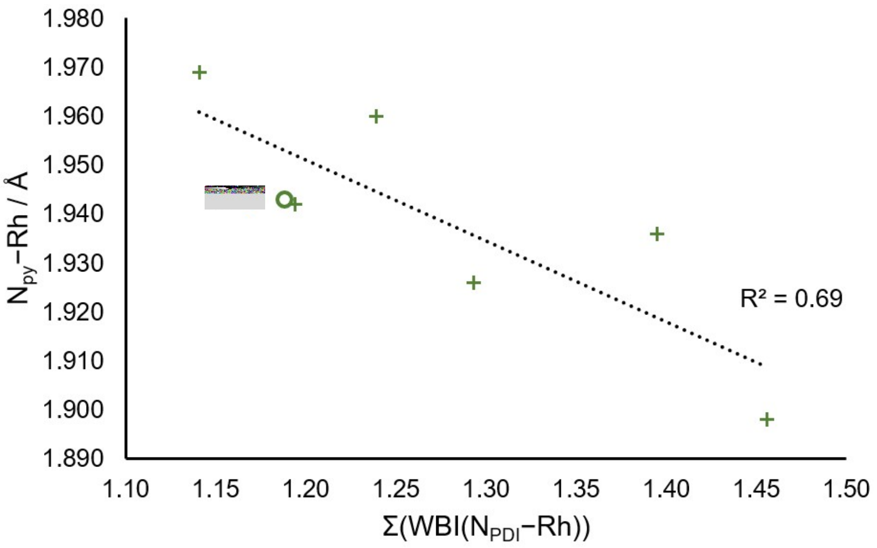

Figure S54. Calculated N_{PDI}–Rh bond orders (Wiberg) plotted against experimental solid-state N_{py}–Rh bond lengths for selected Rh(^{Dipp}PDI)(L) complexes.

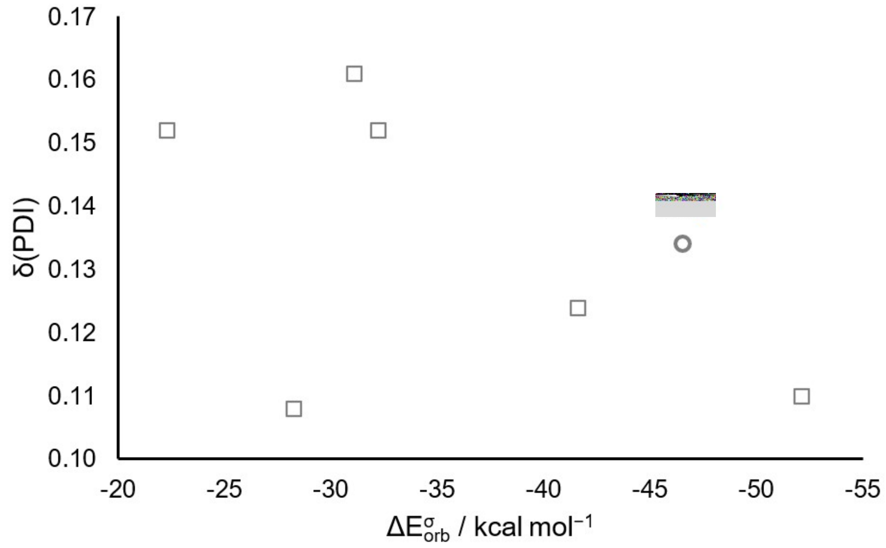

Figure S55. Calculated L–Rh σ donation energies (ETS-NOCV, ΔE_{orb}) plotted against experimental δ(PDI) values for selected Rh(^{Dipp}PDI)(L) complexes.

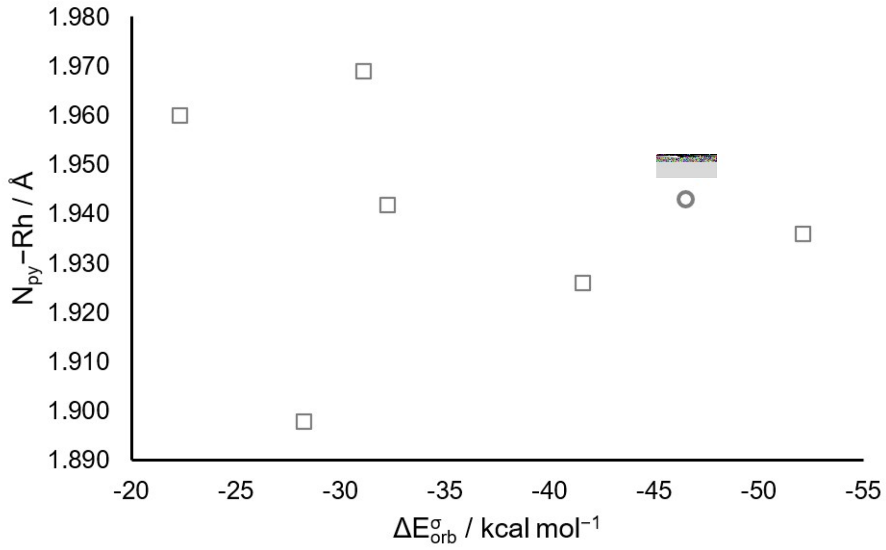

Figure S56. Calculated L–Rh σ donation energies (ETS-NOCV, ΔE_{orb}) plotted against experimental solid-state N_{py}–Rh bond lengths for selected Rh(^{Dipp}PDI)(L) complexes.

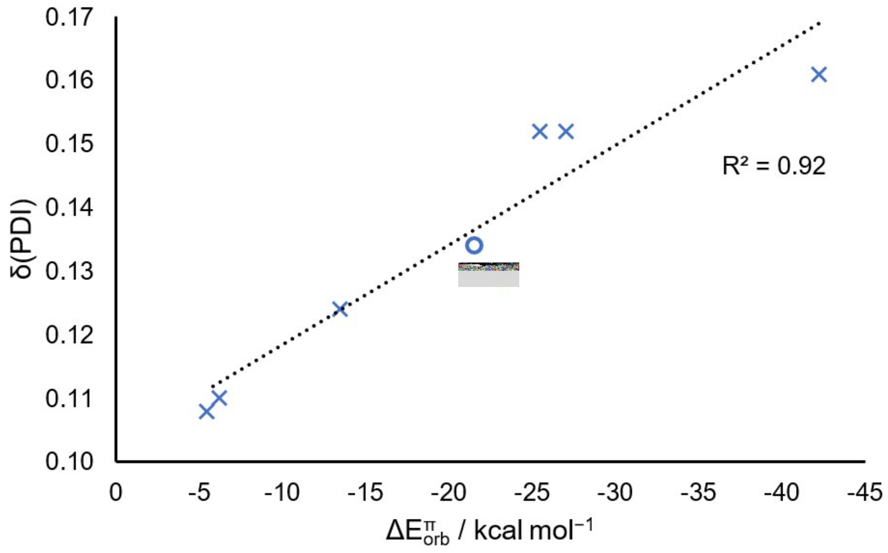

Figure S57. Calculated Rh–L π back-donation energies (ETS-NOCV, ΔE_{orb}) plotted against experimental δ(PDI) values for selected Rh(^{Dipp}PDI)(L) complexes.

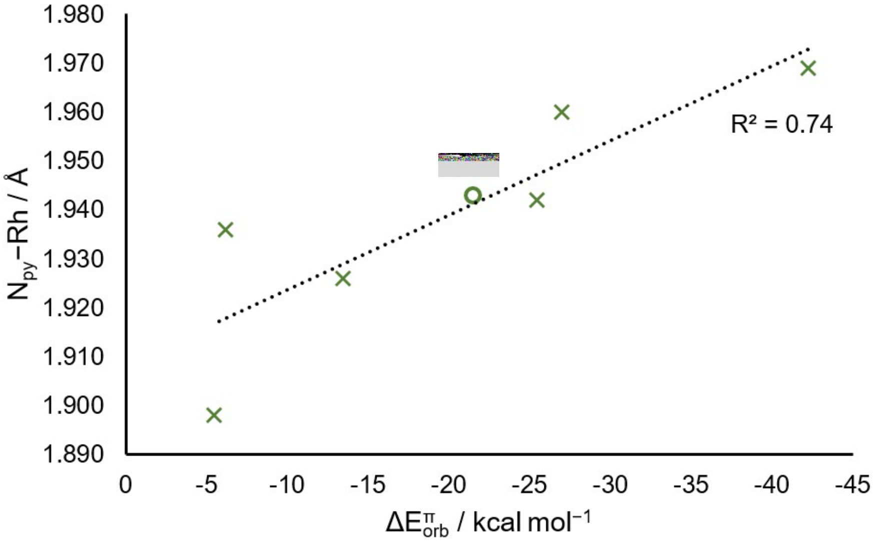

Figure S58. Calculated Rh–L π back-donation energies (ETS-NOCV, ΔE_{orb}) plotted against experimental solid-state N_{py}–Rh bond lengths for selected Rh(^{Dipp}PDI)(L) complexes.

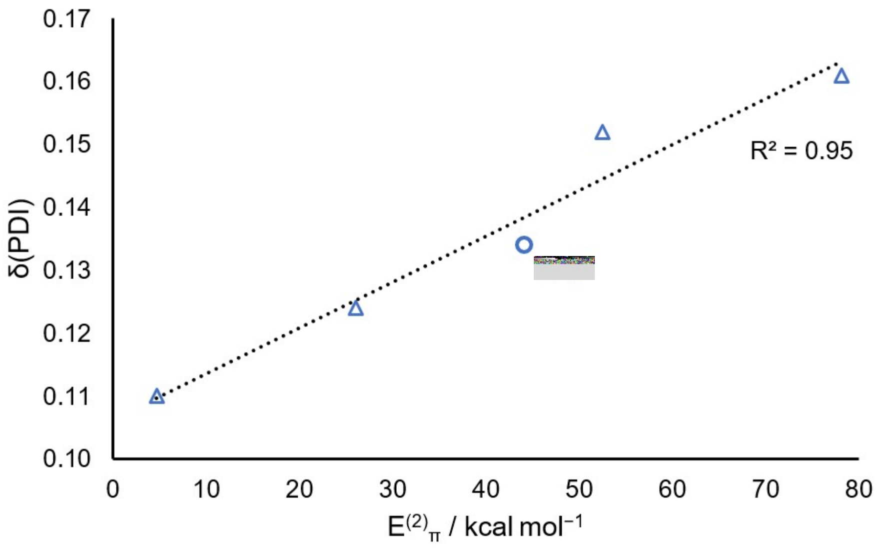

Figure S59. Calculated Rh–L π back-donation energies (NBO, $E^{(2)}_{\pi}$) plotted against experimental δ(PDI) values for selected Rh(^{Dipp}PDI)(L) complexes.

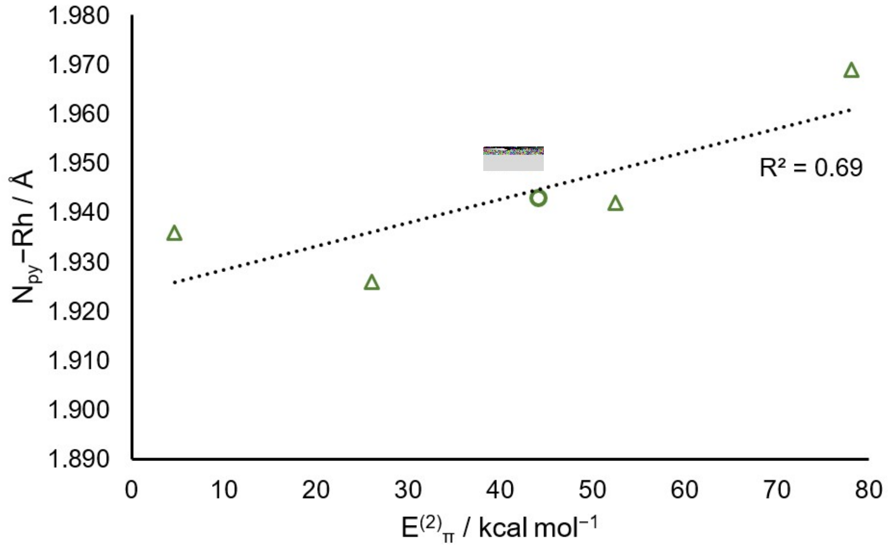

Figure S60. Calculated Rh–L π back-donation energies (NBO, $E^{(2)}_{\pi}$) plotted against experimental solid-state N_{py}–Rh bond lengths for selected Rh(^{Dipp}PDI)(L) complexes.

4. References

1 A. J. Martínez-Martínez and A. S. Weller, *Dalton Trans.*, 2019, **48**, 3551–3554.

2 C. C. Hojilla Atienza, C. Milsmann, E. Lobkovsky and P. J. Chirik, *Angew. Chem. Int. Ed.*, 2011, **50**, 8143–8147.

3 V. C. Gibson, M. J. Humphries, K. P. Tellmann, D. F. Wass, A. J. P. White and D. J. Williams, *Chem. Commun.*, 2001, **1**, 2252–2253.

4 S. Nückel and P. Burger, *Organometallics*, 2001, **20**, 4345–4359.

5 Q. Knijnenburg, D. Hetterscheid, T. Martijn Kooistra and P. H. M. Budzelaar, *Eur. J. Inorg. Chem.*, 2004, **2004**, 1204–1211.

6 D. W. N. Wilson, S. J. Urwin, E. S. Yang and J. M. Goicoechea, *J. Am. Chem. Soc.*, 2021, ***143***, 10367–10373.

7 E. L. Dias, M. Brookhart and P. S. White, *Organometallics*, 2000, **19**, 4995–5004.

8 CrysAlisPro, Agilent Technologies, Version 1.171.41.117a.

9 (a) G. M. Sheldrick in SHELXL97, *Programs for Crystal Structure Analysis (Release 97-2)*, Institut für Anorganische Chemie der Universität, Tammanstrasse 4, D-3400 Göttingen, Germany, 1998; (b) G. M. Sheldrick, *Acta Crystallogr. Sect. A*, 1990, **46**, 467−473; (c) G. M. Sheldrick, *Acta Crystallogr. Sect. A*, 2008, 64, 112−122.

10 F. Neese, *Wiley Interdiscip. Rev. Comput. Mol. Sci.*, 2012, **2**, 73–78.

11 F. Neese, *Wiley Interdiscip. Rev. Comput. Mol. Sci.*, 2018, **8**, 1–6.

12 F. Neese, F. Wennmohs, U. Becker and C. Riplinger, *J. Chem. Phys.*, 2020, **152**, 224108.

13 S. Grimme, *J. Comput. Chem.*, 2006, **27**, 1787–1799.

14 F. Weigend and R. Ahlrichs, *Phys. Chem. Chem. Phys.*, 2005, **7**, 3297–3305.

15 Y. S. Lin, G. De Li, S. P. Mao and J. Da Chai, *J. Chem. Theory Comput.*, 2013, ***9***, 263–272.

16 D. A. Pantazis, X. Y. Chen, C. R. Landis and F. Neese, *J. Chem. Theory Comput.*, 2008, **4**, 908–919.

17 M. Bühl, C. Reimann, D. A. Pantazis, T. Bredow and F. Neese, *J. Chem. Theory Comput.*, 2008, **4**, 1449–1459.

18 E. D. Glendening, J. K. Badenhoop, A. E. Reed, J. E. Carpenter, J. A. Bohmann, C. M. Morales, P. Karafiloglou, C. R. Landis and F. Weinhold, *NBO 7.0*, Theoretical Chemistry Institute, University of Wisconsin, Madison, 2018.

19 M. Mitoraj and A. Michalak, *J. Mol. Model.*, 2007, ***13***, 347–355.

20 L. Zhao, M. von Hopffgarten, D. M. Andrada and G. Frenking, *Wiley Interdiscip. Rev.Comput. Mol. Sci.*, 2018, **8**, e1345.

21 T. Lu and F. Chen, *J. Comput. Chem.*, 2012, **33**, 580–592.

22 S. E. Bradforth, E. H. Kim, D. W. Arnold and D. M. Neumark, *J. Chem. Phys.*, 1998, **98**,

800–810.

23 J. Zhou, E. Garand and D. M. Neumark, *J. Chem. Phys.*, 2007, **127**, 114313.

24 Russell D. Johnson III, *NIST Computational Chemistry Comparison and Benchmark DataBase (Release 22): NIST Standard Reference Database Number 101* can be found under http://cccbdb.nist.gov/, **2022**.

25 E. S. Yang and J. M. Goicoechea, *Angew. Chem. Int. Ed.*, 2022, **61**, e202206783.

26 S. Gaillard, A. M. Z. Slawin and S. P. Nolan, *Chem. Commun.*, 2010, **46**, 2742–2744.

27 C. M. Zinser, F. Nahra, L. Falivene, M. Brill, D. B. Cordes, A. M. Z. Slawin, L. Cavallo, C. S. J. Cazin and S. P. Nolan, *Chem. Commun.*, 2019, **55**, 6799–6802.

28 E. Y. Tsui, P. Müller and J. P. Sadighi, *Angew. Chem. Int. Ed.*, 2008, **47**, 8937–8940.

29 V. J. Scott, J. A. Labinger and J. E. Bercaw, *Organometallics*, 2010, **29**, 4090–4096.

30 M. R. Fructos, T. R. Belderrain, P. de Frémont, N. M. Scott, S. P. Nolan, M. Mar Díaz-Requejo and P. J. Pérez, *Angew. Chem. Int. Ed.*, 2005, **44**, 5284–5288.

31 R. E. Marsh, *Acta Crystallogr. Sect. B Struct. Sci.*, 2009, **65**, 782–783.

32 C. Dash, P. Kroll, M. Yousufuddin and H. V. R. Dias, *Chem. Commun.*, 2011, **47**, 4478–4480.

33 L. Canovese, F. Visentin, C. Levi and V. Bertolasi, *Organometallics*, 2011, **30**, 875–883.

34 S. Gaillard, P. Nun, A. M. Z. Slawin and S. P. Nolan, *Organometallics*, 2010, **29**, 5402–5408.

35 P. de Frémont, N. Marion and S. P. Nolan, *J. Organomet. Chem.*, 2009, **694**, 551–560.

36 M. J. Humphries, K. P. Tellmann, V. C. Gibson, A. J. P. White and D. J. Williams, *Organometallics*, 2005, 24, 2039–2050.
